# Exploring the potential of bis(thiazol-5-yl)phenylmethane derivatives as novel candidates against genetically defined multidrug-resistant *Staphylococcus aureus*

**DOI:** 10.1371/journal.pone.0300380

**Published:** 2024-03-22

**Authors:** Povilas Kavaliauskas, Waldo Acevedo, Andrew Garcia, Ethan Naing, Birute Grybaite, Birute Sapijanskaite-Banevic, Ramune Grigaleviciute, Ruta Petraitiene, Vytautas Mickevicius, Vidmantas Petraitis

**Affiliations:** 1 Division of Infectious Diseases, Department of Medicine, Weill Cornell Medicine of Cornell University, New York, NY, United States of America; 2 Institute of Infectious Diseases and Pathogenic Microbiology, Prienai, Lithuania; 3 Biological Research Center, Lithuanian University of Health Sciences, Kaunas, Lithuania; 4 Department of Organic Chemistry, Kaunas University of Technology, Kaunas, Lithuania; 5 Instituto de Química, Facultad de Ciencias, Pontificia Universidad Católica de Valparaíso, Valparaíso, Chile; 6 Department of Animal Nutrition, Lithuanian University of Health Sciences, Kaunas, Lithuania; 7 Center for Discovery and Innovation, Hackensack Meridian Health, Nutley, New Jersey, United States of America; Kafrelsheikh University Faculty of Pharmacy, EGYPT

## Abstract

Antimicrobial resistance (AMR) represents an alarming global challenge to public health. Infections caused by multidrug-resistant *Staphylococcus aureus* (*S*. *aureus*) pose an emerging global threat. Therefore, it is crucial to develop novel compounds with promising antimicrobial activity against *S*. *aureus* especially those with challenging resistance mechanisms and biofilm formation. Series of bis(thiazol-5-yl)phenylmethane derivatives were evaluated against drug-resistant Gram-positive bacteria. The screening revealed an *S*. *aureus*-selective mechanism of bis(thiazol-5-yl)phenylmethane derivatives (MIC 2–64 *μ*g/mL), while significantly lower activity was observed with vancomycin-resistant *Enterococcus faecalis* (MIC 64 *μ*g/mL) (*p<*0.05). The most active phenylmethane-based (p-tolyl) derivative, **23a,** containing nitro and dimethylamine substituents, and the naphthalene-based derivative, **28b,** harboring fluorine and nitro substituents, exhibited strong, near MIC bactericidal activity against *S*. *aureus* with genetically defined resistance phenotypes such as MSSA, MRSA, and VRSA and their biofilms. The *in silico* modeling revealed that most promising compounds **23a** and **28b** were predicted to bind *S*. *aureus* MurC ligase. The **23a** and **28b** formed bonds with MurC residues at binding site, specifically Ser12 and Arg375, indicating consequential interactions essential for complex stability. The *in vitro* antimicrobial activity of compound **28b** was not affected by the addition of 50% serum. Finally, all tested bis(thiazol-5-yl)phenylmethane derivatives showed favorable cytotoxicity profiles in A549 and THP-1-derived macrophage models. These results demonstrated that bis(thiazol-5-yl)phenylmethane derivatives **23a** and **28b** could be potentially explored as scaffolds for the development of novel candidates targeting drug-resistant *S*. *aureus*. Further studies are also warranted to understand *in vivo* safety, efficacy, and pharmacological bioavailability of bis(thiazol-5-yl)phenylmethane derivatives.

## Introduction

Antimicrobial resistance (AMR) represents an alarming global challenge to public health. The escalating emergence of resistance, particularly against last-line antibiotics, poses a significant threat, leading to increased mortality rates and escalating treatment costs [1–3]. Annually, an estimated 17 million individuals succumb to bacterial infections, with over 2 million individuals are affected by drug-resistant strains [4]. The rapid spread of AMR underscores the urgent need for comprehensive and novel strategies targeting drug-resistance among clinically important pathogens.

The need for novel antimicrobial agents with unique mechanisms of action is particularly evident in the case of Gram-positive pathogens, especially multidrug-resistant (MDR) *Staphylococcus aureus* (*S*. *aureus*) [5, 6]. This becomes more evident in infections caused by methicillin-resistant *S*. *aureus* (MRSA) or vancomycin-resistant (VRSA) *S*. *aureus*, particularly those cases with surgically implanted medical devices like prostheses or catheters, which require assertive treatment strategies often involving surgical removal [7, 8]. Additionally, the ability of *S*. *aureus* to form biofilms is associated with prolonged and persistent infections, demanding extended treatment durations [8, 9]. Hence, there is a growing need for novel antimicrobial compounds exhibiting activity that effectively targets both bacteria and their biofilms [10].

Infections caused by *S*. *aureus* exhibit considerable heterogeneity in their nature, necessitating a multifaceted treatment approach that encompasses the administration of staphylococci-active antibiotics alongside surgical debridement, removal of indwelling devices or aggressive wound management [11–14]. Several antibiotics with established clinical efficacy are deployed to combat staphylococcal infections, targeting crucial bacterial processes including cell wall biosynthesis, transcription, translation, and DNA replication [10, 11, 14]. Resistance to antibiotics in *S*. *aureus* emerges through various mechanisms, including modification of the drug target, enzymatic inactivation of the drug, enhanced drug efflux, and the dissemination of mobile genetic elements that facilitate resistance development [14, 15]. Notably, the rapidly growing antibiotic resistance poses an increasing challenge, particularly with respect to vancomycin, a crucial antibiotic employed in the treatment of drug-resistant staphylococcal infections [15]. Although the occurrence of VRSA or vancomycin-intermediate *S*. *aureus* (VISA) strains remains relatively rare, its prevalence is on the rise [15]. Consequently, it is crucial to develop novel compounds capable of combatting *S*. *aureus* strains harboring diverse pre-existing resistance mechanisms.

Thiazole, a five-membered heterocyclic ring containing both sulfur and nitrogen, has emerged as a versatile scaffold in the development of various antimicrobial drugs [16]. Thiazole inherent chemical properties, such as reactive and electron-rich structure, lend themselves well to various substitutions, allowing the generation of diverse derivatives with enhanced bioactivity. Moreover, thiazole derivatives, often has moderate lipophilicity and the potential for hydrogen bonding, contribute to favorable pharmacokinetic profiles [16, 17]. Thiazole derivatives have displayed efficacy against a various pathogenic organism, including WHO priority listed pathogens [16, 17]. Depending on the structural substitutions, these compounds often target essential microbial processes such as DNA synthesis, protein translation, and direct and indirect cell wall biosynthesis [16, 18]. The sulfur atom within the thiazole ring can coordinate with metal ions crucial for microbial enzyme activity, adding another layer to their antimicrobial mechanism [18, 32]. The thiazole nucleus containing compound shows favorable biological properties with the ability to interact with diverse microbial targets including cell wall synthesis pathway, thus making thiazole-based compounds as a promising scaffold for the development of novel and effective antimicrobial agents for further pre-clinical evaluation against multidrug-resistant *S*. *aureus* or other Gram-positive pathogens [11, 14, 17, 32].

In our previous paper, we described the initial synthesis of high molecular weight bis(thiazol-5-yl)phenylmethane derivatives [18, 19]. These compounds demonstrated structure-depended antimicrobial activity against *Mycobacterium luteus* (*M*. *luteus*). As a continuation of our interest to develop novel bioactive compounds targeting multidrug-resistant pathogens, these compounds were screened using drug-resistant bacterial and fungal pathogens. In the current study, we describe the subsequent characterization of bis(thiazol-5-yl)phenylmethane as promising candidates selectively targeting *S*. *aureus* even with the presence of challenging drug-resistance mechanisms. The molecular *in silico* modeling studies reveled that most potent bis(thiazol-5-yl)phenylmethane derivatives **23a** and **28b** interacts with active center of *S*. *aureus* MurC protein, thus acting as novel modulator of MurC in *S*. *aureus*. Finally, the most promising hit compounds were able to induce the dispersion of mature *S*. *aureus* biofilms, thus making bis(thiazol-5-yl)phenylmethane as promising scaffold for further development of *S*. *aureus*-directed antimicrobial candidates.

## Materials and methods

### Synthetic procedures

Compounds **Ib**, and **6b, 9b** were resynthesized according previously described procedures [18].

Compounds **Ia**, **(1–5)a** and **(10–23)a** were resynthesized according to the described procedures [19].

### General procedure for the preparation of thiazoles 7, 8b

To a solution of compound **Ib** (0.7 g, 2.5 mmol) in acetone (10 mL), the corresponding haloketone (3.1 mmol) was added, and the mixture was refluxed for 2 h. Upon completion of the reaction (TLC), the mixture was cooled down, and the formed aminothiazolium bromide was filtered off, washed with acetone, dried and transferred into the base by refluxing in 2.5% aqueous sodium acetate (0.5 g/20 mL), cooling and filtering off the formed solid to give compound **7b** as a blueish solid, m. p. 168−169°C and compound **8b** as white solid, m. p. 188–189°C.

#### 3-((4-(4-Fluorophenyl)thiazol-2-yl)(naphthalen-1-yl)amino)propanoic acid (7b)

Yield 0.77 g (79%) IR (KBr) ν_max_ (cm^-1^): 3058 (OH), 1693 (CO), 1594 (C = N); ^1^H NMR (400 MHz, DMSO-*d*_*6*_): δ 2.61–2.85 (m, 2H, CH_2_CO), 3.74–4.69 (m, 2H, NCH_2_), 7.08 (s, 1H, SCH), 7.16–8.15 (m, 11H, H_Ar_), 12.19 (s, 1H, COOH); ^13^C NMR (101 MHz, DMSO-*d*_*6*_): δ 32.61 (CH_2_CO), 48.84 (NCH_2_), 103.01, 122.36, 126.50, 126.78, 127.29, 127.40, 127.68, 127.80, 128.80, 128.89, 129.14, 129.75, 130.24, 134.84, 139.38, 140.28, 149.04 (C_Ar_, S-CH = C), 170.07 (C = N), 172,65 (COOH). Calcd. for C_22_H_17_FN_2_O_2_S, %: C 67.33; H 4.37; N 7.14. Found, %: C 67.10; H 4.38; N 7.06.

#### 3-((4-(4-Chlorophenyl)thiazol-2-yl)(naphthalen-1-yl)amino)propanoic acid (8b)

Yield 0.92 g (90%) IR (KBr) ν_max_ (cm^-1^): 3051 (OH), 1700 (CO), 1531 (C = N); ^1^H NMR (400 MHz, DMSO-*d*_*6*_): δ 2.75 (t, 2H, *J* = 7.4 Hz, CH_2_CO), 3.93–4.92 (m, 2H, NCH_2_), 7.17 (s, 1H, SCH), 7.33–8.18 (m, 11H, H_Ar_), 12.26 (s, 1H, COOH); ^13^C NMR (101 MHz, DMSO-*d*_*6*_): δ 32.60 (CH_2_CO), 48.78 (NCH_2_), 104.02, 122.35, 126.49, 126.78, 127.29, 127.42, 128.61, 128.79, 129.12, 129.76, 131.93, 133.50, 134.82, 140.27, 149.02 (C_Ar_, S-CH = C), 170.08 (C = N), 172.64 (COOH). Calcd. for C_22_H_17_ClN_2_O_2_S, %: C 64.62; H 4.19; N 6.85. Found, %: C 64.44; H 4.01; N 6.87.

#### General procedure for the preparation of bis(thiazol-5-yl)phenylmethanes (24–31)b

To a mixture of the corresponding compound **(6–9)b** (3 mmol), the appropriate aromatic aldehyde (1.5 mmol) (molar ratio 2:1) and acetone (40 mL), the concentrated hydrochloric acid (0.5 mL) was added dropwise. The mixture was heated at reflux for 18 h and cooled down. The formed crystalline product was filtered off, washed with plenty of acetone and boiled in 4% aqueous sodium acetate for 5 min. The obtained appropriate product **(24–31)b** was filtered off, washed with water and dried.

**3,3’-((((4-Fluorophenyl)methylene)bis(4-(4-chlorophenyl)thiazole-5,2-diyl))bis(naphthalen-1-ylazanediyl))dipropionic acid (24b)** a greenish solid, yield 1.43 g (52%), mp 178–179°C; IR (KBr) ν_max_ (cm^-1^): 3340 (2x OH), 1708 (2x C = O), 1520 (2x C = N); ^1^H NMR (400 MHz, DMSO-*d*_*6*_): δ 2.37 (s, 4H, CH_2_CO), 3.51–4.73 (m, 4H, NCH_2_), 5.64 (s, 1H, CH), 6.81–8.46 (m, 26H, H_ar_), 12.30 (br. s, 2H, 2x COOH); ^13^C NMR (101 MHz, DMSO-*d*_*6*_): δ 34.02 (CH_2_CO), 40.54 (CCHC), 49.60 (NCH_2_), 115.43, 115.65, 122.25, 126.31, 126.64, 126.93, 127.07, 128.24, 128.59, 128.81, 129.21, 129.30, 129.35, 129.45, 129.62, 132.39, 133.45, 134.61, 139.17, 140.03, 159.72, 162.26, 167.78, 173.98 (C_ar_, C = N, C = O); HRMS m/z calculated for C_51_H_37_Cl_2_FN_4_O_4_S_2_ [M+H]+: 923.1617, found: 923.1698.

**3,3’-((((4-Chlorophenyl)methylene)bis(4-phenylthiazole-5,2-diyl))bis(naphthalen-1-ylazanediyl))dipropionic acid (25b)** a greenish solid, yield 1.15 g (44%), mp 191–192°C; IR (KBr) ν_max_ (cm^-1^): 3418 (2x OH), 1710 (2x C = O), 1520 (2x C = N); ^1^H NMR (400 MHz, DMSO-*d*_*6*_): δ 2.47 (m, 4H, CH_2_CO overlaps with the solvent), 3.61–4.62 (m, 4H, NCH_2_), 5.70 (s, 1H, CH), 6.83–8.17 (m, 28H, H_ar_), 12.05 (s, 2H, 2x COOH); ^13^C NMR (101 MHz, DMSO-*d*_*6*_): δ 33.77 (CH_2_CO), 40.75 (CCHC), 49.24 (NCH_2_), 122.25, 126.33, 126.64, 127.01, 127.70, 127.94, 128.23, 128.67, 128.81, 129.04, 129.47, 131,61, 134.60, 138.75, 140.07, 142.41, 147.24, 167.63, 173.63 (C_ar_, C = N, C = O); HRMS m/z calculated for C_51_H_39_ClN_4_O_4_S_2_ [M+H]+: 871.2101, found: 871.2195.

**3,3’-((((4-Bromophenyl)methylene)bis(4-phenylthiazole-5,2-diyl))bis(naphthalen-1-ylazanediyl))dipropionic acid (26b)** a greenish solid, yield 1.31 g (48%), mp 201–202°C; IR (KBr) ν_max_ (cm^-1^): 3417 (2x OH), 1712 (2x C = O), 1522 (2x C = N); ^1^H NMR (400 MHz, DMSO-*d*_*6*_): δ 2.46 (m, 4H, CH_2_CO overlaps with the solvent), 3.53–5.16 (m, 4H, NCH_2_), 5.68 (s, 1H, CH), 6.60–8.24 (m, 28H, H_ar_), 12.14 (s, 2H, 2x COOH); ^13^C NMR (101 MHz, DMSO-d_6_): δ 33.91 (CH_2_CO), 40.85 (CCHC), 49.34 (NCH_2_), 120.23, 122.28, 126.33, 126.66, 126.96, 127.03, 127.15, 127.72, 127.96, 128.26, 128.60, 128.85, 129.41, 131.59, 134.62, 140.09, 142.85, 167.64, 173.73 (C_ar_, C = N, C = O); HRMS m/z calculated for C_51_H_39_BrN_4_O_4_S_2_ [M+H]+: 915.1596, found: 915.1678.

**3,3’-((((4-Nitrophenyl)methylene)bis(4-phenylthiazole-5,2-diyl))bis(naphthalen-1-ylazanediyl))dipropionic acid (27b)** a greenish solid, yield 1.11 g (42%), mp 211–212°C; IR (KBr) ν_max_ (cm^-1^): 3420 (2x OH), 1711 (2x C = O), 1520 (2x C = N); ^1^H NMR (400 MHz, DMSO-*d*_*6*_): δ 2.59 (s, 4H, CH_2_CO), 3.67–4.60 (m, 4H, NCH_2_), 5.83 (s, 1H, CH), 6.88–8.27 (m, 28H, H_ar_), 12.15 (br. s, 2H, 2x COOH); ^13^C NMR (101 MHz, DMSO-d_6_): δ 32.93 (CH_2_CO), 41.29 (CCHC), 48.74 (NCH_2_), 122.22, 124.01, 126.36, 126.73, 127.90, 127.99, 128.34, 128.58, 128.63, 129.01, 134.37, 134.64, 139.93, 140.08, 146.36, 150.49, 167.90, 172.81 (C_ar_, C = N, C = O); HRMS m/z calculated for C_51_H_39_N_5_O_6_S_2_ [M+H]+: 882.2342, found: 882.2433.

**3,3’-((((4-Nitrophenyl)methylene)bis(4-(4-fluorophenyl)thiazole-5,2-diyl))bis(naphthalen-1-ylazanediyl))dipropionic acid (28b)** a greenish solid, yield 1.51 g (55%), mp 203–204°C; IR (KBr) ν_max_ (cm^-1^): 3406 (2x OH), 1712 (2x C = O), 1521 (2x C = N); ^1^H NMR (400 MHz, DMSO-*d*_*6*_): δ 2.53 (s, 4H, CH_2_CO overlaps with the solvent), 3.63–4.59 (m, 4H, NCH_2_), 5.73 (s, 1H, CH), 6.76–8.29 (m, 26H, H_ar_), 12.09 (s, 2H, 2x COOH); ^13^C NMR (101 MHz, DMSO-*d*_*6*_): δ 33.18 (CH_2_CO), 41.22 (CCHC), 48.93 (NCH_2_), 115.11, 115.32, 122.22, 124.02, 126.32, 126.73, 127.04, 128.67, 129.03, 130.02, 130.10, 130.90, 134.64, 139.98, 146.42, 150.16, 160.40, 162.83, 172.99 (C_ar_, C = N, C = O); HRMS m/z calculated for C_51_H_37_F_2_N_5_O_6_S_2_ [M+H]+: 918.2153, found: 918.2233.

**3,3’-((((4-Nitrophenyl)methylene)bis(4-(4-bromophenyl)thiazole-5,2-diyl))bis(naphthalen-1-ylazanediyl))dipropionic acid (29b)** a greenish solid, yield 1.67 g (54%), mp 241–242°C; IR (KBr) ν_max_ (cm^-1^): 3419 (2x OH), 1708 (2x C = O), 1520 (2x C = N); ^1^H NMR (400 MHz, DMSO-d_6_): δ 2.57 (s, 4H, CH_2_CO), 3.65–4.74 (m, 4H, NCH_2_), 5.77 (s, 1H, CH), 6.81–8.40 (m, 26H, H_ar_), 12.27 (br. s., 2H, 2x COOH); ^13^C NMR (101 MHz, DMSO-*d*_*6*_): δ 32.88 (CH_2_CO), 41.14 (CCHC), 48.74 (NCH_2_), 121.26, 122.17, 123.99, 126.75, 128.69, 129.08, 129.96, 131.27, 133.50, 134.64, 139.80, 146.44, 149.86, 167.92, 172.85 (C_ar_, C = N, C = O); HRMS m/z calculated for C_51_H_37_Br_2_N_5_O_6_S_2_ [M+H]+: 1038.0552, found: 1038.0623.

**3,3’-((((4-(Dimethylamino)phenyl)methylene)bis(4-phenylthiazole-5,2-diyl))bis(naphthalen-1-ylazanediyl))dipropionic acid (30b)** a greenish solid, yield 1.31 g (50%), mp 181–182°C; IR (KBr) ν_max_ (cm^-1^): 3425 (2x OH), 1713 (2x C = O), 1520 (2x C = N); ^1^H NMR (400 MHz, DMSO-*d*_*6*_): δ 2.60 (s, 4H, CH_2_CO), 2.74 (s, 6H, 2x CH_3_), 3.71–4.67 (m, 4H, NCH_2_), 5.60 (s, 1H, CH), 6.10–8.54 (m, 28H, H_ar_), 12.23 (br. s., 2H, 2x COOH); ^13^C NMR (101 MHz, DMSO-*d*_*6*_): δ 32.93 (CH_2_CO), 35.26 (CH_3_), 40.19 (CCHC), 48.61 (NCH_2_), 112.05, 122.30, 126.36, 126.66, 127.00, 127.50, 127.74, 127.92, 128.15, 128.61, 128.86, 130.80, 134.63, 134.84, 140.16, 148.94, 167.28, 172.80 (C_ar_, C = N, C = O); HRMS m/z calculated for C_53_H_45_N_5_O_4_S_2_ [M+H]+: 880.2913, found: 880.3006.

**3,3’-((((4-(Dimethylamino)phenyl)methylene)bis(4-(4-fluorophenyl)thiazole-5,2-diyl))bis(naphthalen-1-ylazanediyl))dipropionic acid (31b)** a greenish solid, yield 1.21 g (44%), mp 211–212°C; IR (KBr) ν_max_ (cm^-1^): 3430 (2x OH), 1713 (2x C = O), 1520 (2x C = N); ^1^H NMR (400 MHz, DMSO-*d*_*6*_): δ 2.53–2.65 (m, 4H, CH_2_CO), 2.75 (s, 6H, 2x CH_3_), 3.60–4.56 (m, 4H, NCH_2_), 5.48 (s, 1H, CH), 6.20–8.37 (m, 26H, H_ar_), 12.58 (br. s., 2H, 2x COOH); ^13^C NMR (101 MHz, DMSO-*d*_*6*_): δ 32.90 (CH_2_CO), 35.76 (CH_3_), 40.25 (CCHC), 48.63 (NCH_2_), 112.09, 114.94, 115.14, 122.29, 126.37, 126.67, 126.71, 127.78, 128.64, 128.93, 129.87, 129.93, 131.29, 131.66, 134.65, 140.09, 142.35, 149.01, 151.67, 172.75 (C_ar_, C = N, C = O); HRMS m/z calculated for C_53_H_43_F_2_N_5_O_4_S_2_ [M+H]+: 916.2725, found: 916.2797.

### Bacterial strains and culture conditions

The multidrug-resistant *S*. *aureus* strain TCH 1516 [USA 300-HOU-MR] was obtained from the American Type Culture Collection (ATCC). Vancomycin-intermediate *S*. *aureus*, *Clostridiodes difficile* (*C*. *difficile*), *Candida auris* (*C*. *auris*) strains were acquired from the Centers for Disease Control and Prevention (CDC) ARisolate bank. Prior to the initiation of this study, all microbial strains were stored in commercial cryopreservation systems at a temperature of -80°C. The strains were cultivated on Columbia Sheep Blood agar (Becton Dickinson, United States), Anaerobe agar for *C*. *difficile* (Becton Dickinson, United States) or Potato-Dextrose agar (PDA) for *C*. *auris* (Becton Dickinson, United States).

### Minimal inhibitory concentration determination

The antimicrobial activity of bis(thiazol-5-yl)phenylmethane derivatives was assessed using the broth microdilution method, following the guidelines outlined by the Clinical Laboratory Standards Institute (CLSI), with modification [20]. In brief, the compounds were dissolved in dimethylsulfoxide (DMSO) to attain a final concentration of 25 mg/mL. The control antibiotics and antifungals were obtained from MCE (MedChemExpress, United States) and dissolved in DMSO. Dilution series were prepared in deep 96-well microplates to achieve a two-fold concentration range of 0.25, 0.5, 1, 2, 4, 8, and 16 *μ*g/mL, utilizing Cation-Adjusted Mueller-Hinton Broth (CAMHB) as the growth medium. For *C*. *difficile* experiments, the dilutions were performed in CAMBH supplemented with 10% of leaked horse blood and 100 *μ*g/mL of L-cysteine. For *C*. *auris* screening, dilutions were performed in RMPI/MOPS media. The microplates containing the dilution series were then inoculated with fresh cultures of each tested organism to reach a final concentration of 5 × 10^4^ CFU (colony-forming units) of the test organism in media containing 1% DMSO and 1× drug concentration, with a volume of 200 *μ*l per well. Wells that were inoculated with media containing 1% DMSO served as positive controls. Subsequently, the microplates were incubated at 35 ± 1°C for 18 ± 2 hours. For *C*. *difficile* experiments, microplates were sealed in commercial GasPack anaerobic system and incubated at 35 ± 1°C for 24 hours. Following the incubation period, the plates were examined using a manual microplate viewer (Sensititre Manual Viewbox, United States). The minimal inhibitory concentration (MIC) was defined as the lowest concentration (*μ*g/mL) of the tested drug that completely inhibited the growth of the test organism. All experiments were conducted in duplicate with three technical replicates for each condition.

### Minimal bactericidal concentration determination

The minimal bactericidal concentration (MBC; *μ*g/mL) of bis(thiazol-5-yl)phenylmethane derivatives was determined as described before with slight modification [20]. Following the MIC determination using 96-well plates, an aliquot (10*μ*L) from MIC well and wells with increasing concentrations were taken and spotted on Columbia Sheep Blood agar. The plates were incubated at 35 ± 1°C for 24 hours and the MBC concentration was determined as the lowest concentration (*μ*g/mL) of test compounds that is fully suppressing the growth of test organism. All experiments were conducted in duplicate with three technical replicates for each condition.

### Biofilm integrity assay

Bacterial biofilm integrity assay was performed as described elsewhere with minor modifications [20]. Briefly, the test organism was cultured on Mueller-Hinton agar overnight to achieve well isolated colonies. One-two colonies were picked up and suspended in 5 mL of CAMBH and cultured overnight. The bacterial cultures were then diluted 1:100 with fresh CAMBH and subsequently cultured at 37°C, 200 rpm for 3–4 hours until OD600 reached 0.3. The culture was then dispensed in 96-well flat-bottomed microplates and was cultured overnight in static conditions to generate the mature biofilms. Next day, the media was aspirated and replaced with fresh CAMBH, containing 0.5% of DMSO and selected test compounds. The biofilms were further incubated for 24 hours at 37°C at static conditions. After cultivation, media was aspirated, the wells were washed 3 times with sterile PBS solution and fixed with 4% paraformaldehyde for 30 min, at room temperature. The paraformaldehyde was aspirated, fixed biofilms washed 3 times with PBS and stained with 0.5% of crystal violet for 1 hour. The plates were washed in running deionized water, dried at room temperature. The biofilm absorbed crystal violet was then solubilized with 10% aques acetic acid for 10 min, transferred to fresh flat bottom microplates and the absorbance was measured using a Multiscan Sky microplate spectrophotometer at OD.

### Molecular docking

#### Ligand preparation

The 3D structure of each compound (**23a** and **28b**) was built using Gaussview and was geometrically optimized using Avogadro [21]. These structures were visually checked to correct some structural errors.

#### Protein preparation

Twelve Mur family proteins in *Staphylococcus aureus* were selected for this study. The crystal structure of three selected proteins were retrieved from the Protein Data [22], including MurB (PDB 1HSK), MurE (PDB 4C12) and MurT (PDB 6GS2), where nine models were retrieved from AlphaFold Protein Structure Database, including MurA1, MurA2, MurC, MurD, MurF, MurG, MurP, MurQ and MurZ (with accession number Q6G7L0, Q5HE76, Q2FXJ0, P0A090, Q2FWH4, Q5HG02, W8U768, Q7A1Y2 and A0A2S6DFC3, respectively).

#### Docking of ligand-protein interaction

We resorted to virtual screening using Autodock Vina, a target-specific scoring method useful for virtual screening [21–23]. The derivatives **23a** and **28b** were docked into a set of proteins to identify potential biological targets. Both ligands and proteins were prepared using AutoDock Tools version 1.5.7 (ADT) according to the AutoDock Vina High Throughput screening standard method. Gasteiger partial charges were assigned to the atoms of ligands. The AutoTors option was used to define the rotatable bonds in the ligands. The visual inspection of the results was performed using the Molecular Graphics Laboratory (MGL) Tools package. We selected a grid volume enough to cover each receptor. Finally, graphical analysis was performed using Discovery Studio Visualizer, version 1.9.2 and Discovery Studio.

#### Cell lines and culture conditions

Human A549 pulmonary epithelial cells (ATCC CCl-185) were acquired from the ATCC. These cells were cultivated in DMEM/F12 medium enriched with GlutaMAX (ThermoFisher Scientific, Waltham, MA, USA), Penicillin-Streptomycin (PenStrep) (ThermoFisher Scientific, Waltham, MA, USA), and 10% heat-inactivated fetal bovine serum (FBS) (ThermoFisher Scientific, Waltham, MA, USA).

Human THP-1 monocytes (ATCC TIB-202), also obtained from ATCC, were maintained in complete RPMI medium (ThermoFisher Scientific, Waltham, MA, USA) supplemented with GlutaMAX (ThermoFisher Scientific, Waltham, MA, USA), 10% heat-inactivated FBS (Gibco, ThermoFisher Scientific, Waltham, MA, USA), and 50 *μ*M of beta-mercaptoethanol. Both cell lines were cultivated at 37°C under 5% CO_2_ atmospheric conditions. For the initiation of differentiation of THP-1 monocytes into macrophages, the cells were exposed to complete RPMI medium containing 200 ng/mL of Phorbol 12-myristate 13-acetate (PMA) (Sigma-Aldrich, Schnelldorf, Germany) for a duration of 48 hours. Subsequently, the cells underwent a resting period in PMA-free RPMI medium for an additional 48 hours prior to their utilization in experimental assays.

#### Cytotoxicity assay

The *in vitro* inhibitory effects of the compounds were assessed using the MTT assay, as described in previous studies [24, 25]. The A549 or THP-1 derived macrophages were seeded into 96-well plates at a concentration of 1 × 10^4^ cells per well. Following an overnight incubation at 37°C with 5% CO_2_, the cells were treated with the compounds at a concentration of 100 *μ*M, and this treatment was carried out in triplicate. After a 20-hour exposure period, the MTT reagent was introduced, and the cells were subsequently incubated for an additional 4 hours. To serve as a cytotoxicity control, a 10% solution of sodium dodecyl sulfate (SDS) was employed. The formazan product resulting from the MTT assay was extracted using anhydrous dimethyl sulfoxide (DMSO). The optical density of the samples was determined using a microplate reader, specifically at a wavelength of 570 nm. To ascertain the percentage of A549 cell viability, the following formula was applied: ([AE-AB]/[AC-AB]) × 100%, with AE representing the absorbance of the experimental samples, AC representing the absorbance of untreated samples, and AB representing the absorbance of blank controls. The obtained data were subjected to analysis using statistical software such as GraphPad Prism or QuickCalcs.

#### Rabbit erythrocyte hemolysis assay

Rabbit erythrocytes were suspended in pre-warmed phosphate buffered saline (PBS) containing 0.1% of DMSO. Then test compounds were added to reach 100 *μ*M concentration and then samples were incubated for 1 hour at 37°C. 10% SDS was used as a hemolysis control. Following the incubation, samples were centrifuged at 1000 × g for 10 min., and the released hemoglobin was measured spectrophotometrically at the optical density of 405 nm (OD 405 nm).

#### Statistical analysis

The results are expressed as mean ± standard deviation (SD). Statistical analyses were performed with Prism (GraphPad Software, San Diego, CA, USA), using Kruskal–Wallis test and two-way ANOVA. *P* < 0.05 was accepted as significant.

## Results

### Synthesis of compounds

The interest to the synthesis and investigation of bis(thiazoles) **(10–23)a** and **(24–31)b** follows from the fact that analogous compounds exhibited a promising antibacterial activity against *M*. *luteum* in our previously publication [19]. Therefore, in this study we have explored the synthetic versatility and structure-depended activity (SAR) of bis(thiazoles) against panel of multidrug-resistant Gram-positive and Gram-negative pathogens with emerging multidrug-resistance phenotypes.

**Scheme 1:**
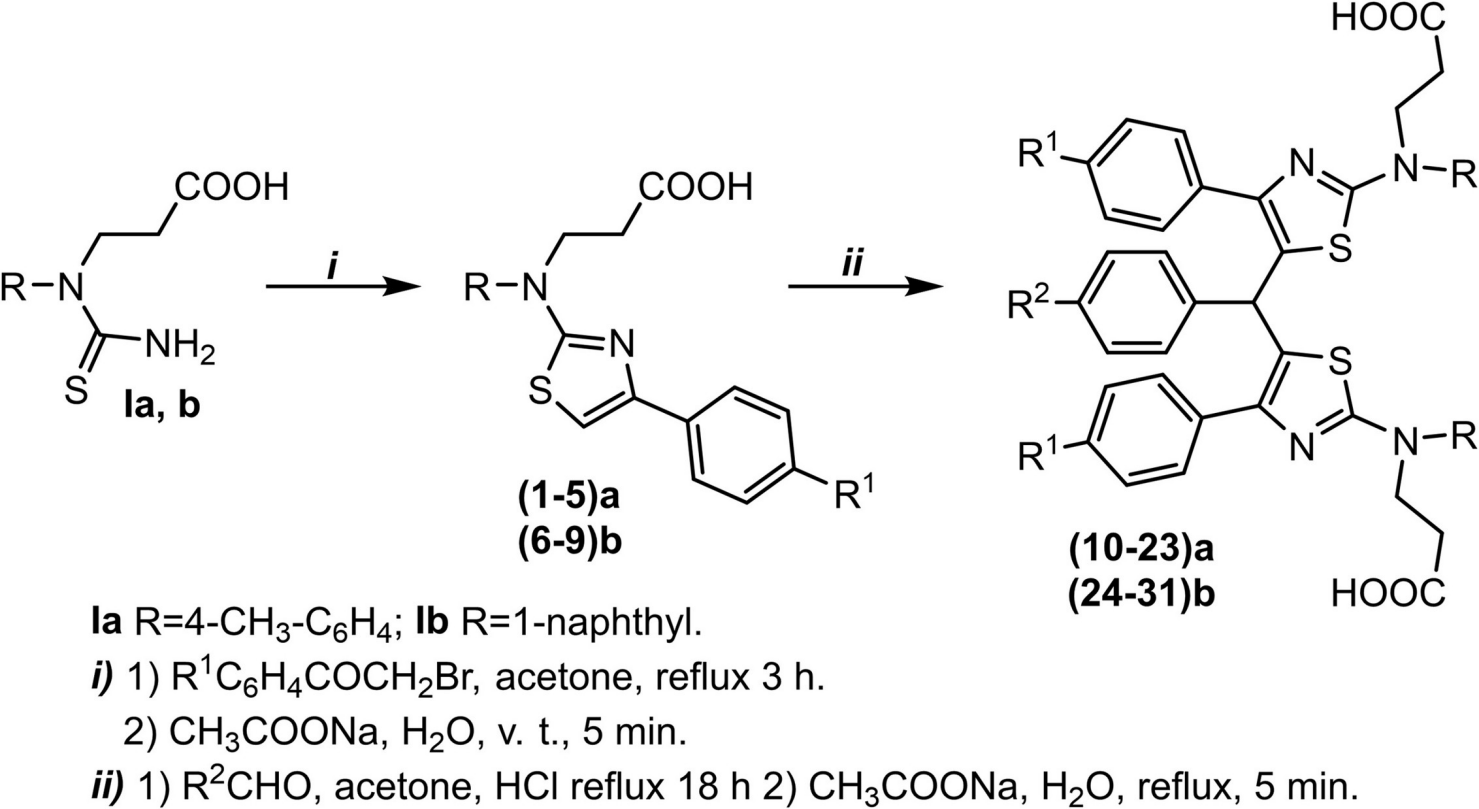
Synthesis of bis(thiazol-5-yl)p-tolyl detivatives (10–23)a and (24-31b).

The reactions of thiazole derivatives **(1–5)a** and **(6–9)b** with aromatic aldehydes in a 2:1 molar ratio and in the presence of a catalytic amount of concentrated hydrochloric acid afforded bis(thiazol-5-yl)phenylmethanes **(10–23)a, (24–31)b** (Table 1 and Scheme 1), which have already been crystallized in the reaction mixture. The obtained appropriate product was filtered off, washed with water, and dried. The products were elucidated based on their IR, NMR, and mass spectroscopy data. The analysis of the ^1^H NMR spectra of compounds **(24–31)b** revealed a singlet at approx. 5.74 ppm, ascribed to the newly formed C-CH-C fragment, which is clearly confirmed by the resonance line at approx. 40.8 ppm in the ^13^C NMR spectra. Both spectra displayed an increased abundance of the aromatic signals as well. The characteristic resonances lines at 160.99 ppm (*J* = 255.7 Hz, ^13^C NMR spectra) and at 115.54 (*J* = 21.4 Hz, ^13^C NMR spectra) show the splitting influence of fluorine atom in the compound **24b**.

**Table 1:** The effect of various substitutions incorporated in bis(thiazol-5-yl)p-tolyl derivatives (10–23)a and (24-31b) on the obtained synthesized yealds.

Compound	R	R^1^	R^2^	Yield
**1a**	**p-tolyl**	-H	**-**	89%
**2a**		-F		87%
**3a**		-Cl		99%
**4a**		-CN		85%
**5a**		-NO_2_		89%
**6b**	**naphtyl**	-H		86%
**7b**		-F		79%
**8b**		-Cl		90%
**9b**		-Br		54%
**10a**	**p-tolyl**	-H	-F	44%
**11a**		-Cl		26%
**12a**		-NO_2_		39%
**13a**		-CN	-Cl	41%
**14a**		-NO_2_		42%
**15a**		-NO_2_	-Br	38%
**16a**		-H	-NO_2_	47%
**17a**		-F		40%
**18a**		-Cl		43%
**19a**		-NO_2_		37%
**20a**		-H	-N(CH_3_)_2_	47%
**21a**		-F		35%
**22a**		-Cl		37%
**23a**		-NO_2_		41%
**24b**	**naphtyl**	-Cl	**-**F	45%
**25b**		-H	**-**Cl	48%
**26b**		-H	**-**Br	43%
**27b**		-H	-NO_2_	41%
**28b**		-F		45%
**29b**		-Br		39%
**30b**		-H	-N(CH_3_)_2_	36%
**31b**		-F		42%

### Enhanced antimicrobial activity against Gram-positive pathogens by bis(thiazol-5-yl)phenylmethane derivatives (10–23)a and (24–31)b

To comprehensively evaluate the antimicrobial properties of bis(thiazol-5-yl)phenylmethane derivatives **10–31**, we conducted antimicrobial activity determination assay using a panel of representative multidrug-resistant bacterial and fungal strains. The antimicrobial activity of these compounds was first evaluated at a fixed concentration of 100 *μ*M. We refined the CLSI broth dilution method and employed resazurin viability assays in combination with spectrophotometric analysis to thoroughly characterize the inhibitory potential of compounds **10–31** against the WHO priority bacterial and fungal strains.

**Fig 1:**
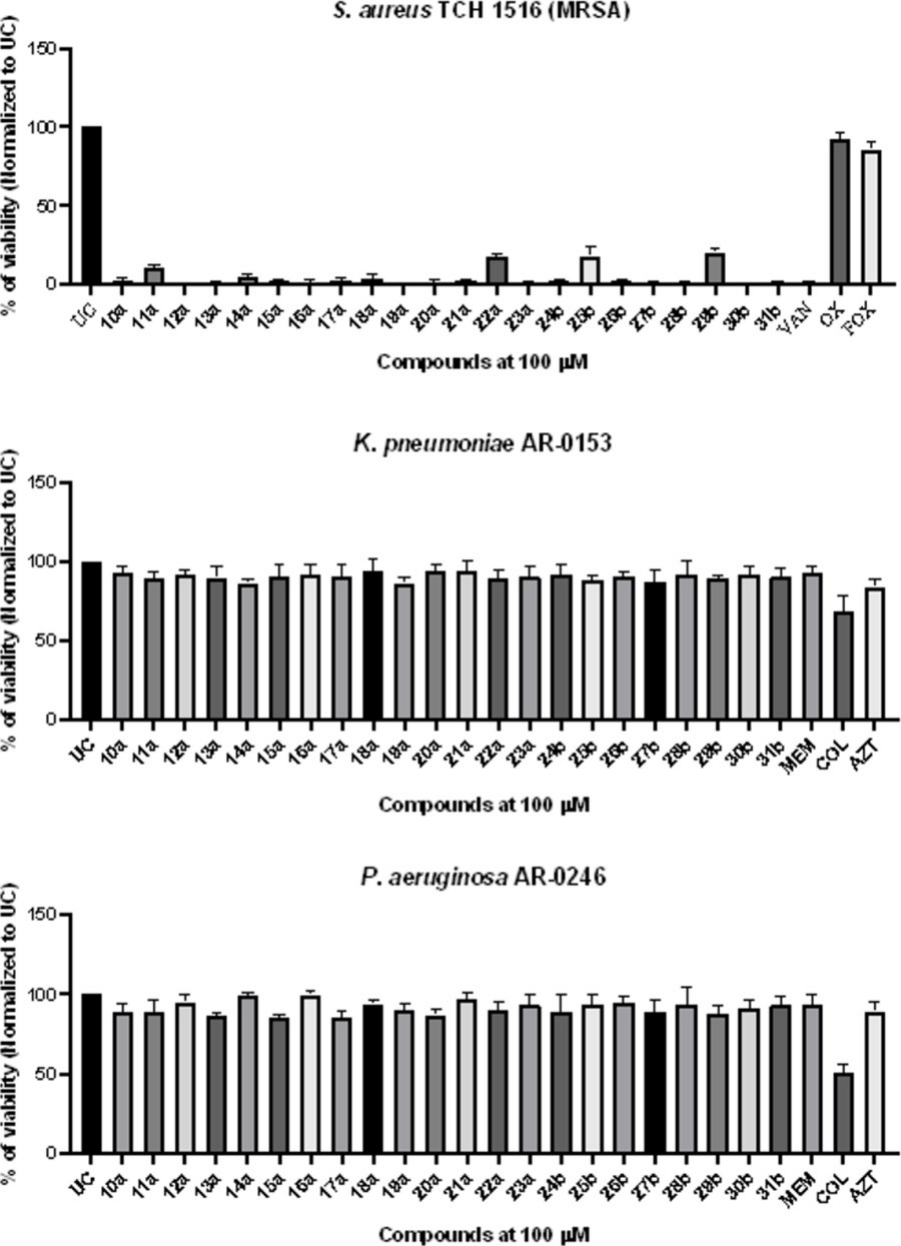
*In vitro* antimicrobial activity of bis(thiazol-5-yl)phenylmethane derivatives 10–31 and control antimicrobial compounds at a concentration of 100 *μ*M against selected multidrug-resistant bacterial strains. Bacterial strains were exposed to compounds and control antimicrobial drugs at a fixed concentration of 100 *μ*M for 18 hours. Subsequently, resazurin (25 *μ*M) was added, and the plates were further incubated for 3 hours. Following incubation, the optical density at 700 nm (OD_700 nm_) was measured, and the post-treatment viability percentage was normalized to the untreated control (UC). AZT-aztreonam, COL-colistin, FOX-cefoxitin, MEM-meropenem, OX-oxacillin, VAN-vancomycin. The data presented in the figure represents the mean ± standard deviation (SD) from three independent experimental replicates.

In our initial screening, we tested these compounds against a range of clinically significant pathogens, including the hypervirulent Gram-positive strain *S*. *aureus* TCH1516 (belonging to the MRSA USA300 clade), the Gram-negative New Delhi metallo-beta-lactamase 1 (NDM-1) producing *K*. *pneumoniae* AR-0153, and the non-fermenting NDM-1 producing *P*. *aeruginosa* AR-0246 (Fig 1). Additionally, we included the fluconazole-resistant *C*. *auris* AR-0386 of the African clade in our study (S1 Fig in S1 File).

Upon exposure to these strains, compounds **10–31** demonstrated favorable antimicrobial activity, with a particular selective activity against *S*. *aureus* TCH 1516 (Fig 1). Importantly, the antimicrobial activity was found to be structure-dependent, resulting in a reduction in the viability of *S*. *aureus* TCH 1516, ranging from 0.4% to 20.7% (*p*<0.05). Notably, compounds **11a** (R^1^-Cl, R^2^-F), **22a** (R^1^-Cl, R^2^-N(CH_3_)_2_), which feature a methylbenzene radical, as well as the naphtalene derivative **25b** (R^1^-H, R^2^-Cl), and **29b** (R^1^-Br, R^2^-NO_2_), exhibited reduced antimicrobial activity against the *S*. *aureus* TCH 1516 strain (Fig 1).

Intriguingly, compounds **10–31**, did not displayed antimicrobial activity against multidrug-resistant *K*. *pneumoniae* AR-0153 or *P*. *aeruginosa* AR-0246 (Fig 1). Moreover, no antifungal activity was observed when these compounds were tested against drug-resistant *C*. *auris* AR-0386 (S1 Fig in S1 File). These observations collectively suggest that bis(thiazol-5-yl)phenylmethane scafold exhibit promising antimicrobial activity primarily against the Gram-positive bacterium *S*. *aureus* strain TCH 1516, emphasizing their potential as selective antimicrobial candidate.

### Bis(thiazol-5-yl)phenylmethane derivatives 10–31 exhibit limited antimicrobial activity against drug-resistant *Enterococcus*, *Streptococcus*, and *Clostridioides difficile*

After observing antimicrobial activity directed against *S*. *aureus* TCH 1516 by compounds **10–31**, we sought to investigate whether the observed efficacy of bis(thiazol-5-yl)phenylmethane derivatives **10–31** is specific to *S*. *aureus* or if it extends to other Gram-positive bacterial pathogens. To address this question, we selected representative Gram-positive strains of clinical significance and determined their minimal inhibitory concentrations (MIC; *μ*g/mL) following the guidelines set by the CLSI (Table 2).

**Table 2:** *In vitro* minimal inhibitory concentration (MIC) values (μg/mL) of compounds 10–31 against drug-resistant Gram-positive bacterial strains.

Compound	*E*. *faecalis* AR-0781 (VRE)^a^	*E*. *faecalis* AR-0782 (VRE)^b^	*C*. *difficile*AR-1067^c^	*S*. *agalactiae* STS011^d^	*S*. *agalactiae* STS031^d^
**10a**	64	32	<64	64	64
**11a**	<64	<64	<64	64	64
**12a**	<64	<64	<64	64	64
**13a**	<64	<64	<64	32	64
**14a**	32	64	32	<64	<64
**15a**	32	32	64	<64	<64
**16a**	16	32	<64	<64	<64
**17a**	16	16	<64	<64	<64
**18a**	64	<64	<64	<64	<64
**19a**	64	<64	16	16	<64
**20a**	32	64	<64	64	64
**21a**	<64	<64	<64	64	64
**22a**	<64	<64	<64	64	64
**23a**	16	16	<64	64	64
**24b**	16	16	<64	64	64
**25b**	64	32	<64	<64	<64
**26b**	32	32	<64	<64	<64
**27b**	<64	<64	64	<64	<64
**28b**	<64	<64	64	<64	<64
**29b**	<64	<64	16	<64	64
**30b**	<64	<64	<64	<64	64
**31b**	<64	<64	<64	32	64
Doxycycline	16	>1	16	8	16
Clindamycin	8	8	32	1	1
Vancomycin	<64	64	2	2	1
Ceftriaxone	32	8	64	1	1

a. Vancomycin-resistant *Enterococcus faecalis* (VRE) harboring *tet(L)*, *tet(M)*, *VanA* resistance mechanisms.

b. Vancomycin-resistant *Enterococcus faecalis* (VRE) harboring *aac(6’)-Ie*, *ant(6)-Ia*, *aph(3’)-IIIa*, *catA8*, *qacZ*, *VanB* resistance mechanisms.

c. MLST type ST1, ribotype 027. Toxin A and toxin B producing *C*. *difficile* strain.

b. Strains harboring *tetM* or *tetO* tetracycline resistance genes, previously reported by Rodgus *et al*. [24].

We observed that bis(thiazol-5-yl)phenylmethane derivatives **10–31** exhibit moderate antimicrobial activity against vancomycin-resistant *Enterococcus* (VRE) strains, with MIC values ranging from 16 to more than 64 *μ*g/mL (Table 2). Weak antimicrobial activity was also noted when group B *Streptococcus agalactiae* (BGS) strains were subjected to MIC determination, with MIC values of 64 *μ*g/mL and higher (Table 2). Notably, among the tested compounds, **17a** (R^1^-F, R^2^-NO_2_), **23a** (R^1^-NO_2_, R^2^-N(CH_3_)_2_), and **24b** (R^1^-Cl, R^2^-F) displayed the highest activity against VRE, exhibiting an MIC value of 16 *μ*g/mL. However, these same compounds exhibited no or low activity against *S*. *agalactiae* STS011 and STS031 strains (MIC values of 64 *μ*g/mL or higher) or *C*. *difficile* (MIC > 64 *μ*g/mL) (Table 2).

On the other hand, only the nitrated compound **14a** (R^1^-NO_2_, R^2^-Cl) exhibited activity against *C*. *difficile*, with an MIC value of 32 *μ*g/mL. Conversely, replacing the chlorine at the R^2^ position with a bromine radical **15a** resulted in decreased antimicrobial activity, with an MIC of 64 *μ*g/mL (Table 2). Furthermore, nitrated compound **19a** demonstrated good antimicrobial activity against *C*. *dificile* (MIC 16 *μ*g/mL), *S*. *agalactiae* SAS011 (MIC 16 *μ*g/mL) but not *S*. *agalactiae* STS031 (MIC <64 *μ*g/mL) (Table 2).

Furthermore, naphthalene derivatives (**27–29)b** containing a nitro radical at the R^2^ position demonstrated antimicrobial activity against *C*. *difficile*. Compound **27b** (R^1^-H) exhibited weak activity, with an MIC of 64 *μ*g/mL, while the replacement of R^1^-H with fluorine (**28b**) had no effect. Intriguingly, the introduction of a bromine atom at the R^1^ position (**29b**) significantly increased the activity against *C*. *difficile*, resulting in an MIC of 16 *μ*g/mL. These findings underscore the importance of substituents at the R^1^ position for directing antimicrobial activity against anaerobic *C*. *difficile*.

### Bis(thiazol-5-yl)phenylmethane derivatives 10–31 exhibit promising activity against S*taphylococcus aureus* strains with emerging resistance mechanisms

After characterizing the antimicrobial activity of compounds **10–31** against a panel of Gram-positive clinical strains, including *Enterococcus* spp., *Streptococcus* spp., and *Clostridiodes* spp., we sought to determine whether the observed antimicrobial activity against *S*. *aureus* was influenced by pre-existing antimicrobial resistance phenotypes. To address this question comprehensively, we selected representative strains with varying resistance profiles, encompassing methicillin-susceptible (MSSA) wild-type, MRSA, and VRSA (Table 3).

**Table 3:** *In vitro* MIC values (μg/mL) of compounds 10–31 against *Staphylococcus aureus* with genetically defined resistance mechanisms.

Compound	*S*. *aureus* SA-1001 (MSSA)^a^	*S*. *aureus* 2717–2 (MSSA)^a^	*S*. *aureus* TCH 1516 (MRSA)	*S*. *aureus* ME-311 *(*MRSA)^b^	*S*. *aureus* VA13 (VRSA)^c^
**10a**	4	4	8	8	16
**11a**	16	16	32	16	16
**12a**	4	4	4	4	4
**13a**	8	8	8	8	8
**14a**	4	8	16	16	16
**15a**	8	16	32	16	16
**16a**	8	8	4	4	4
**17a**	4	4	4	4	4
**18a**	8	16	8	8	8
**19a**	4	4	4	4	8
**20a**	4	8	16	4	4
**21a**	8	8	8	8	4
**22a**	64	64	64	64	64
**23a**	4	4	4	4	4
**24b**	8	8	4	4	4
**25b**	16	32	16	16	4
**26b**	16	16	8	8	8
**27b**	8	4	4	4	4
**28b**	2	2	2	2	2
**29b**	32	64	16	16	64
**30b**	4	4	2	2	2
**31b**	8	8	2	2	2
Clindamycin	1	>1	4	8	4
Doxycycline	1	2	8	16	32
Vancomycin	>1	>1	2	2	16
Ceftriaxone	>1	1	8	4	4

a. Pan-susceptible strains harboring *blaZ*. Previously described by Kavaliauskas *et al*. [20].

b. Methicillin-resistant *S*. *aureus* (MRSA) strain harboring *SCmecA* and *ermA*. Previously described by Kavaliauskas *et al*. [20].

c. Vancomycin-resistant *S*. *aureus* (VRSA) strain harboring *vanA*, *aac(6′)-aph(2″)*, *tetK*. Previously described by Kavaliauskas *et al*. [20].

Our investigations revealed that compounds **10–31** exhibited a structure-dependent antimicrobial activity, which was only slightly affected by the pre-existing resistance profiles of the *S*. *aureus* strains. Both MSSA, MRSA, and VRSA strains displayed similar susceptibility profiles, with MIC values ranging from 2 to 64 *μ*g/mL. Notably, compounds containing a p-tolyl core with a fluorine atom substitution at the R^2^ position demonstrated structure-dependent activity when the R^1^ position was modified with halogen or a nitro group (Table 3).

Specifically, compound **10a** (R^1^-H) exhibited notable antimicrobial activity against MSSA (MIC 4 *μ*g/mL), MRSA (MIC 8 *μ*g/mL), and VRSA (MIC 16 *μ*g/mL). The introduction of a chlorine atom at the R^1^ position in **11a** resulted in altered antimicrobial activity against MSSA (MIC 16 *μ*g/mL) and MRSA strains (MIC 16–32 *μ*g/mL), although it had no effect on VRSA susceptibility (MIC 16 *μ*g/mL). Interestingly, replacing the chlorine atom with a nitro group (**12a**) led to an enhancement in antimicrobial activity against all strains, with a consistent MIC of 4 *μ*g/mL observed across all strains (Table 3).

The introduction of a nitrile group at the R^1^ position and a chlorine atom at the R^2^ position resulted in equal antimicrobial activity against all resistance types, with a MIC of 8 *μ*g/mL observed for compound **13a**. However, replacing the nitrile at the R^1^ position with a nitro substituent **14a** decreased antimicrobial activity against MRSA and VRSA strains, leading to an MIC of 16 *μ*g/mL although increased activity was observed against pan-susceptible *S*. *aureus* SA-1001 (MSSA). Subsequently, substituting chlorine with bromine at the R^2^ position in the nitro compound **14a** resulted in compound **15a**. Compound **15a** demonstrated favorable antimicrobial activity against *S*. *aureus* strains with emerging resistance phenotypes, including VRSA. Furthermore, compound **15a** which exhibited similar MIC values across all *S*. *aureus* MSSA (8–16 *μ*g/mL) and MRSA (16–32 *μ*g/mL) strains, except for *S*. *aureus* TCH 1516 MRSA strain where MIC was observed as 32 *μ*g/mL (Table 3).

The importance of the R^2^ position in the antimicrobial activity of bis(thiazol-5-yl)phenylmethanes was further elucidated by introducing an acidic electron-withdrawing nitro group at the R^2^ position and varying substituents at the R^1^ position. Compound **16a** with R^1^-H displayed enhanced antimicrobial activity against MRSA and VRSA strains, with an MIC of 4 *μ*g/mL. Substituting hydrogen with halogens such as fluorine **17a** increases the antimicrobial activity against MSSA, MRSA and VRSA resistance phenotypes, while introduction of chlorine **18a** at the R^1^ position weakens the antimicrobial activity against all tested *S*. *aureus* resistance phenotypes. Intriguingly, adding a nitro group to the R^1^ position resulted in compound **19a**, which contained three nitro groups and displayed similar MIC profiles against MSSA and MRSA strains (MIC 4 *μ*g/mL), while the VRSA strain exhibited a slightly elevated MIC (8 *μ*g/mL) (Table 3).

Replacing the acidic nitro group at the R^2^ position with a basic dimethylamine group resulted in compounds with similar activity. Dimethylamine derivatives **20a** and **21a**, containing H or F substituents at the R^1^ position, displayed comparable activity against all strains. The introduction of Cl at the R^1^ position **22a** resulted in decreased activity against all strains (MIC 64 *μ*g/mL), while Cl substitution with a nitro group **23a** restored activity against all resistance phenotypes (Table 3).

Bis(thiazol-5-yl)phenylmethanes containing a naphthalene core and substitutions at R^1^ and R^2^ were also examined. Compound **24b**, containing R^1^-Cl and R^2^-F, exhibited selectively lower MIC against MRSA and VRSA strains (MIC 4 *μ*g/mL) compared to MSSA strains (MIC 8 *μ*g/mL). Replacing the fluorine atom at R^2^ with chlorine or bromine (**25b** and **26b**) while maintaining hydrogen at R^1^ decreased antimicrobial activity, while the addition of a nitro substituent at R^2^ (**27-29b**) modulated antimicrobial activity based on the R^1^ substituent. Compound **27b** (R^1^-H; R^2^-NO_2_) displayed good activity against MRSA and VRSA strains (MIC 4 *μ*g/mL) with the exception of *S*. *aureus* SA-1001 (MIC 8 *μ*g/mL), while incorporating fluorine at the R^1^ position in compound **28b** resulted in enhanced antimicrobial activity against all strains, with an MIC of 2 *μ*g/mL. The presence of a fluorine atom at the R^1^ position appeared to be crucial for potent activity, as replacing fluorine with bromine (**29b**) resulted in considerable decreased antimicrobial activity (Table 2).

Finally, the addition of a dimethylamino substituent at R^2^ (**30b,** R^1^-H) led to stronger activity against MRSA and VRSA phenotypes (MIC 2 *μ*g/mL) compared to MSSA strains (MIC 4 *μ*g/mL), while the addition of fluorine at the R^1^ position (**31b**) further reduced activity against MSSA strains (MIC 8 *μ*g/mL). Based on the results generated from screening, two compounds (**23a** and **28b**) were selected for further studies. The compounds were selected based on their fixed MIC values across various strains with defined resistance profiles. Furthermore, compounds **23a** and **28b** were selected for further characterization based on their bactericidal activity, as observed in measuring minimal bactericidal concentration (MBC), that was significantly close to MIC values for compounds **23a** and **28b** (S1 Table in S1 File).

Following the identification of the most potent bactericidal candidates, we examined the effect of serum addition to the antimicrobial activity of compounds **23a** and **28b**. We conducted *in vitro* MIC determination in the presence of 50% fetal bovine serum (FBS), to elucidate the serum protein binding properties, crucial for further hit to lead optimization. Compound **23a** exhibited a capacity to bind serum proteins, as its MIC was significantly increased (*p* < 0.005) (MIC 64 *μ*g/mL) in all tested strains compared to the serum-free control (MIC 4 *μ*g/mL) (Table 4). In contrast, the antimicrobial activity of naphthalene derivative **28b** increased the MIC for *S*. *aureus* SA-1001 (MSSA) and *S*. *aureus* VR13 (VRSA) strains (MIC 4 *μ*g/mL) (Table 4).

**Table 4:** The effect of serum addition of the MIC values (μg/mL) for compounds 23a and 28b.

Bacteria	Phenotype	Compound 23a	Compound 23a+50% FBS	Compound 28b	Compound 28b+50% FBS
***S*. *aureus* SA-1001**	MSSA	4	64	2	4
***S*. *aureus* TCH 1516**	MRSA	4	64	2	2
***S*. *aureus* VR13**	VRSA	4	<64	2	4

Abbreviations: FBS–fetal bovine serum.

These data highlight the robust antimicrobial activity of bis(thiazol-5-yl)phenylmethanes against *S*. *aureus*, even in the presence of genetically defined and challenging resistance mechanisms. Additionally, our structure-activity relationship studies revealed that the presence of a naphthalene substitution with fluorine at the R^1^ position and a nitro group at the R^2^ position is pivotal for eliciting potent antimicrobial activity against *S*. *aureus*, while maintaining minimal serum protein binding.

### Bis(thiazol-5-yl)phenylmethane derivatives exhibit the ability to disperse mature *S*. *aureus* biofilms *in vitro*

**Fig 2:**
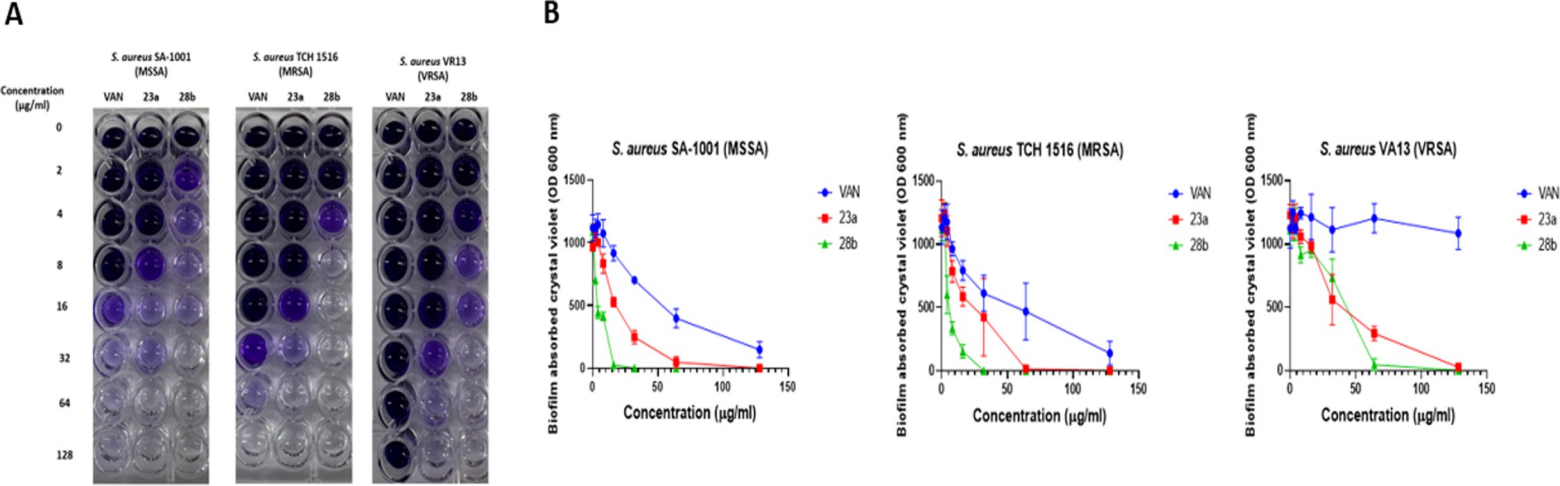
Bis(thiazol-5-yl)phenylmethane derivatives 23a and 28b exhibit a anti-biofilm activity against *S*. *aureus* with genetically defined resistance mechanisms. Bacterial biofilms were grown for 24 hours in static conditions and then biofilms were exposed with compounds **23a, 28b** or vancomycin (VAN) in fresh media for 18 hours. Biofilm mass was measured by using crystal violet assay. Panel A shows representative images of crystal violet assay. Panel B demonstrates the spectrophotometric measurements of compound-exposed biofilms. The data presented in the figure represents the mean ± standard deviation (SD) from three independent experimental replicates.

After identifying the two most potent compounds, **23a** and **28b**, we proceeded to investigate whether bis(thiazol-5-yl)phenylmethanes **23a** and **28b** could impact *S*. *aureus* biofilms, which are known to be crucial for *S*. *aureus* virulence and pathogenesis. We compared the anti-biofilm activity of these compounds with that of vancomycin (VAN), a high molecular mass approved antibiotic commonly employed for treating infections caused by Gram-positive pathogens (Fig 2A and 2B).

The compounds **23a** and **28b** exhibited a concentration-dependent anti-biofilm activity that was evident across all tested strains. Importantly, both compounds displayed significant anti-biofilm activity against all tested *S*. *aureus* resistance phenotypes. Specifically, bis(thiazol-5-yl)phenylmethane **23a** demonstrated robust antibiofilm activity against MSSA, MRSA, and vancomycin-resistant *S*. *aureus* (VRSA) strains. Compound **28b**, on the other hand, exhibited remarkably potent antibiofilm activity against all tested strains, surpassing the efficacy of vancomycin (Fig 2A and 2B).

These findings strongly indicate that compounds **23a** and **28b** possess the capability to exert promising antibiofilm activity against mature *S*. *aureus* biofilms *in vitro*. The notably enhanced anti-biofilm activity of compound **28b** implies the crucial role played by the fluorine and nitro substituents in conferring this biofilm-disrupting capability.

### Bis(thiazol-5-yl)phenylmethane derivatives 23a and 28b interacts with *S*. *aureus* MurC protein

After performing *in silico* docking analysis using compounds **23a** and **28b** and twelve *S*. *aureus* Mur ligases, the compounds **23a** and **28b** were calculated to bind more strongly to MurC with ΔG_bin_ values of -9.6 and -11.4 respectively, as shown in Table 5.

**Table 5:** Predicted binding free energy values (ΔG_*bin*_, kcal/mol) for the docking of compounds with Mur family proteins in *S*. *aureus*.

Gene name	Compound Protein name	23a	28b
MurA1	Acetylglucosamine 1-carboxyvinyltransferase 1	-7.4	-8.6
MurA2	UDP-N-acetylglucosamine 1-carboxyvinyltransferase 2	-7.7	-9.1
MurB	UDP-N-acetylenolpyruvoylglucosamine reductase	-9.2	-10.1
**MurC**	**UDP-N-acetylmuramate-L-alanine ligase**	**-9.6**	**-11.4**
MurD	UDP-N-acetylmuramoylalanine-D-glutamate ligase	-8.6	-9.8
MurE	UDP-N-acetylmuramoyl-L-alanyl-D-glutamate-L-lysine ligase	-8.6	-8.9
MurF	UDP-N-acetylmuramoyl-tripeptide—D-alanyl-D-alanine ligase	-8.4	-8.7
MurG	UDP-N-acetylglucosamine-N-acetylmuramyl-(pentapeptide) pyrophosphoryl-undecaprenol N-acetylglucosamine transferase	-9.4	-9.8
MurP	N-acetylmuramic acid phosphotransfer permease	-8.5	-8.2
MurQ	N-acetylmuramic acid 6-phosphate etherase	-8.2	-8.2
MurT	Lipid II isoglutaminyl synthase	-7.7	-8.3
MurZ	UDP-N-acetylglucosamine 1-carboxyvinyltransferase	-7.9	-8.5

Proteins with their respective (**AlphaFold**) entries: MurA1 (Q6G7L0), MurA2 (Q5HE76), MurC (Q2FXJ0), MurD (P0A090), MurF (Q2FWH4), MurG (Q5HG02), MurP (W8U768), MurQ (Q7A1Y2) and MurZ (A0A2S6DFC3).

Proteins with their respective (**PDB**) entries: MurB (1HSK), MurE (4C12) and MurT (6GS2).

**Fig 3:**
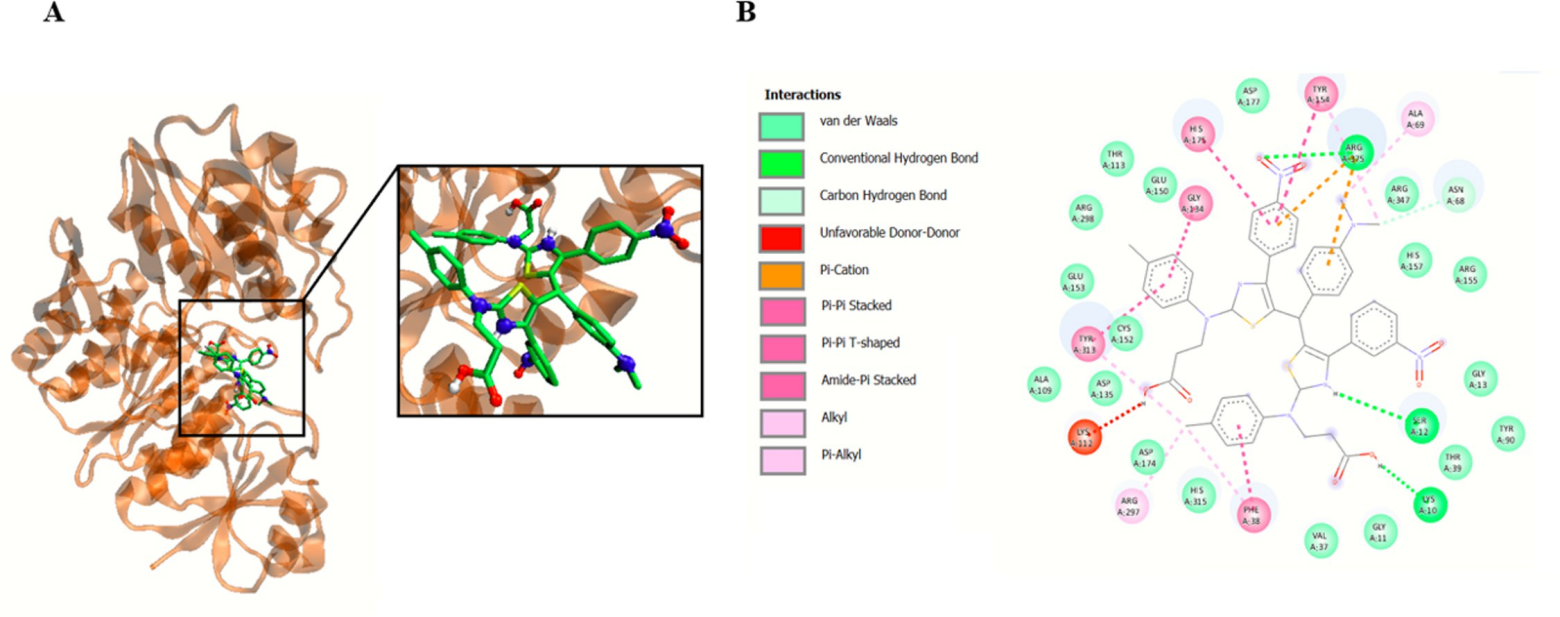
Compound 23a interacts with *S*. *aureus* MurC ligase. Panel A demonstrates the potential binding site of **23a** into MurC. Panel B shows plotted 2D maps of H-bonds and hydrophobic interactions of **23a** with MurC residues. Van der Waals, Pi–Cation, Pi–Pi stacked, Pi–Pi T-shaped, amide-Pi stacked, alkyl and Pi–alkyl are considered hydrophobic interactions.

After performing the initial calculations and identifying *S*. *aureus* MurC as a target for compounds **23a** and **28b**, we then explored potential binding sites and poses of the compounds **23a** and **28b** into MurC. We found that compound **23a** positions in MurC active center and interacts with MurC Lys 10, Ser12 and Arg375 amino acids (Fig 3A and 3B).

**Fig 4:**
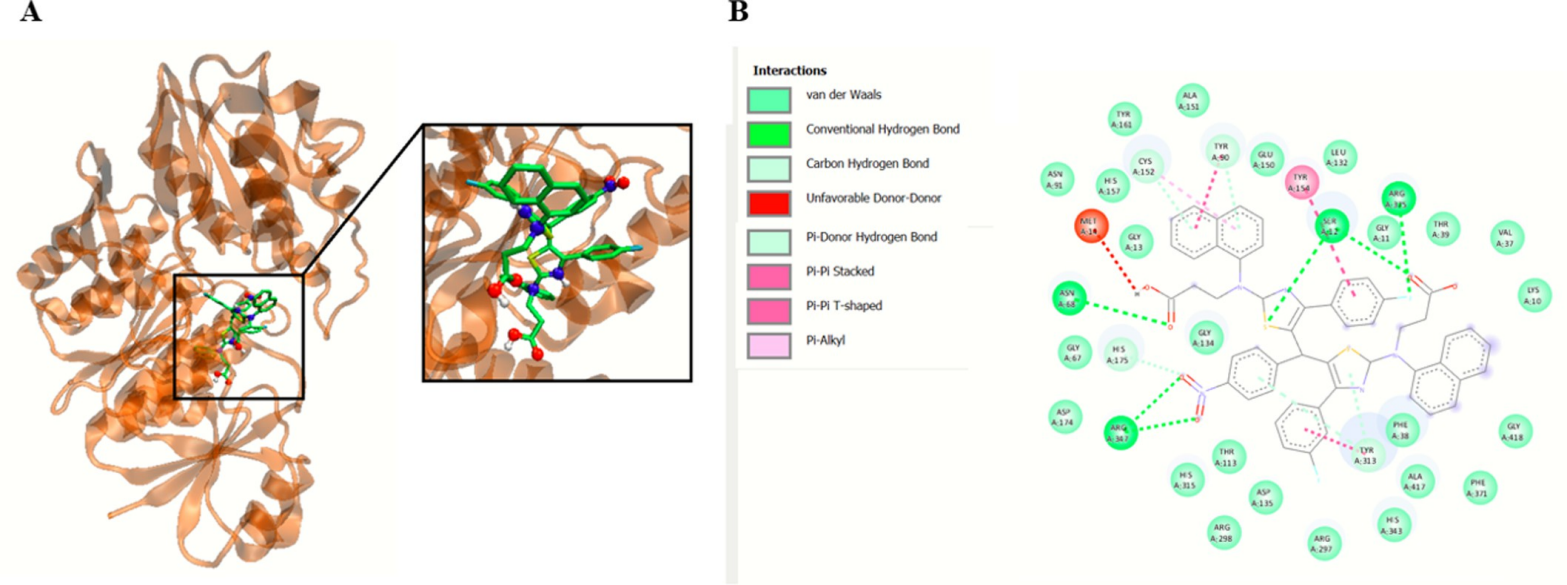
Compound 28b interacts with *S*. *aureus* MurC ligase. Panel A shows potential binding site of **28b** into MurC. Panel B demonstrates plotted 2D maps of H-bonds and hydrophobic interactions of **28b** with MurC residues. Van der Waals, Pi–Pi stacked, Pi–Pi T-shaped and Pi–alkyl is considered hydrophobic interactions.

On the other hand, compound **28b** interacts with Ser12, Asn68, Arg347 and Arg375 of MurC by forming hydrogen bonds (Fig 4A and 4B).

**Fig 5:**
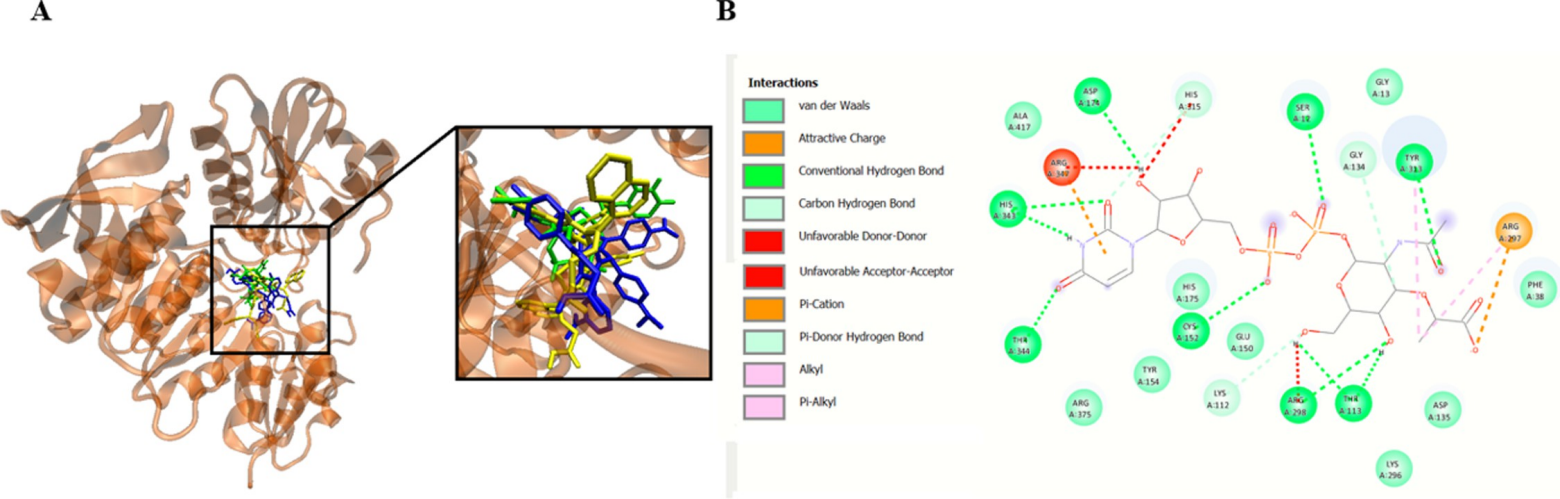
Compounds 23a and 28b shares overlapping positions with UDP-N-acetyl-alpha-D-muramate binding pocket in *S*. *aureus* MurC. Panel A shows the docking poses for UDP-N-acetyl-alpha-D-muramat**e** (green), **23a** (blue), and **28b** (yellow) into MurC. Panel B demonstrates 2D maps of H-bonds and hydrophobic interactions of UDP-N-acetyl-alpha-D-muramate with MurC amno acid residues. Van der Waals, Pi–cation, Pi–donor hydrogen bond, alkyl and Pi–alkyl are considered hydrophobic interactions.

After characterizing the interaction positions and binding of compounds **23a** and **28b** to MurC, we then explored conducted a comparative analysis of **23a** and **28b** into MurC ligase with t known ligand UDP-N-acetyl-alpha-D-muramate (Fig 5).

The molecular docking simulations elucidate the binding affinities of compounds **23a** and **28b** with *S*. *aureus* MurC in comparison to its endogenous substrate, UDP-N-acetyl-alpha-D-muramate. Evidently, both **23a** and **28b** exhibit substantial binding properties, with compound **28b** demonstrating superior efficacy (ΔGbin = -11.4 kcal/mol) relative to compound **23a** (ΔGbin = -9.6 kcal/mol). Noteworthy is the formation of hydrogen bonds by both compounds with pivotal residues within the MurC binding site, specifically Ser12 and Arg375, indicating consequential interactions essential for complex stability. Compound **28b** further establishes hydrogen bonds with Asn68 and Arg347. The substrate engages in hydrogen bonding interactions with Ser12, Thr113, Cys152, Asp174, Arg298, Tyr313, His343, and Thr344 (Table 6).

**Table 6:** Binding site contacts of compound 23a, 28b, and substrate into MurC.

Compounds	ΔG_*bin*_ (kcal/mol)	H-Bonds contacts in the Binding Site
		MurC
**23a**	−9.6	Lys 10, **Ser12**, **Arg375**
**28b**	−11.4	**Ser12**, Asn68, Arg347, **Arg375**
**Substrate** ^**[a]**^	−8.6	**Ser12**, Thr113, Cys152, Asp174, Arg298, Tyr313, His343, Thr344

[a] Substrate correspond to **UDP-N-acetyl-alpha-D-muramate**. 3D structure of the substrate was retrieved from PubChem Compound (CID 24772978) in format SDF and converted to MOL2 using open Babel software.

### Bis(thiazol-5-yl)phenylmethane derivatives demonstrate favorable *in vitro* cytotoxicity parameters

**Fig 6:**
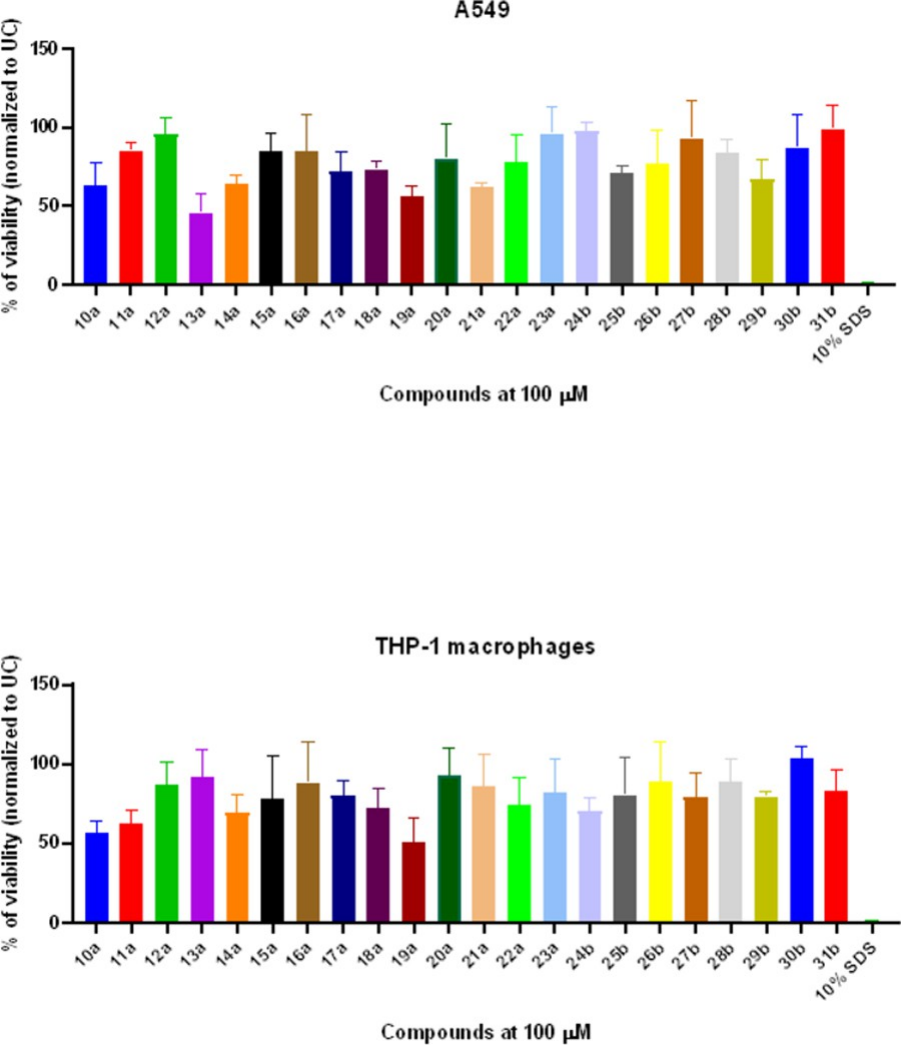
The bis(thiazol-5-yl)phenylmethane derivatives 10–31 exhibit favorable A549 and THP-1 derived macrophage cytotoxicity profiles. The data represents the mean ± standard deviation (SD) from three independent experimental replicates.

After demonstrating the potent antimicrobial and anti-biofilm activity of bis(thiazol-5-yl)phenylmethane derivatives, our investigation extended to understanding the cytotoxicity profiles of compounds **10–31**. To assess the cytotoxic properties of these compounds against different cell types, we employed A549 human lung adenocarcinoma cell lines to evaluate their effects on epithelial cells. Additionally, we utilized the THP-1 monocyte-derived macrophage model to assess cytotoxicity to phagocytic and immune cells (Fig 6).

The bis(thiazol-5-yl)phenylmethane derivatives **10–31** exhibited a structure-dependent cytotoxic activity against both A549 human lung adenocarcinoma cells and THP-1 monocyte-derived macrophages *in vitro*. Notably, the cytotoxic properties of R^2^ fluorinated compounds **(10–12)a** were influenced by the substituent at the R^1^ position. For instance, compound **10a** (R^1^-H) significantly reduced the viability of A549 cells and THP-1 macrophages to 63.4% and 53.2%, respectively. The introduction of an F radical or nitro group resulted in reduced cytotoxicity against both cell lines (Fig 3). Conversely, the introduction of chlorine at the R^2^ position and nitrile or nitro groups at R^1^ **(13–14)a** increased cytotoxicity in A549 cells (46.1% and 65.3%, respectively), while showing lower toxicity to THP-1-derived macrophages (92.1% and 69%). The substitution of chlorine with bromine at the R^2^ position of the nitrated compound **15a** decreased cytotoxicity in both cell culture models. Among compounds with a nitro group at the R^2^ position and substitutions at R^1^ **(16–19)a**, only compound **19a** (R^1^-NO_2_, R^2^-NO_2_) exhibited pronounced cytotoxicity in both cell lines. Compounds bearing a dimethylamine substituent at the R^2^ position **(20–23)a** exhibited similar cytotoxic activity regardless the substitutions at R^1^, and resulted a cell culture viability in both A549 and THP-1 models (78–112%) (Fig 6).

**Fig 7:**
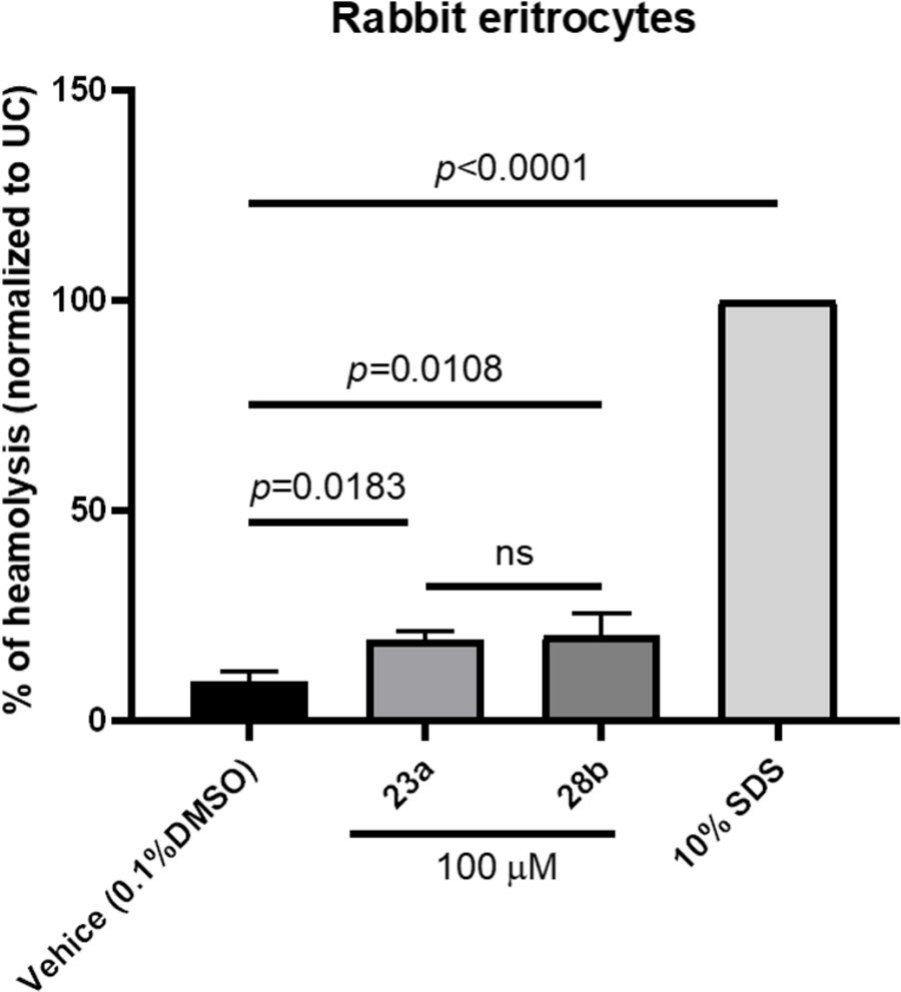
Hemolytic activity assessment of compounds 23a and 28b using rabbit erythrocyte hemolysis assay. Rabbit erythrocytes were exposed to compounds **23a** and **28b** at a concentration of 100 *μ*M for a duration of 1 hour at 37°C. Hemolytic activity was evaluated, and results were compared to the vehicle control (DMSO). The results expressed as mean values ± standard deviation (SD) are derived from three independent experimental replicates.

In contrast, compounds bearing naphthalene substitutions **(24–31)b** demonstrated low cytotoxicity profiles in both cell culture models, reducing the viability of cells to between 75% and 93%. Following the characterization of the in vitro cytotoxic properties of compounds **10–31**, we proceeded to assess the cytotoxic and hemolytic properties of the most promising antimicrobial hit compounds, **23a** and **28b**, using a rabbit erythrocyte hemolysis assay. In this assay, rabbit erythrocytes exposed to 100 *μ*M of compounds **23a** and **28b** for 1 hour at 37°C displayed significant hemolysis (*p*<0.05) compared to the vehicle (DMSO) control. However, it’s important to note that none of the compounds exhibited greater or equal hemolysis comparable to the 10% SDS control (Fig 7).

These data demonstrated that bis(thiazol-5-yl)phenylmethane derivatives 10–31 exhibit favorable *in vitro* cytotoxic profiles crucial for further hit to lead optimization.

## Discussion

In this study, the tested bis(thiazol-5-yl)phenylmethane derivatives demonstrated promising and selective antimicrobial activity against *S*. *aureus* strains with emerging and genetically defined resistance mechanism.

Within infections attributed to *S*. *aureus*, MRSA presents an even bigger challenge due to its resistance to a multiple of therapeutic agents [1, 14]. Vancomycin, commonly employed for treating MRSA infections, is facing limitations owing to the emergence of vancomycin-intermediate *Staphylococcus aureus* (VISA) and vancomycin-resistant *Staphylococcus aureus* (VRSA) strains, thereby diminishing its efficacy for critically ill patients [14, 15]. Daptomycin and oxazolidinones (such as linezolid and tedizolid) serve as alternative treatment options; however, reports of resistance emergence are already surfacing [28–30]. Consequently, there is a critical need to develop innovative candidates specifically targeting *S*. *aureus* strains with pre-existing resistance, warranting comprehensive pre-clinical evaluations.

Several small molecule compounds have been previously proposed as potential hits for targeting *Staphylococcus aureus* and other clinically significant Gram-positive pathogens [31–34]. Nitrogen-containing heterocycles, particularly those bearing various aromatic or non-aromatic structural substituents, are often regarded as favorable functional groups in medicinal chemistry, acting as pharmacophores or pharmacological mediators. Among these heterocycles, thiazoles are frequently considered favorable moieties capable of directly modulating biological activity or indirectly forming complexes that enhance the pharmacological and physicochemical properties of compounds [35]. As an illustrative example of antimicrobial thiazole derivatives, high molecular mass bis(thiazol-5-yl)phenylmethanes have been previously assessed for their antimicrobial properties [18, 19]. Despite their high molecular mass, these compounds demonstrated antimicrobial activity against tested pan-susceptible organisms, positioning them as a promising group for further exploration. In the present study, a library of multidrug-resistant Gram-positive (*S*. *aureus*) and Gram-negative (*K*. *pneumoniae* and *P*. *aeruginosa*) clinical pathogens, along with drug-resistant *C*. *auris*, was employed. Remarkably, selective Gram-positive bacteria-directed antimicrobial activity was observed. Similar activity of thiazole-based compounds against Gram-positive pathogens have been previously also reported [36, 37]. Intriguingly, the antimicrobial activity against other Gram-positive cocci, such as vancomycin-resistant *Enterococcus* or *Streptococcus*, was notably weaker. Only bis(thiazol-5-yl)phenylmethanes **17a** (R^1^-F, R^2^-NO_2_), **23a** (R^1^-NO_2_, R^2^-N(CH_3_)_2_), and **24b** (R^1^-Cl, R^2^-F) exhibited activity against *Enterococcus*, suggesting that electron-withdrawing groups like fluorine or nitro groups may play a pivotal role in the interaction with bacterial cells, while maintaining anti-staphylococcal activity. On the other hand, the incorporation of fluorine and nitro groups in the opposite positions of compound **17a** resulted in compound **12a** with loss of anti-*Enterococcal* activity, while the activity against *S*. *aureus* remained. This suggests that the location of these groups is crucial for brad-spectrum Gram-positive cocci-directed activity. Furthermore, steric hindrance-inducing substituents such as dimethylamine or chlorine appeared to enhance the compound’s interaction with *Enterococcus* cells.

Pre-existing antimicrobial resistance mechanisms in *S*. *aureus* exert a profound influence on the antimicrobial activity of various compounds that undergoing early pre-clinical screening [20]. One illustrative example is the impact of efflux pumps, which function by utilizing a myriad of compounds as substrates, actively exporting these molecules from the *S*. *aureus* cytosol [3–41]. This process can result in a consequential loss of antimicrobial activity; therefore it is crucial to understand the behavior of various investigational compounds in drug-resistant models. Additionally, drug-resistant *S*. *aureus* possesses the capability to modulate various cellular targets or enhance the expression of enzymes responsible for inactivating antimicrobial compounds therefore it is essential to develop novel compounds that are targeting novel bacterial targets, thus evading pre-existing resistance mechanisms [39]. This adaptive response contributes to a diminished activity of antimicrobial agents in drug-resistant strains as compared to their pan-susceptible counterparts.

Taking this to the account, we performed further screening of bis(thiazol-5-yl)phenylmethanes using MSSA, MRSA, and VRSA strains. Two bis(thiazol-5-yl)phenylmethane compounds emerged as promising hits, displaying consistent MIC values across diverse *S*. *aureus* resistance backgrounds. Specifically, the p-tolyl based derivative **23a**, containing nitro and dimethylamino substituents, and the naphthalene based derivative **28b**, harboring fluorine and nitro substituents, exhibited notable anti-*S*. *aureus* activity against *S*. *aureus* isolates with genetically defined resistance phenotypes such as MSSA, MRSA, and VRSA. Furthermore, naphthalene-based derivatives **12, 17** and **19** also showed good antimicrobial activity against *S*. *aureus*, although only compounds **23a** and **28b** showed strong bactericidal activity, with MIC close to minimal bactericidal concentration (MBC).

The cell wall biosynthesis pathway has been extensively explored as a prime target for antimicrobial agents. Numerous investigational and FDA-approved compounds have been introduced to combat infections caused by Gram-positive pathogens. Notably, β-lactam nucleus-containing antibiotics exemplify this approach by targeting penicillin-binding proteins (PBPs), crucial for peptidoglycan synthesis [35, 38–42]. Mutations in PBPs often lead to reduced affinity for β-lactam antibiotics, conferring bacterial resistance. Beyond PBPs, other proteins are pivotal in the cell wall synthesis of Gram-positive bacteria. Within this category, Muramyl ligases (Mur ligases) play a crucial role in peptidoglycan synthesis. The Mur ligase family encompasses distinct enzymes, including MurC, MurD, MurE, MurF, and MurG, each orchestrating specific steps in the peptidoglycan synthesis pathway. Among these, MurC catalyzes the initial addition of the amino acid L-alanine to UDP-N-acetylmuramic acid (UDP-MurNAc), a precursor for subsequent cell wall synthesis processes. Inhibition of MurC in *S*. *aureus* leads to a loss of bacterial viability and integrity, underscoring the potential of MurC as a promising antimicrobial target [19, 35, 38–41, 43]. The most promising compounds **23a** and **28b** were subjected to *in silico* modeling to propose the cellular target in *S*. *aureus*. Among Mur family proteins, MurC was identified as the most promising target with the ability to bind to active center of MurC, thus making bis(thiazol-5-yl)phenylmethanes **23a** and **28b** as novel *S*. *aureus* MurC protein modulators.

Remarkably, when examining serum binding characteristics and performing MIC evaluation using serum, compound **23a** exhibited almost complete loss of activity in the presence of serum against tested all strains, whereas compound **28b** retained its antimicrobial activity in the serum-containing environment. The p-tolyl substituent is considerably less bulky and hydrophilic, then naphthalene, thus possibly allowing compound **23a** to interact more with serum proteins. Furthermore, high lipophilicity mediated by naphthalene substituent in compound **28b** could result in stronger interaction with serum lipids thus limiting the protein binding properties.

The assessment of toxicological parameters for novel compounds is pivotal for their subsequent pre-clinical evaluation [43, 44]. In-depth *in vitro* toxicity data not only serves as a crucial determinant for guiding hit-to-lead optimization but also offers valuable insights into groups within the compounds that may mediate toxicity. Thiazole derivatives have been extensively investigated as potential anti-cancer candidates, implying that compounds containing the thiazole nucleus may possess inherent cytotoxic properties. In this context, the evaluation of cytotoxic properties of bis(thiazol-5-yl)phenylmethanes was undertaken, utilizing lung epithelial cell models and monocyte-derived macrophages. Surprisingly, the bis(thiazol-5-yl)phenylmethanes exhibited weak cytotoxic activity in both cellular models. To further elucidate the detergent-like cytotoxic activity the most promising compounds (**23a** and **28b**) underwent evaluation using a rabbit erythrocyte lysis assay [43]. Both compounds induced lysis of rabbit erythrocytes compared to the vehicle control. However, it is noteworthy that the extent of lysis was significantly lower when compared to the sodium dodecyl sulfate control, a known detergent with strong membrane-disrupting properties.

These results suggest that the cytotoxic activity of bis(thiazol-5-yl)phenylmethanes, although present, may not be predominantly mediated by detergent-like mechanisms [43]. The comprehensive evaluation of their cytotoxicity across different cell models and the confirmation of a milder erythrocyte lysis profile, as compared to the control, emphasize the need for a nuanced understanding of the mechanisms underlying the observed cytotoxic properties. Further investigations into the specific cellular pathways impacted by these compounds and their potential therapeutic implications will be essential for their progression in pre-clinical development.

This study provides structure-activity relation information on novel class of bis(thiazol-5-yl)phenylmethanes and sheds some light on their future evaluation as antimicrobials or anti-biofilm candidates with activity spanning across different resistance phenotypes. Further studies are needed to understand the safety, *in vivo* activity and pharmacological properties of the most promising bis(thiazol-5-yl)phenylmethanes and their inhibitory properties directed towards *S*. *aureus* MurC.

## Supporting information

S1 File (DOCX)

## References

[1] Bengtsson-Palme J, Kristiansson E, Larsson DGJ. Environmental factors influencing the development and spread of antibiotic resistance. FEMS Microbiol Rev. 2018 Jan 1;42(1):fux053. doi: 10.1093/femsre/fux053 ; PMCID: PMC5812547. 29069382 PMC5812547

[2] Larsson DGJ, Flach CF. Antibiotic resistance in the environment. Nat Rev Microbiol. 2022 May;20(5):257–269. doi: 10.1038/s41579-021-00649-x Epub 2021 Nov 4. ; PMCID: PMC8567979. 34737424 PMC8567979

[3] Jit M, Ng DHL, Luangasanatip N, Sandmann F, Atkins KE, Robotham JV, et al. Quantifying the economic cost of antibiotic resistance and the impact of related interventions: rapid methodological review, conceptual framework and recommendations for future studies. BMC Med. 2020 Mar 6;18(1):38. doi: 10.1186/s12916-020-1507-2 ; PMCID: PMC7059710. 32138748 PMC7059710

[4] Martens E, Demain AL. The antibiotic resistance crisis, with a focus on the United States. J Antibiot (Tokyo). 2017 May;70(5):520–526. doi: 10.1038/ja.2017.30 Epub 2017 Mar 1. . 28246379

[5] Lakhundi S, Zhang K. Methicillin-resistant *Staphylococcus aureus*: Molecular characterization, evolution, and epidemiology. Clin Microbiol Rev. 2018 Sep 12;31(4):e00020–18. doi: 10.1128/CMR.00020-18 ; PMCID: PMC6148192. 30209034 PMC6148192

[6] Jaradat ZW, Ababneh QO, Sha’aban ST, Alkofahi AA, Assaleh D, Al Shara A. Methicillin resistant *Staphylococcus aureus* and public fomites: a review. Pathog Glob Health. 2020 Dec;114(8):426–450. doi: 10.1080/20477724.2020.1824112 Epub 2020 Oct 28. ; PMCID: PMC7759291. 33115375 PMC7759291

[7] Menard G, Silard C, Suriray M, Rouillon A, Augagneur Y. Thirty years of sRNA-mediated regulation in *Staphylococcus aureus*: From initial discoveries to *in vivo* biological implications. Int J Mol Sci. 2022 Jul 1;23(13):7346. doi: 10.3390/ijms23137346 ; PMCID: PMC9266662. 35806357 PMC9266662

[8] Oliveira WF, Silva PMS, Silva RCS, Silva GMM, Machado G, Coelho LCBB, et al. *Staphylococcus aureus* and *Staphylococcus epidermidis* infections on implants. J Hosp Infect. J Hosp Infect. 2018 Feb;98(2):111–117. doi: 10.1016/j.jhin.2017.11.008 Epub 2017 Nov 22. 29175074

[9] Bhattacharya M, Wozniak DJ, Stoodley P, Hall-Stoodley L. Prevention and treatment of *Staphylococcus aureus* biofilms. Expert Rev Anti Infect Ther. 2015;13(12):1499–516. doi: 10.1586/14787210.2015.1100533 Epub 2015 Nov 13. ; PMCID: PMC5142822. 26646248 PMC5142822

[10] Lister JL, Horswill AR. *Staphylococcus aureus* biofilms: recent developments in biofilm dispersal. Front Cell Infect Microbiol. 2014 Dec 23;4:178. doi: 10.3389/fcimb.2014.00178 ; PMCID: PMC4275032. 25566513 PMC4275032

[11] Cassat JE, Thomsen I. *Staphylococcus aureus* infections in children. Curr Opin Infect Dis. 2021 Oct 1;34(5):510–518. doi: 10.1097/QCO.0000000000000752 ; PMCID: PMC8630804. 34524201 PMC8630804

[12] Zhou K, Li C, Chen D, Pan Y, Tao Y, Qu W, et al. A review on nanosystems as an effective approach against infections of *Staphylococcus aureus*. Int J Nanomedicine. 2018 Nov 9;13:7333–7347. doi: 10.2147/IJN.S169935 ; PMCID: PMC6233487. 30519018 PMC6233487

[13] Arunachalam K, Pandurangan P, Shi C, Lagoa R. Regulation of *Staphylococcus aureus* virulence and application of nanotherapeutics to eradicate S. *aureus* infection. Pharmaceutics. 2023 Jan 17;15(2):310. doi: 10.3390/pharmaceutics15020310 ; PMCID: PMC9960757. 36839634 PMC9960757

[14] Petraitis V, Petraitiene R, Kavaliauskas P, Naing E, Garcia A, Sutherland C, et al. Pharmacokinetics, Tissue Distribution, and Efficacy of VIO-001 (Meropenem/Piperacillin/Tazobactam) for Treatment of Methicillin-Resistant Staphylococcus aureus Bacteremia in Immunocompetent Rabbits with Chronic Indwelling Vascular Catheters. Antimicrob Agents Chemother. 2021 Oct 18;65(11):e0116821. doi: 10.1128/AAC.01168-21 Epub 2021 Aug 30. ; PMCID: PMC8522724. 34460301 PMC8522724

[15] McGuinness WA, Malachowa N, DeLeo FR. Vancomycin resistance in *Staphylococcus aureus*. Yale J Biol Med. 2017 Jun 23;90(2):269–281. ; PMCID: PMC5482303. 28656013 PMC5482303

[16] Borcea AM, Ionuț I, Crișan O, Oniga O. An overview of the synthesis and antimicrobial, antiprotozoal, and antitumor activity of thiazole and bisthiazole derivatives. Molecules. 2021 Jan 25;26(3):624. doi: 10.3390/molecules26030624 ; PMCID: PMC7865802. 33504100 PMC7865802

[17] Khamitova А, Berillo D, Lozynskyi A, Konechnyi Y, Mural D, Georgiyants V, et al. Thiadiazole and Thiazole Derivatives as Potential Antimicrobial Agents. Mini Rev Med Chem. 2023 Jul 13. doi: 10.2174/1389557523666230713115947 Epub ahead of print. . 37448365

[18] Grybaitė B, Jonuškienė I, Vaickelionienė R, Mickevičius V. Synthesis, transformation and antibacterial activity of new N,N-disubstituted 2-aminothiazole derivatives. Chemija 2017 Dec 7;28(1):73–64.

[19] Grybaitė B, Vaickelionienė R, Stasevych M, Komarovska-Porokhnyavets O, Novikov V, Mickevičius V. Synthesis, transformation of 3-[(4-arylthiazol-2-yl)- (p-tolyl)amino]propanoic acids, bis(thiazol-5-yl)phenyl-, bis(thiazol-5-yl)methane derivatives, and their antimicrobial activity. Heterocycles 2017 Nov 29;96(1):105–86.

[20] Kavaliauskas P, Grybaite B, Mickevicius V, Petraitiene R, Grigaleviciute R, Planciuniene R, et al. Synthesis, ADMET Properties, and *in vitro* antimicrobial and antibiofilm activity of 5-nitro-2-thiophenecarbaldehyde N-((E)-(5-nitrothienyl)methylidene)hydrazone (KTU-286) against *Staphylococcus aureus* with defined resistance mechanisms. Antibiotics (Basel). 2020 Sep 17;9(9):612. doi: 10.3390/antibiotics9090612 ; PMCID: PMC7558474. 32957471 PMC7558474

[21] Hanwell MD, Curtis DE, Lonie DC, Vandermeersch T, Zurek E, Hutchison GR. Avogadro: an advanced semantic chemical editor, visualization, and analysis platform. J Cheminform. 2012 Aug 13;4(1):17. doi: 10.1186/1758-2946-4-17 ; PMCID: PMC3542060. 22889332 PMC3542060

[22] Trott O, Olson AJ. AutoDock Vina: improving the speed and accuracy of docking with a new scoring function, efficient optimization, and multithreading. J Comput Chem. 2010 Jan 30;31(2):455–61. doi: 10.1002/jcc.21334 ; PMCID: PMC3041641. 19499576 PMC3041641

[23] Humphrey W, Dalke A, Schulten K. VMD: visual molecular dynamics. J Mol Graph. 1996 Feb;14(1):33–8, 27–8. doi: 10.1016/0263-7855(96)00018-5 . 8744570

[24] Kairytė K, Grybaitė B, Vaickelionienė R, Sapijanskaitė-Banevič B, Kavaliauskas P, Mickevičius V. Synthesis and biological activity characterization of novel 5-oxopyrrolidine derivatives with promising anticancer and antimicrobial activity. Pharmaceuticals (Basel). 2022 Aug 6;15(8):970. doi: 10.3390/ph15080970 ; PMCID: PMC9415606. 36015119 PMC9415606

[25] Minickaitė R, Grybaitė B, Vaickelionienė R, Kavaliauskas P, Petraitis V, Petraitienė R, et al. Synthesis of novel aminothiazole derivatives as promising antiviral, antioxidant and antibacterial candidates. Int J Mol Sci. 2022 Jul 12;23(14):7688. doi: 10.3390/ijms23147688 ; PMCID: PMC9319503. 35887038 PMC9319503

[26] Kavaliauskas P, Opazo FS, Acevedo W, Petraitiene R, Grybaitė B, Anusevičius K, et al. Synthesis, biological activity, and molecular modelling studies of naphthoquinone derivatives as promising anticancer candidates targeting COX-2. Pharmaceuticals (Basel). 2022 Apr 27;15(5):541. doi: 10.3390/ph15050541 ; PMCID: PMC9144205. 35631366 PMC9144205

[27] Rodgus J, Prakapaite R, Mitsidis P, Grigaleviciute R, Planciuniene R, Kavaliauskas P, et al. Molecular epidemiology of Group B streptococci in Lithuania identifies multi-drug resistant clones and sporadic ST1 serotypes Ia and Ib. Pathogens. 2022 Sep 17;11(9):1060. doi: 10.3390/pathogens11091060 ; PMCID: PMC9504518. 36145492 PMC9504518

[28] de Carvalho CCCR, Taglialegna A, Rosato AE. Impact of PrsA on membrane lipid composition during daptomycin-resistance-mediated β-lactam sensitization in clinical MRSA strains. J Antimicrob Chemother. 2021 Dec 24;77(1):135–147. doi: 10.1093/jac/dkab356 ; PMCID: PMC8730685. 34618036 PMC8730685

[29] Pee CJE, Pader V, Ledger EVK, Edwards AM. A FASII inhibitor prevents staphylococcal evasion of daptomycin by inhibiting phospholipid decoy production. Antimicrob Agents Chemother. 2019 Mar 27;63(4):e02105–18. doi: 10.1128/AAC.02105-18 ; PMCID: PMC6496159. 30718253 PMC6496159

[30] Mlynarczyk-Bonikowska B, Kowalewski C, Krolak-Ulinska A, Marusza W. Molecular mechanisms of drug resistance in *Staphylococcus aureus*. Int J Mol Sci. 2022 Jul 22;23(15):8088. doi: 10.3390/ijms23158088 ; PMCID: PMC9332259. 35897667 PMC9332259

[31] Gao P, Ho PL, Yan B, Sze KH, Davies J, Kao RYT. Suppression of *Staphylococcus aureus* virulence by a small-molecule compound. Proc Natl Acad Sci U S A. 2018 Jul 31;115(31):8003–8008. doi: 10.1073/pnas.1720520115 Epub 2018 Jul 16. ; PMCID: PMC6077739. 30012613 PMC6077739

[32] Jing S, Kong X, Wang L, Wang H, Feng J, Wei L, et al. Quercetin reduces the virulence of S. *aureus* by targeting ClpP to protect mice from MRSA-induced lethal pneumonia. Microbiol Spectr. 2022 Apr 27;10(2):e0234021. doi: 10.1128/spectrum.02340-21 Epub 2022 Mar 23. ; PMCID: PMC9045277. 35319277 PMC9045277

[33] Cai X, Zheng W, Li Z. High-throughput screening strategies for the development of anti-virulence inhibitors against *Staphylococcus aureus*. Curr Med Chem. 2019;26(13):2297–2312. doi: 10.2174/0929867324666171121102829 . 29165063

[34] Tao Y, Sun D, Ren X, Zhao Y, Zhang H, Jiang T, et al. Bavachin suppresses alpha-hemolysin expression and protects mice from pneumonia infection by *Staphylococcus aureus*. J Microbiol Biotechnol. 2022 Oct 28;32(10):1253–1261. doi: 10.4014/jmb.2207.07048 Epub 2022 Sep 14. ; PMCID: PMC9668093. 36224757 PMC9668093

[35] Haroun M, Tratrat C, Tsolaki E, Geronikaki A. Thiazole-based thiazolidinones as potent antimicrobial agents: Design, synthesis and biological evaluation. Comb Chem High Throughput Screen. 2016;19(1):51–7. doi: 10.2174/1386207319666151203002348 . 26632442

[36] Ghasemi B, Sanjarani G, Sanjarani Z, Majidiani H. Evaluation of anti-bacterial effects of some novel thiazole and imidazole derivatives against some pathogenic bacteria. Iran J Microbiol. 2015 Oct;7(5):281–6. doi: 10.1016/B978-0-12-801238-3.00141-0 ; PMCID: PMC4695510. 26719785 PMC4695510

[37] Guo J, Xie Z, Ruan W, Tang Q, Qiao D, Zhu W. Thiazole-based analogues as potential antibacterial agents against methicillin-resistant *Staphylococcus aureus* (MRSA) and their SAR elucidation. Eur J Med Chem. 2023 Nov 5;259:115689. doi: 10.1016/j.ejmech.2023.115689 Epub 2023 Jul 29. . 37542993

[38] Chen XY, Qian F, Wang YY, Liu Y, Sun Y, Zha WB, et al. Ginsenoside 20(S)-Rh2 promotes cellular pharmacokinetics and intracellular antibacterial activity of levofloxacin against *Staphylococcus aureus* through drug efflux inhibition and subcellular stabilization. Acta Pharmacol Sin. 2021 Nov;42(11):1930–1941. doi: 10.1038/s41401-021-00751-z Epub 2021 Aug 30. ; PMCID: PMC8564512. 34462563 PMC8564512

[39] Schindler BD, Jacinto P, Kaatz GW. Inhibition of drug efflux pumps in *Staphylococcus aureus*: current status of potentiating existing antibiotics. Future Microbiol. 2013 Apr;8(4):491–507. doi: 10.2217/fmb.13.16 . 23534361

[40] Monteiro KLC, de Aquino TM, Mendonça Junior FJB. An update on *Staphylococcus aureus* NorA efflux pump inhibitors. Curr Top Med Chem. 2020;20(24):2168–2185. doi: 10.2174/1568026620666200704135837 . 32621719

[41] Faillace MS, Alves Borges Leal AL, Araújo de Oliveira Alcântara F, Ferreira JHL, de Siqueira-Júnior JP, Sampaio Nogueira CE, et al. Inhibition of the NorA efflux pump of *S*. *aureus* by (Z)-5-(4-fluorobenzylidene)-imidazolidines. Bioorg Med Chem Lett. 2021 Jan 1;31:127670. doi: 10.1016/j.bmcl.2020.127670 Epub 2020 Nov 6. . 33161124

[42] Pantosti A, Sanchini A, Monaco M. Mechanisms of antibiotic resistance in *Staphylococcus aureus*. Future Microbiol. 2007 Jun;2(3):323–34. doi: 10.2217/17460913.2.3.323 . 17661706

[43] Evans BC, Nelson CE, Yu SS, Beavers KR, Kim AJ, Li H, et al. *Ex vivo* red blood cell hemolysis assay for the evaluation of pH-responsive endosomolytic agents for cytosolic delivery of biomacromolecular drugs. J Vis Exp. 2013 Mar 9;(73):e50166. doi: 10.3791/50166 ; PMCID: PMC3626231. 23524982 PMC3626231

[44] Lee G, Karunanithi S, Posner B, Niederstrasser H, Cheng H, Federov Y, et al. Chemical screening identifies novel small molecule activators of natural killer cell cytotoxicity against cancer cells. Cancer Immunol Immunother. 2022 Jul;71(7):1671–1680. doi: 10.1007/s00262-021-03117-w Epub 2021 Nov 23. ; PMCID: PMC9124724. 34816323 PMC9124724

