# Supplementary material for: Exploring the potential of bis(thiazol-5-yl)phenylmethane derivatives as novel candidates against genetically defined multidrug-resistant Staphylococcus aureus
Source: PLoS One. 2024 Mar 22;19(3):e0300380. doi: 10.1371/journal.pone.0300380 (PMC10959338; doi:10.1371/journal.pone.0300380)
Supplement: S1 File — (DOCX) [file pone.0300380.s001.docx]

**Exploring the potential of bis(thiazol-5-yl)phenylmethane derivatives as**

**novel candidates against genetically defined multidrug-resistant**

***Staphylococcus aureus* and biofilms**

Povilas Kavaliauskas^1,2,3,4,*^, Waldo Acevedo^6^, Andrew Garcia^1^, Ethan Naing^1^, Birute Grybaite^4^, Birute Sapijanskaite-Banevic^4^, Ramune Grigaleviciute^3,5^, Ruta Petraitiene^1,2^, Vytautas Mickevicius^4¶^, Vidmantas Petraitis ^1,2,3¶^.

^1^ Division of Infectious Diseases, Department of Medicine, Weill Cornell Medicine of Cornell University, New York, NY, USA.

^2^ Institute of Infectious Diseases and Pathogenic Microbiology, Prienai, Lithuania

^3^ Biological Research Center, Lithuanian University of Health Sciences, Kaunas, Lithuania.

^4^ Department of Organic Chemistry, Kaunas University of Technology, Kaunas, Lithuania.

^5^ Department of Animal Nutrition, Lithuanian University of Health sciences, Kaunas, Lithuania.

^6^ Instituto de Química, Facultad de Ciencias, Pontificia Universidad Católica de Valparaíso, Valparaíso, Chile.

* Corresponding author

Email: pok4001@med.cornell.edu

^¶^ These authors contributed equally

**Table of Contents**

[I. NMR Spectra (7b, 8b, (24-31)b) (All in DMSO-*d_6_*) 3](#_Toc156223559)

[II. Mass spectra of synthesized compounds (24-31)b 14](#_Toc156223560)

[III. IR spectra of synthesized compounds (24-31)b 18](#_Toc156223561)

[IV. In vitro antifungal activity 23](#_Toc156223562)

1. **NMR Spectra (7b, 8b, (24-31)b) (All in DMSO-d_6_)**


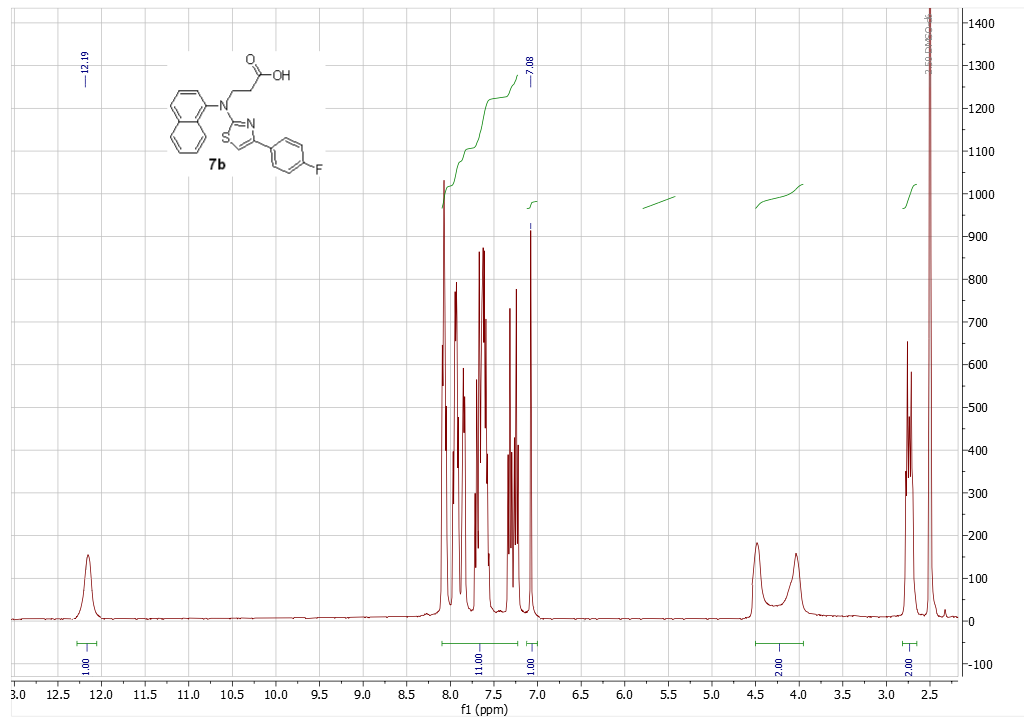


*Figure S1: ^1^H NMR of compound* ***7b***


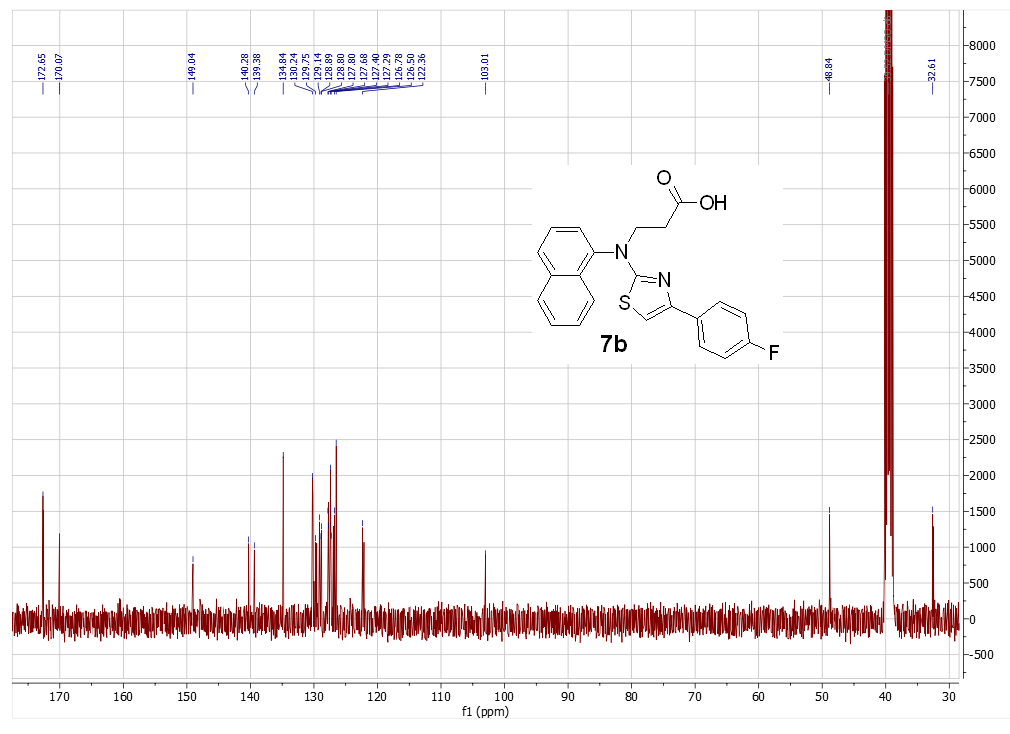


*Figure S2: ^13^C NMR of compound* ***7b***


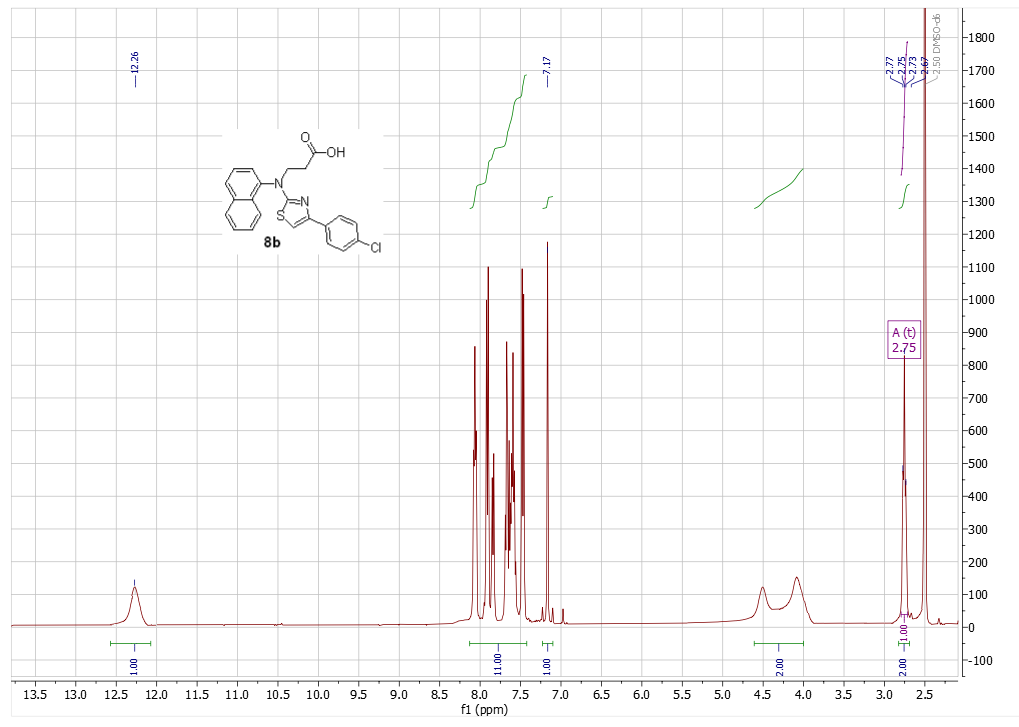


*Figure S3: ^1^H NMR of compound* ***8b***

*
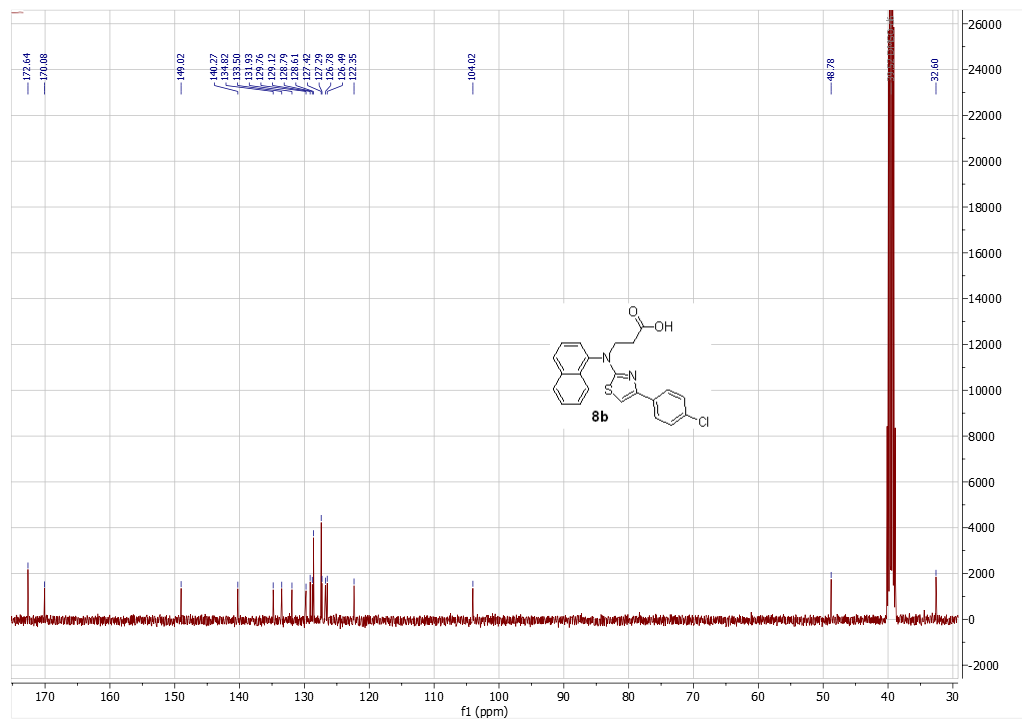
*

*Figure S4: ^13^C NMR of compound* ***8b***


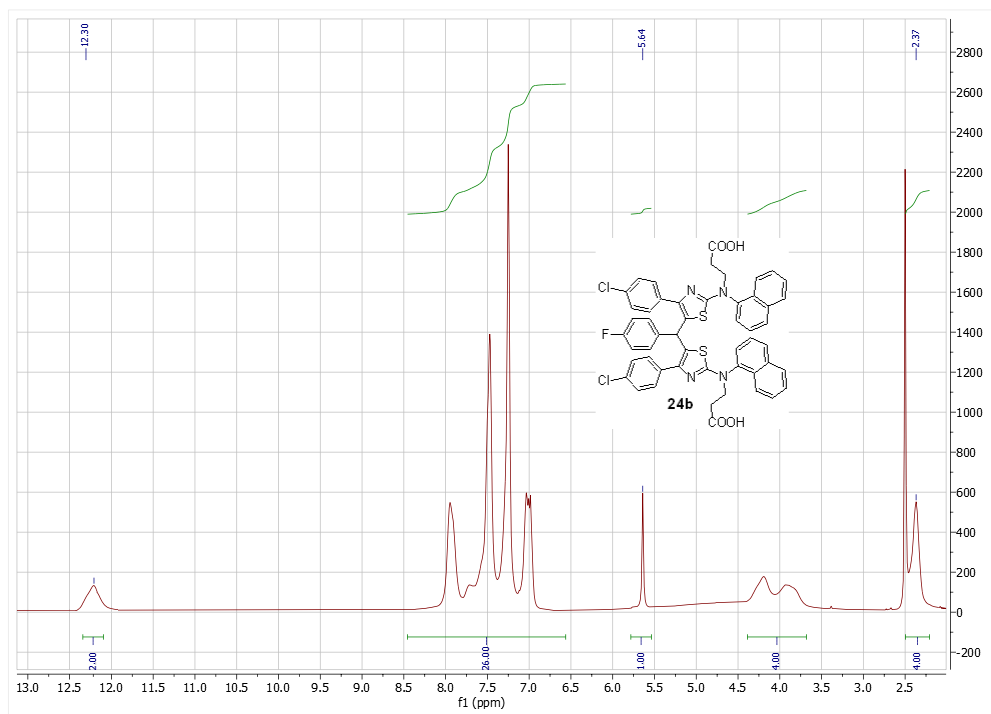


*Figure S5: ^1^H NMR of compound* ***24b***

***
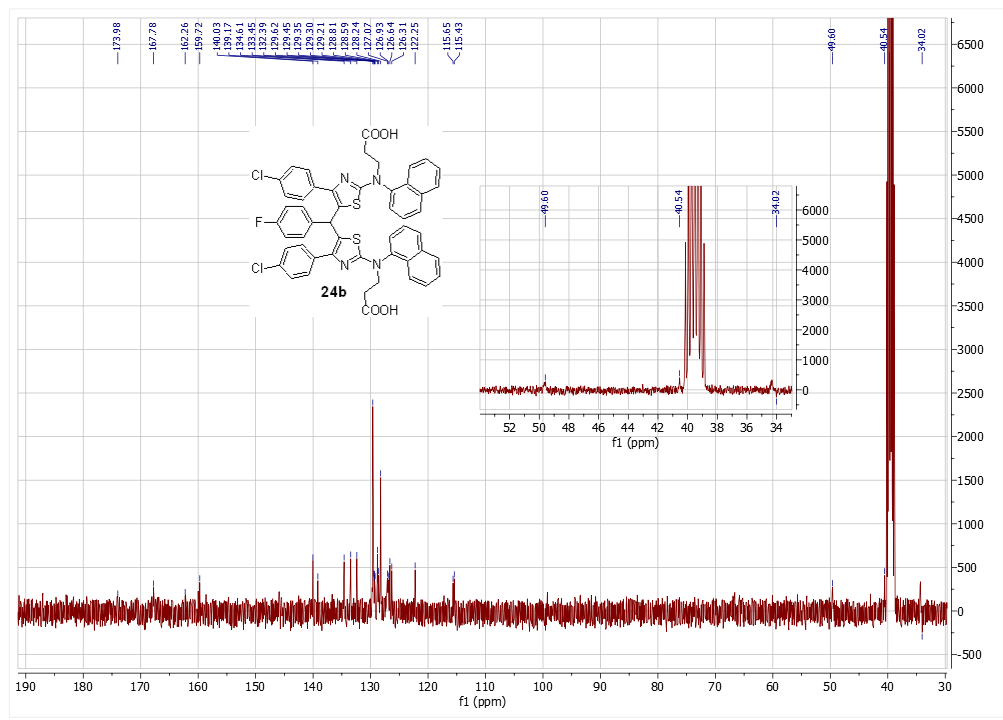
***

*Figure S6: ^13^C NMR of compound* ***24b***

**
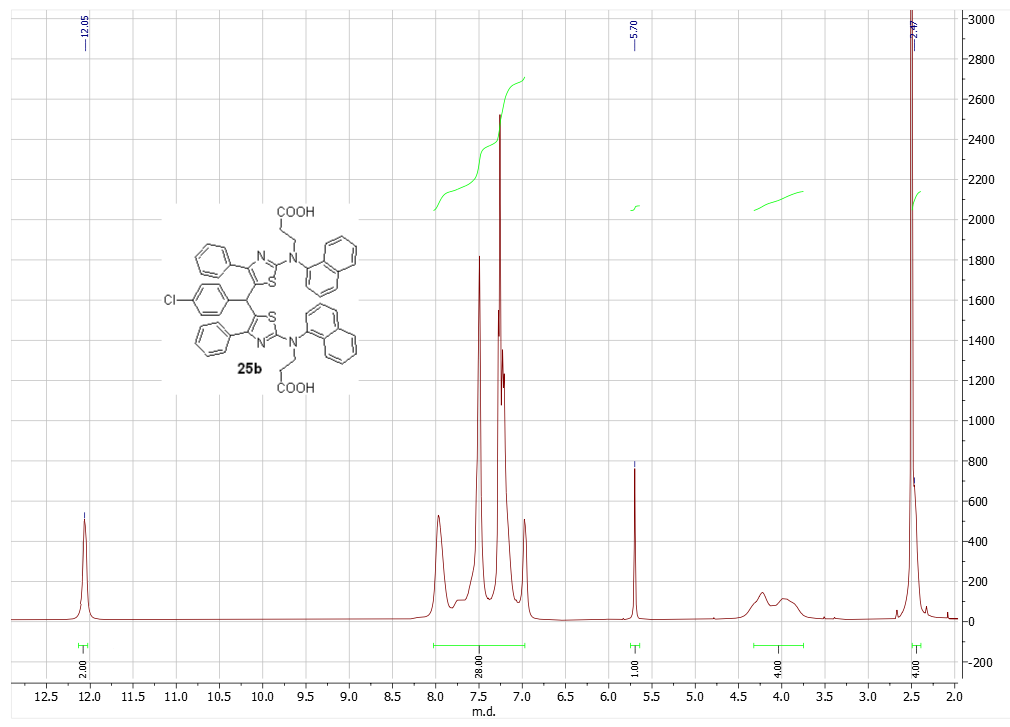
**

*Figure S7: ^1^H NMR of compound* ***25b***

***
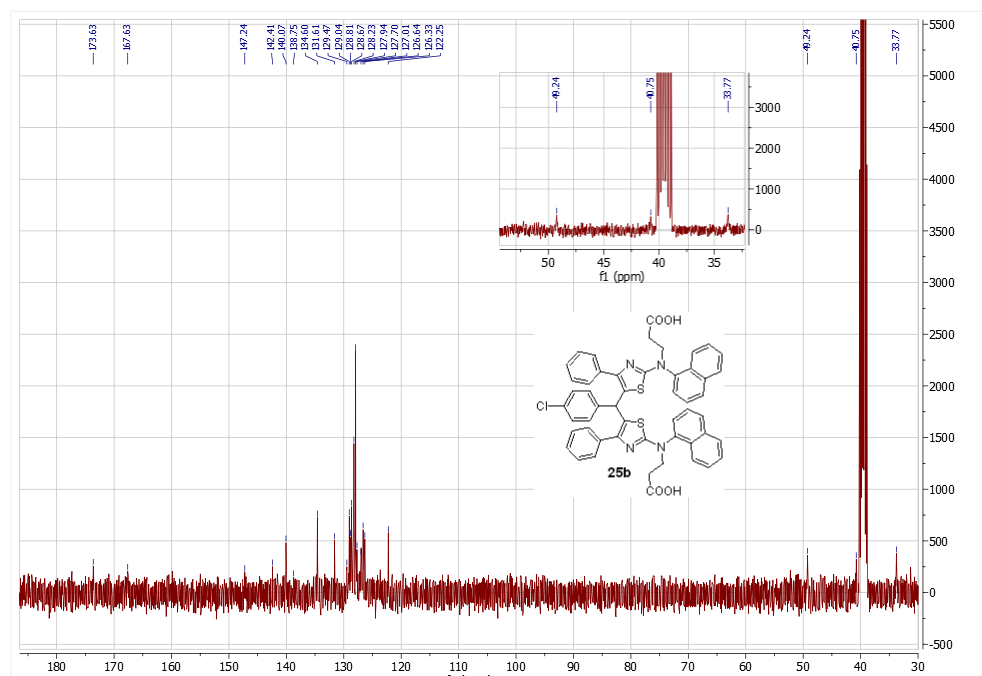
***

*Figure S8: ^13^C NMR of compound* ***25b***

***
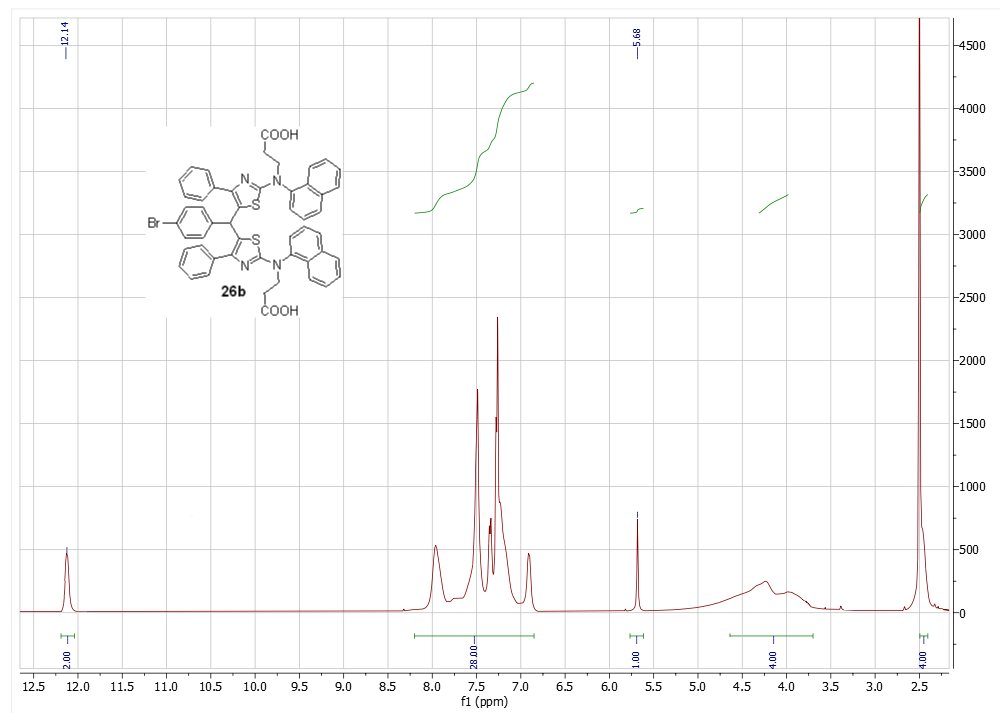
***

*Figure S9: ^1^H NMR of compound* ***26b***

***
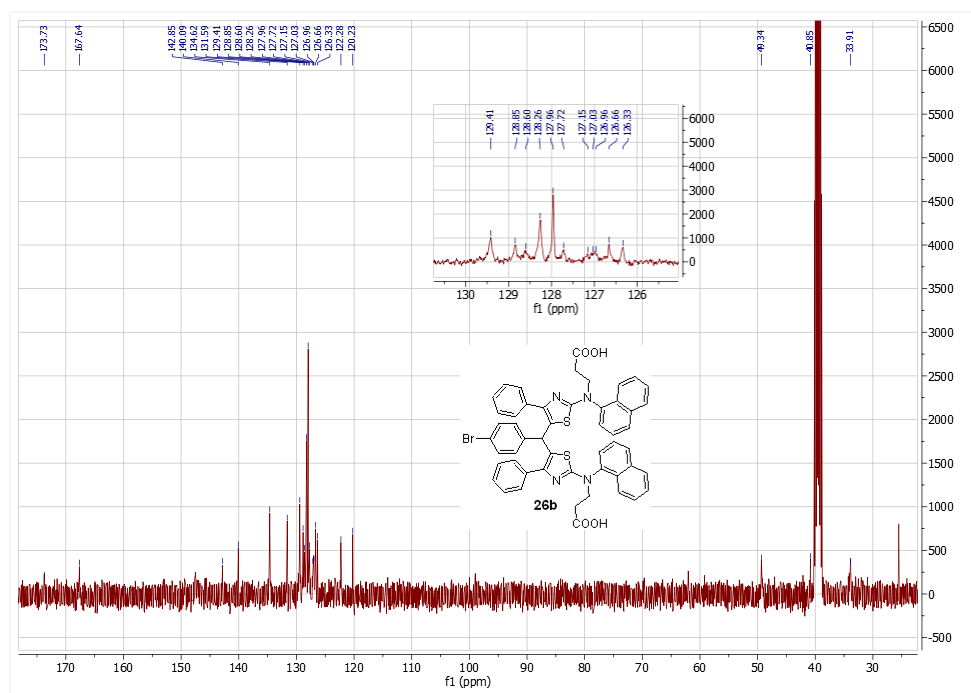
***

*Figure S10: ^13^C NMR of compound* ***26b***

***
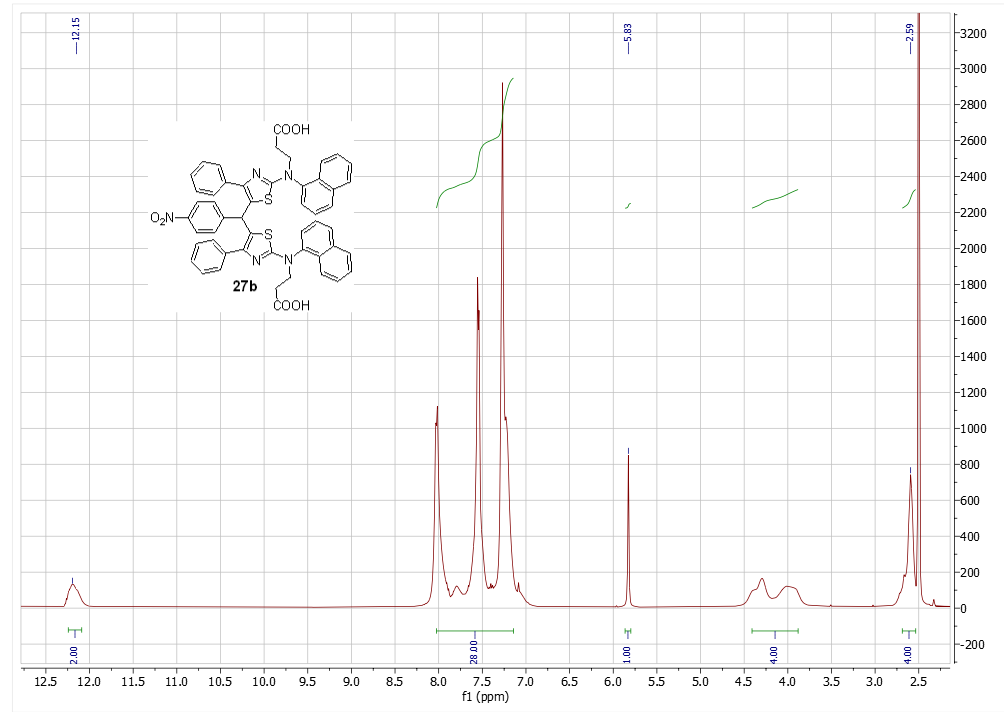
***

*Figure S11: ^1^H NMR of compound* ***27b***

***
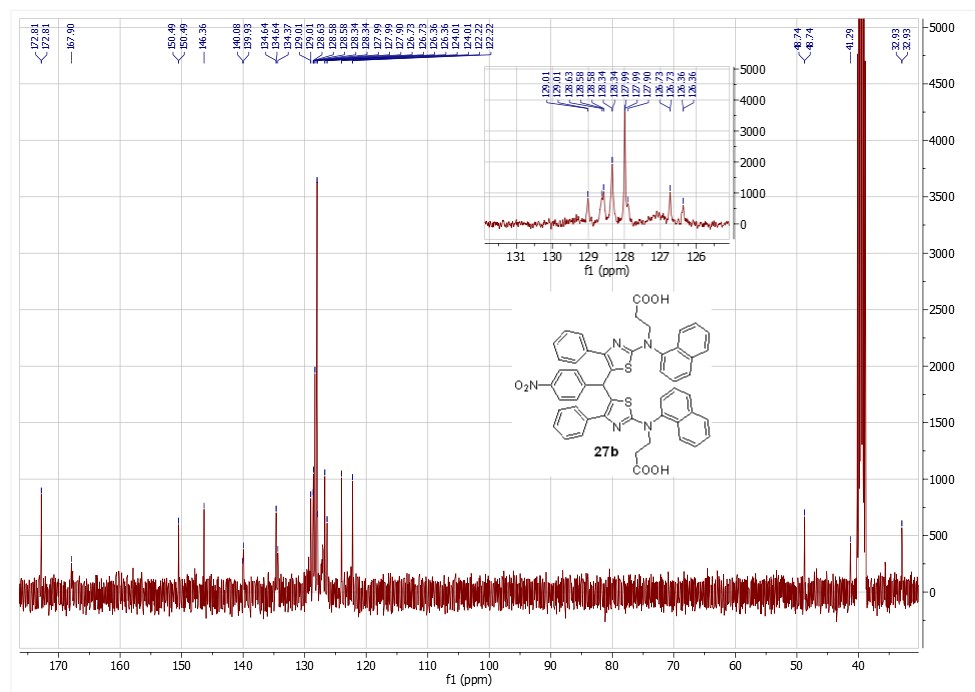
***

*Figure S12: ^13^C NMR of compound* ***27b***

***
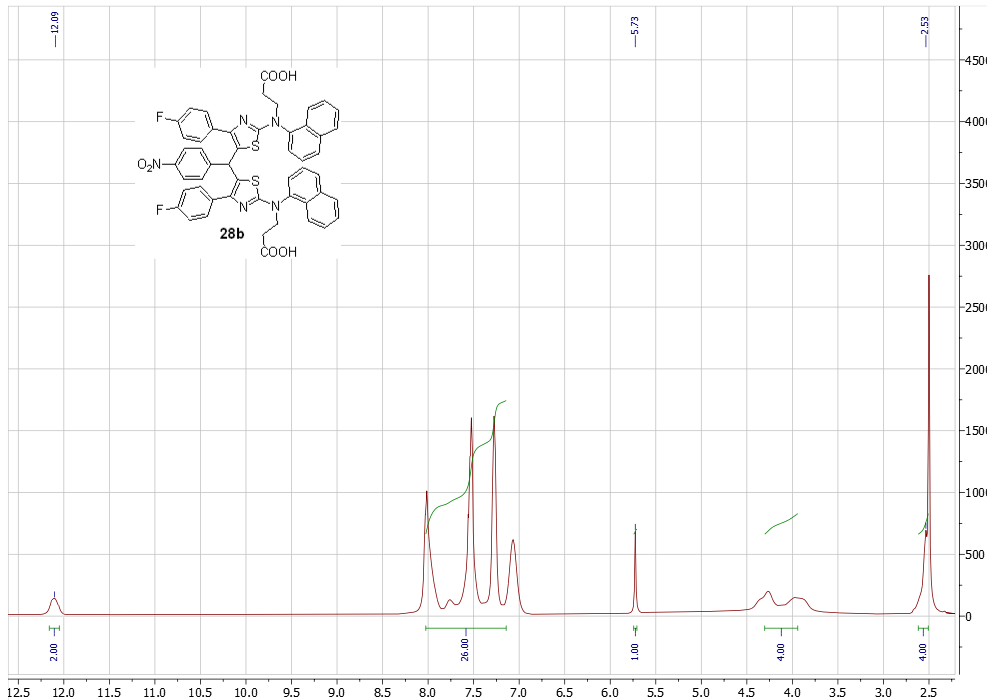
***

*Figure S13: ^1^H NMR of compound* ***28b***

***
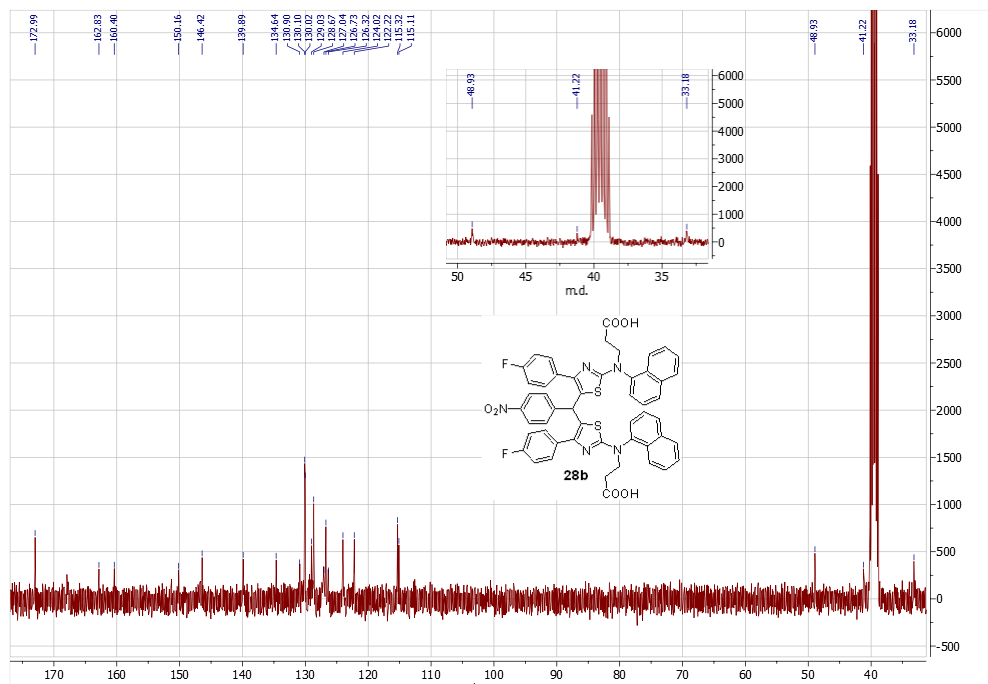
***

*Figure S14: ^13^C NMR of compound* ***28b***

*
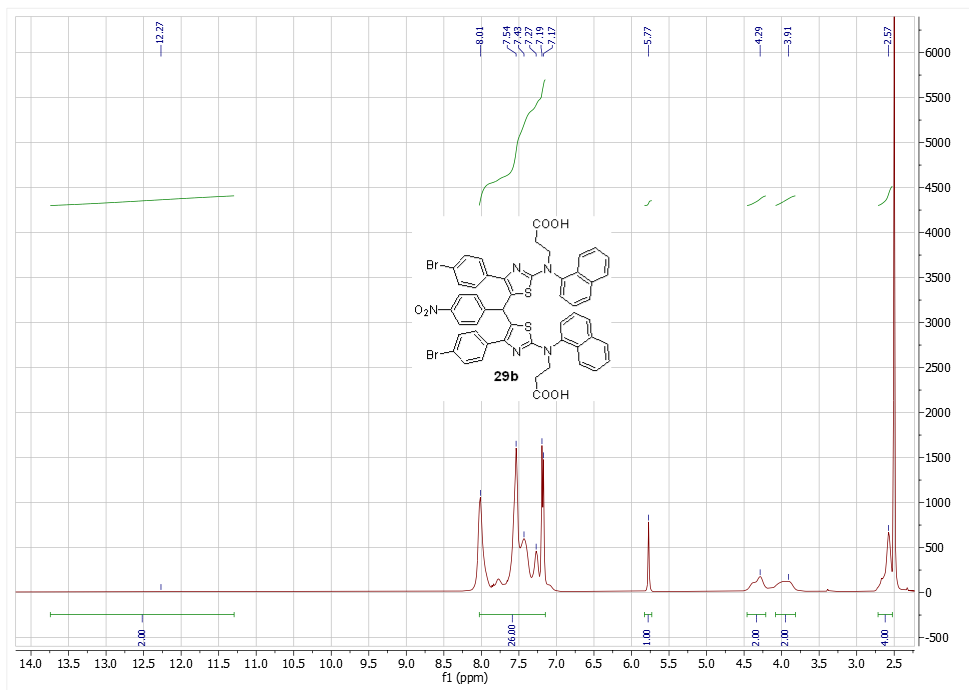
*

*Figure S15: ^1^H NMR of compound* ***29b***

***
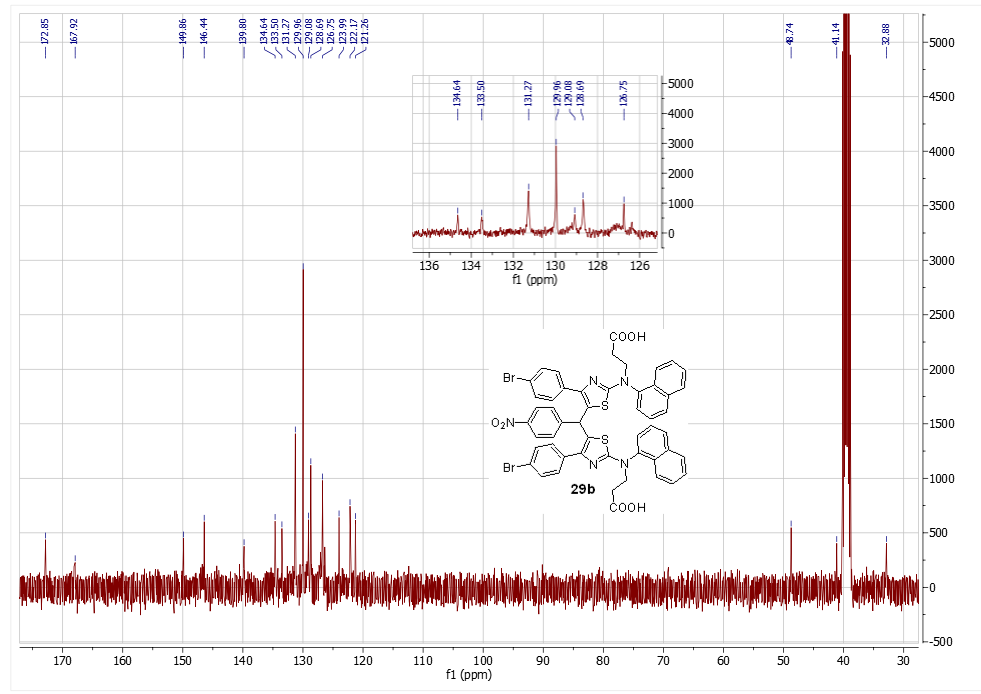
***

*Figure S16: ^13^C NMR of compound* ***29b***

***
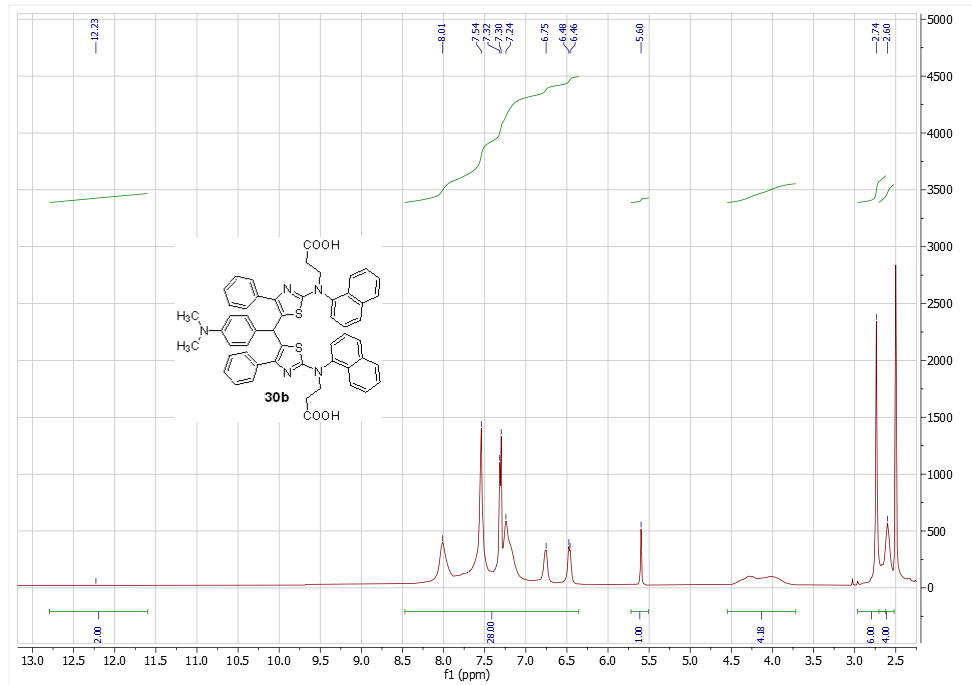
***

*Figure S17: ^1^H NMR of compound* ***30b***

***
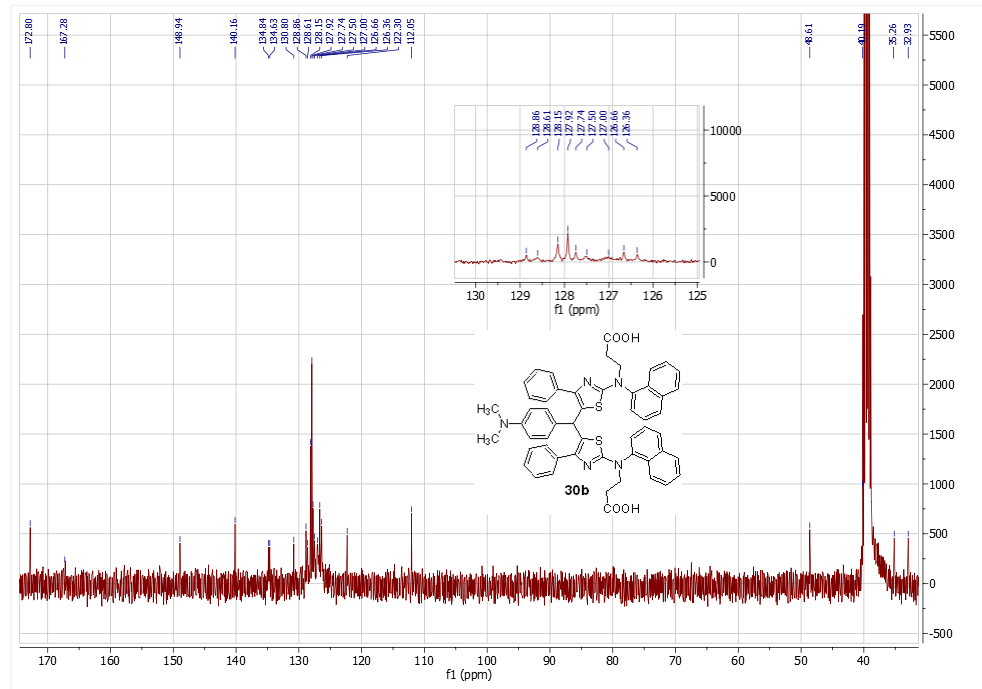
***

*Figure S18: ^13^C NMR of compound* ***30b***

***
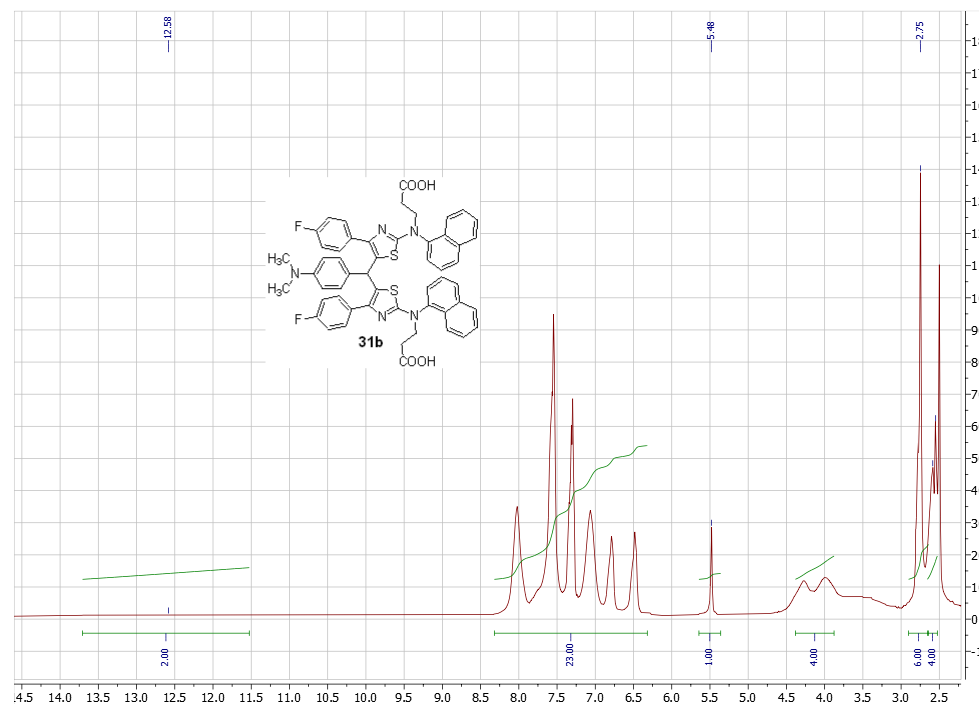
***

*Figure S19: ^1^H NMR of compound* ***31b***

***
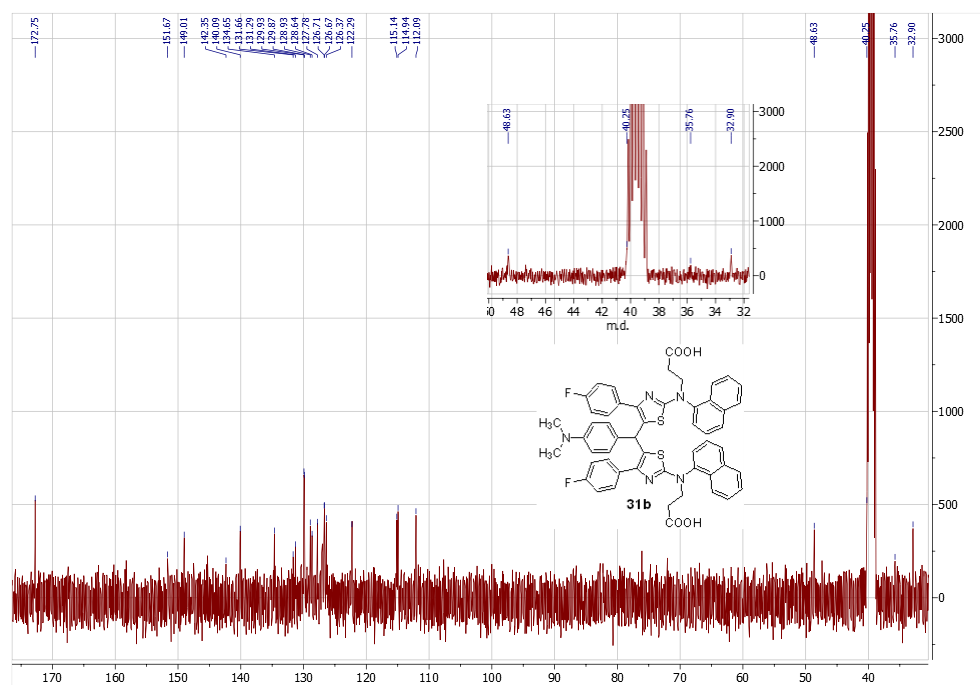
***

*Figure S20: ^13^C NMR of compound* ***31b***

1. **Mass spectra of synthesized compounds (24-31)b**


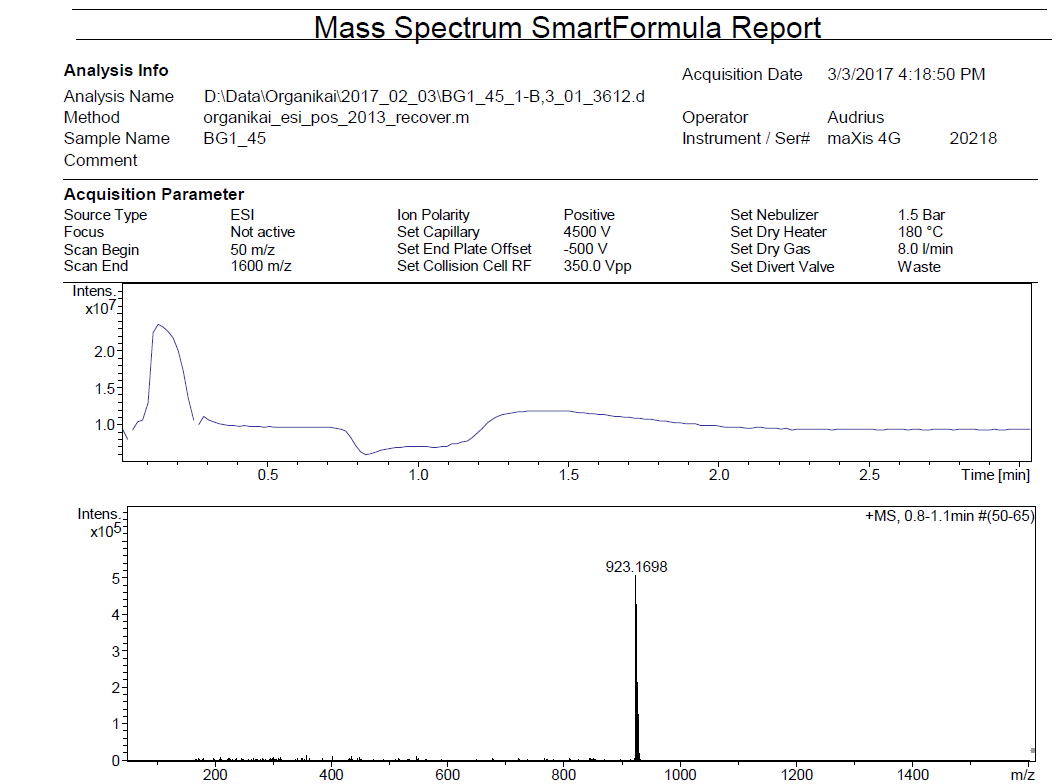


*Figure S21: Mass spectrum of compound* ***24b***

*
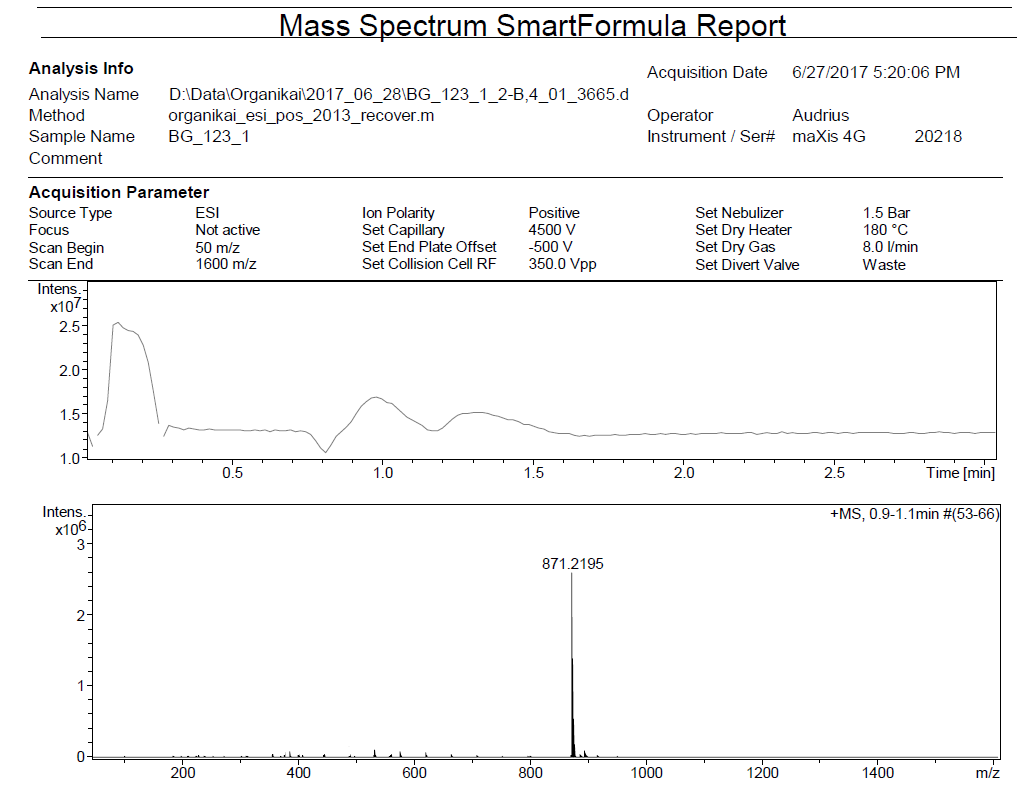
*

*Figure S22: Mass spectrum of compound* ***25b***

***
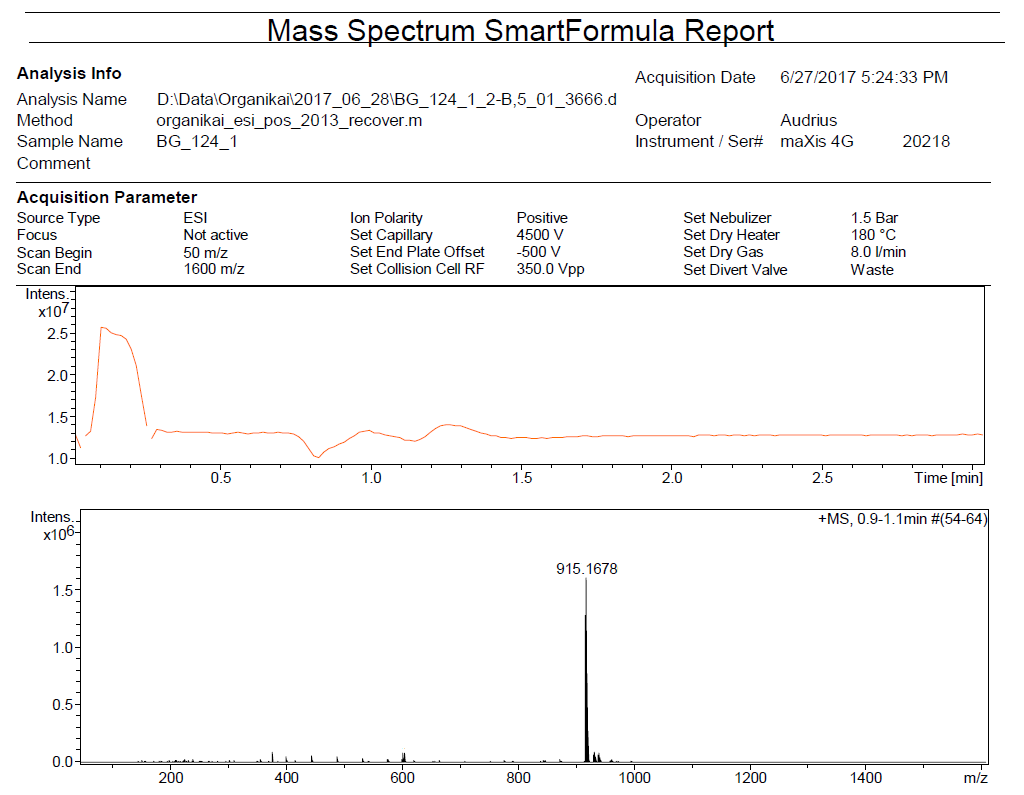
***

*Figure S23: Mass spectrum of compound* ***26b***

***
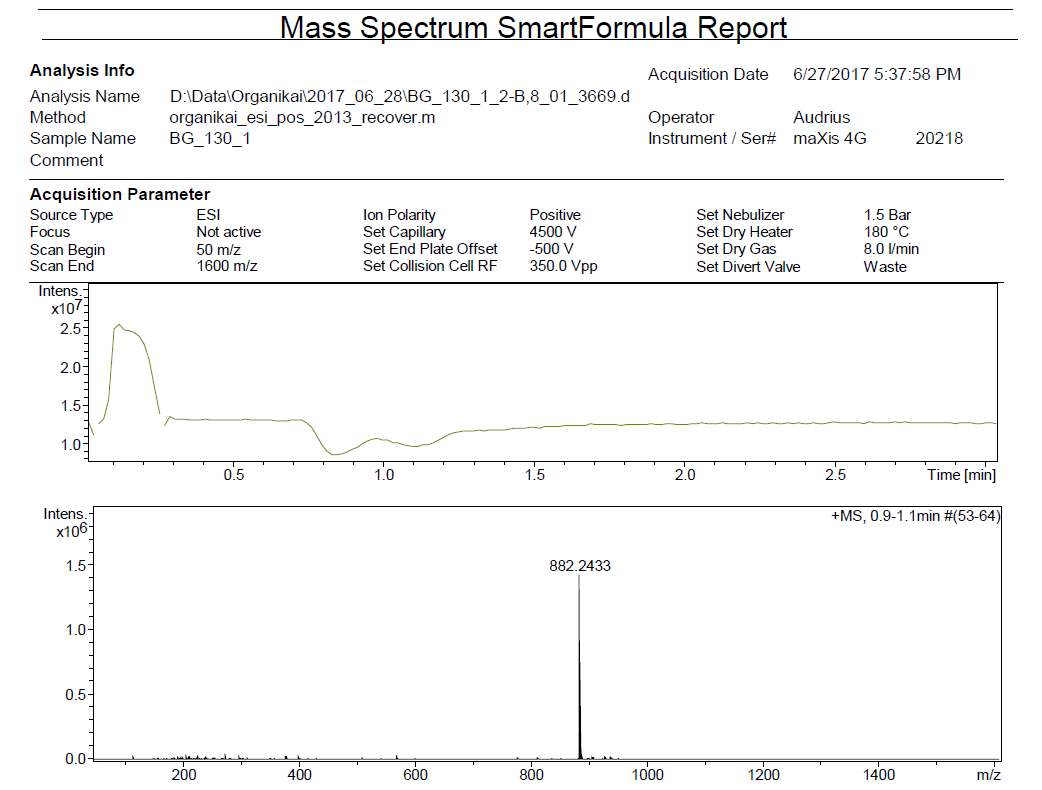
***

*Figure S24: Mass spectrum of compound* ***27b***

***
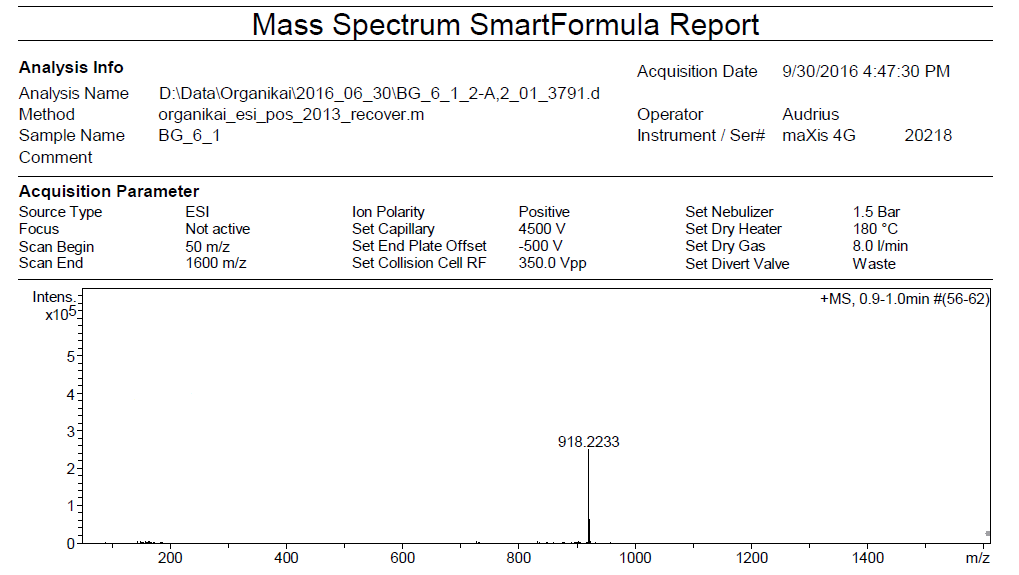
***

*Figure S25: Mass spectrum of compound* ***28b***

***
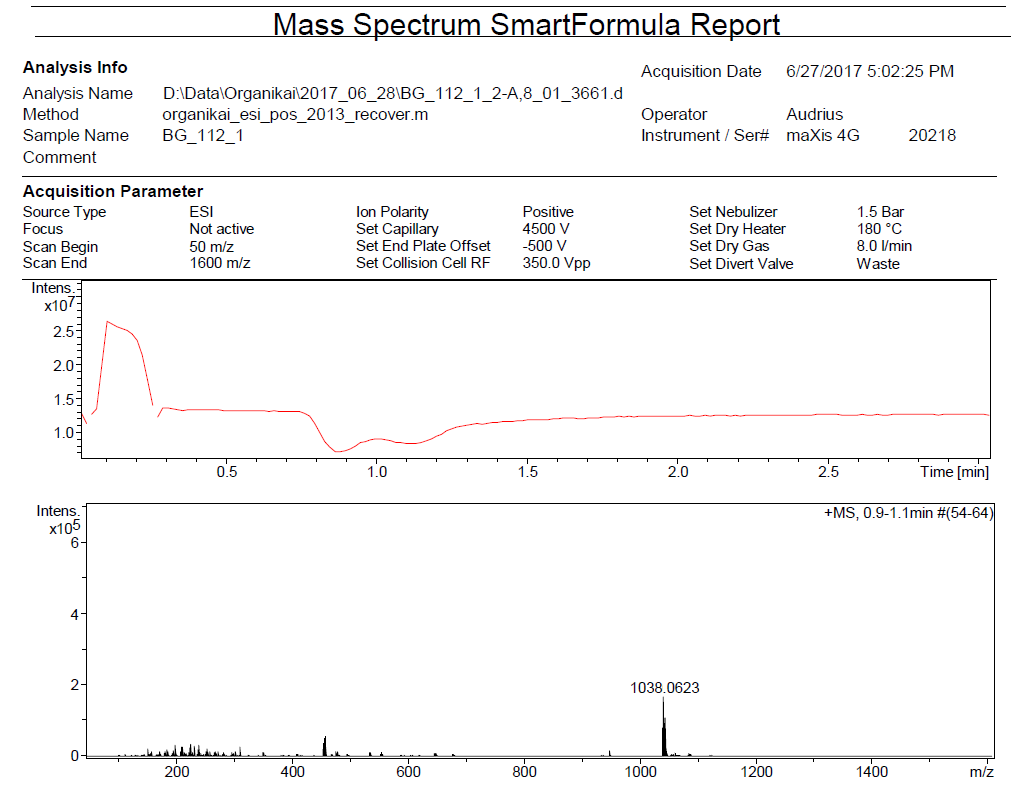
***

*Figure S26: Mass spectrum of compound* ***29b***

***
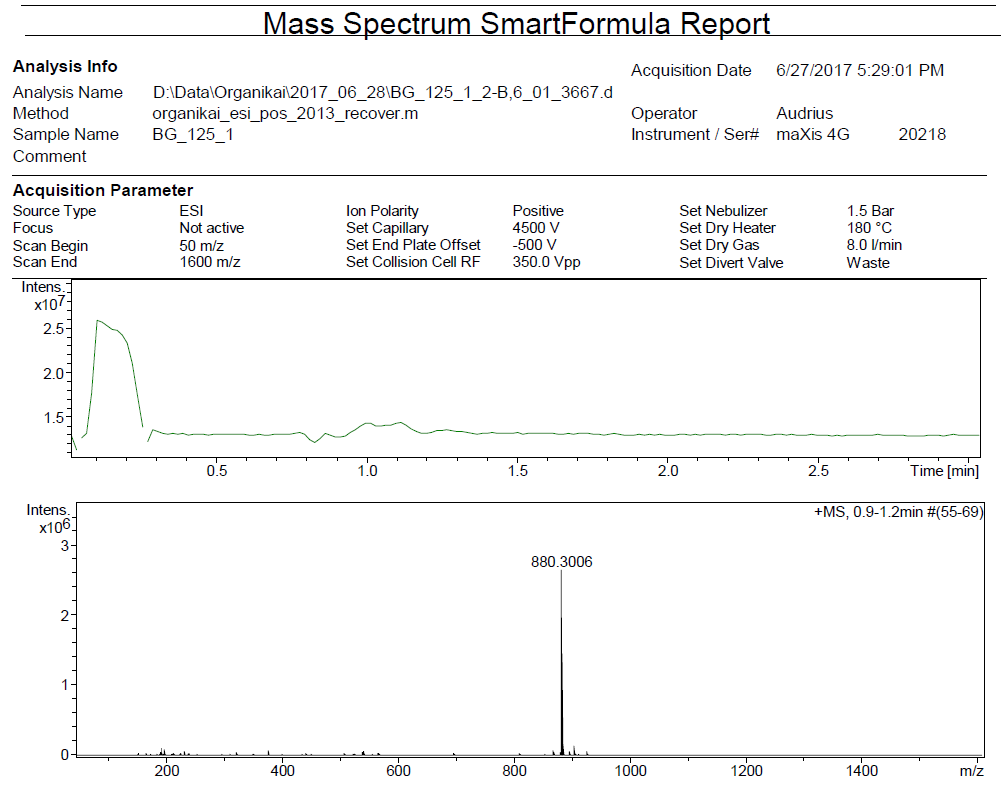
***

*Figure S27: Mass spectrum of compound* ***30b***

***
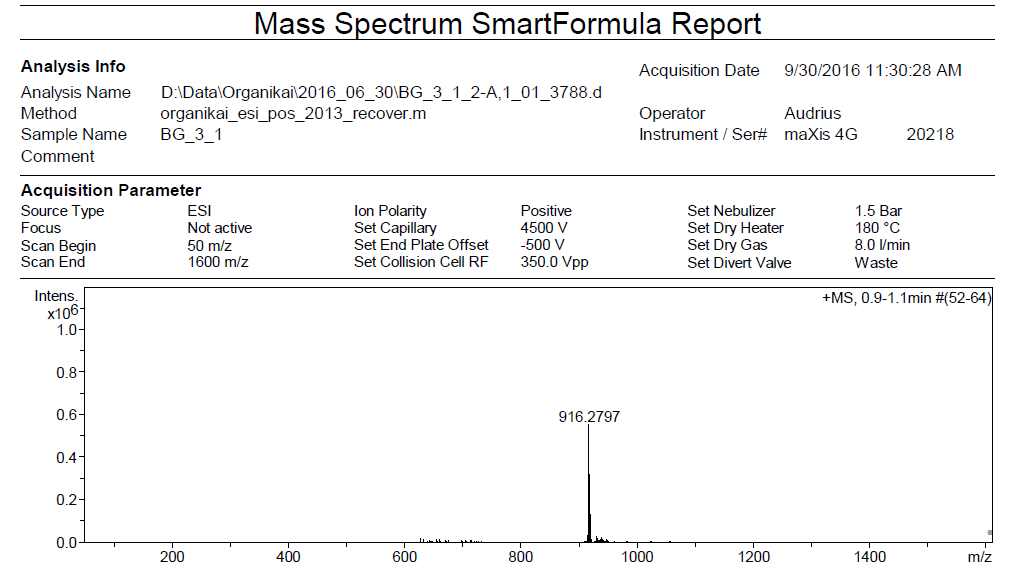
***

*Figure S28: Mass spectrum of compound* ***31b***

1. **IR spectra of synthesized compounds (24-31)b**


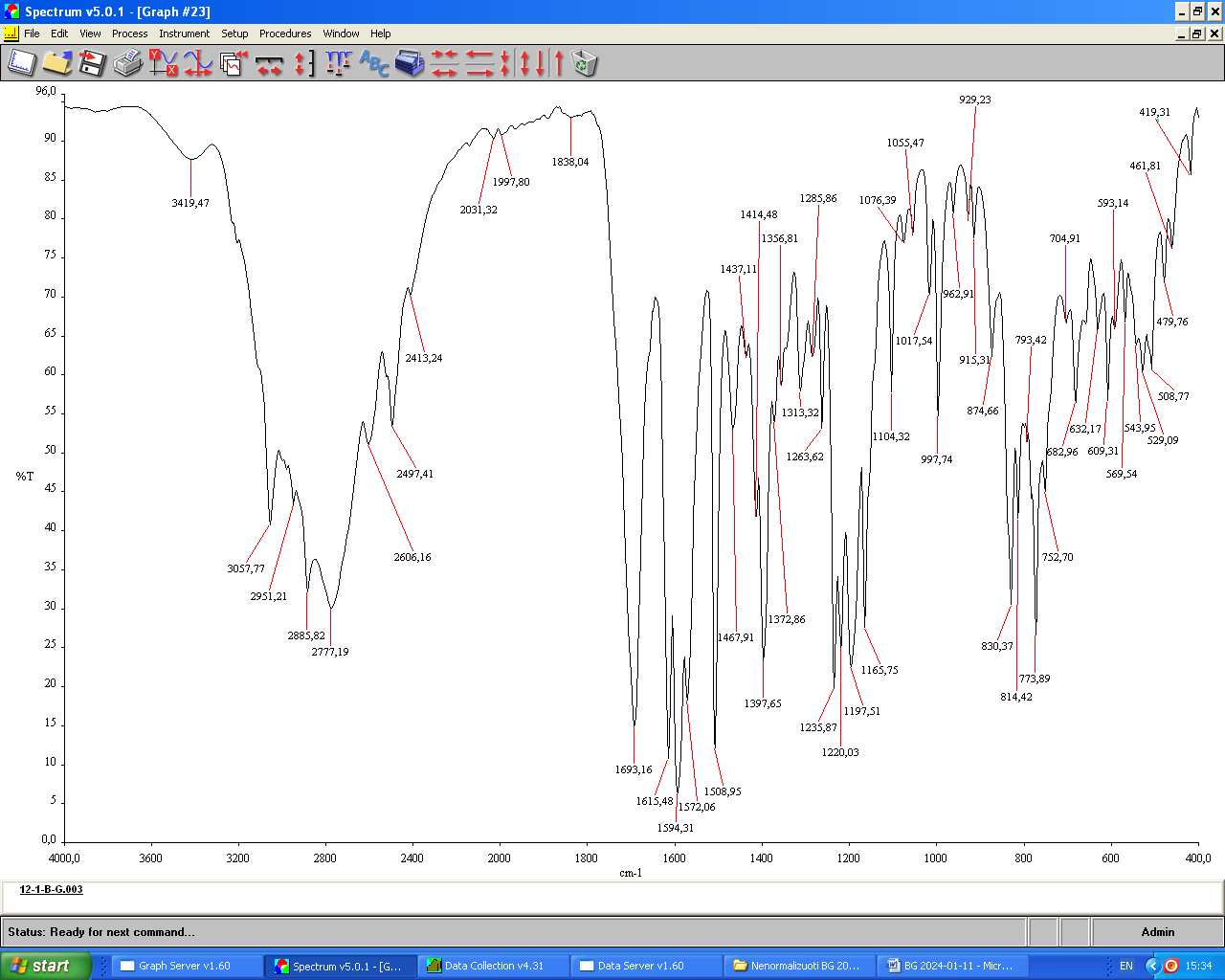


*Figure S29: IR spectra of compound* ***7b***

*Figure S30: IR spectra of compound* ***8b***


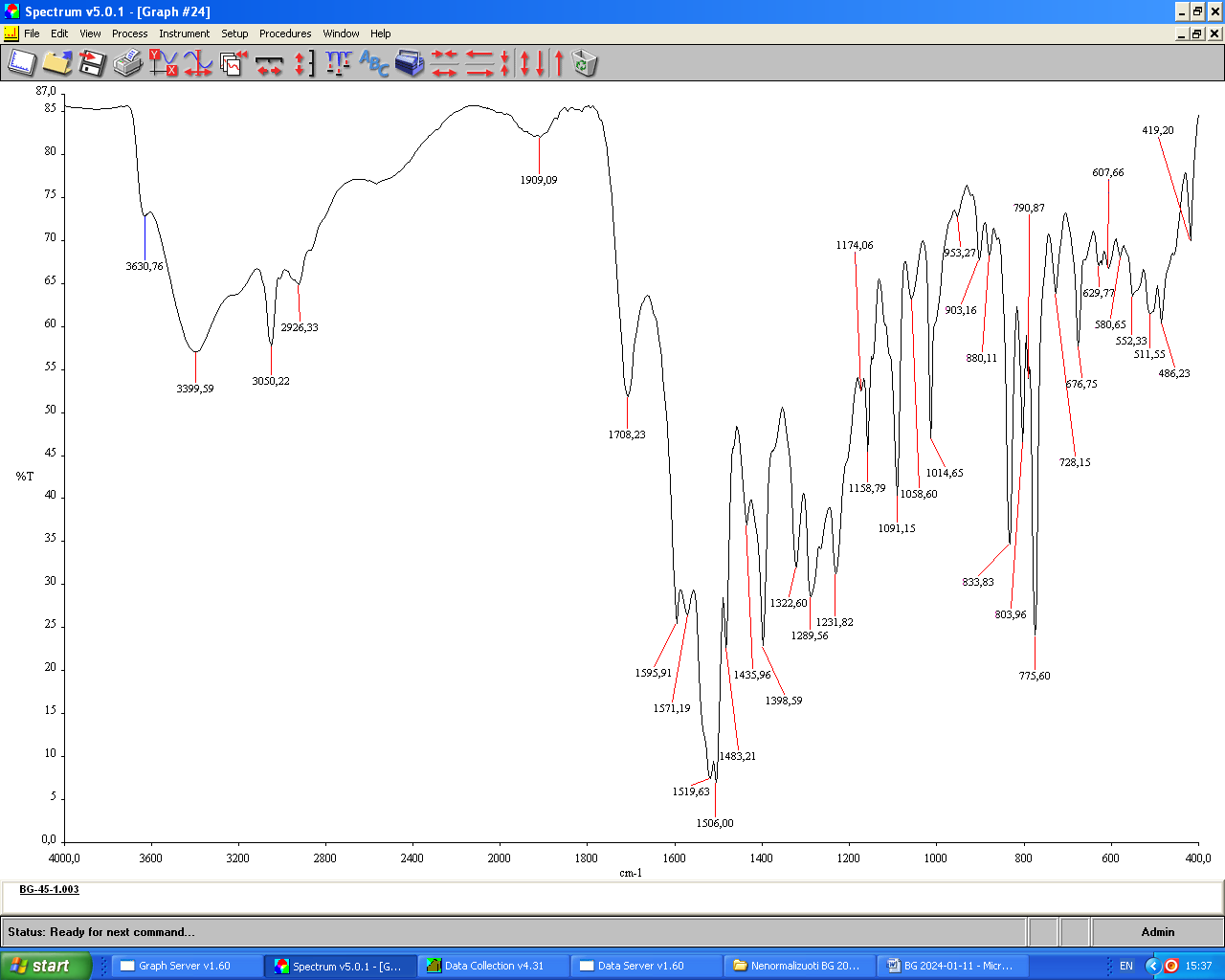


*Figure S31: IR spectra of compound* ***24b***


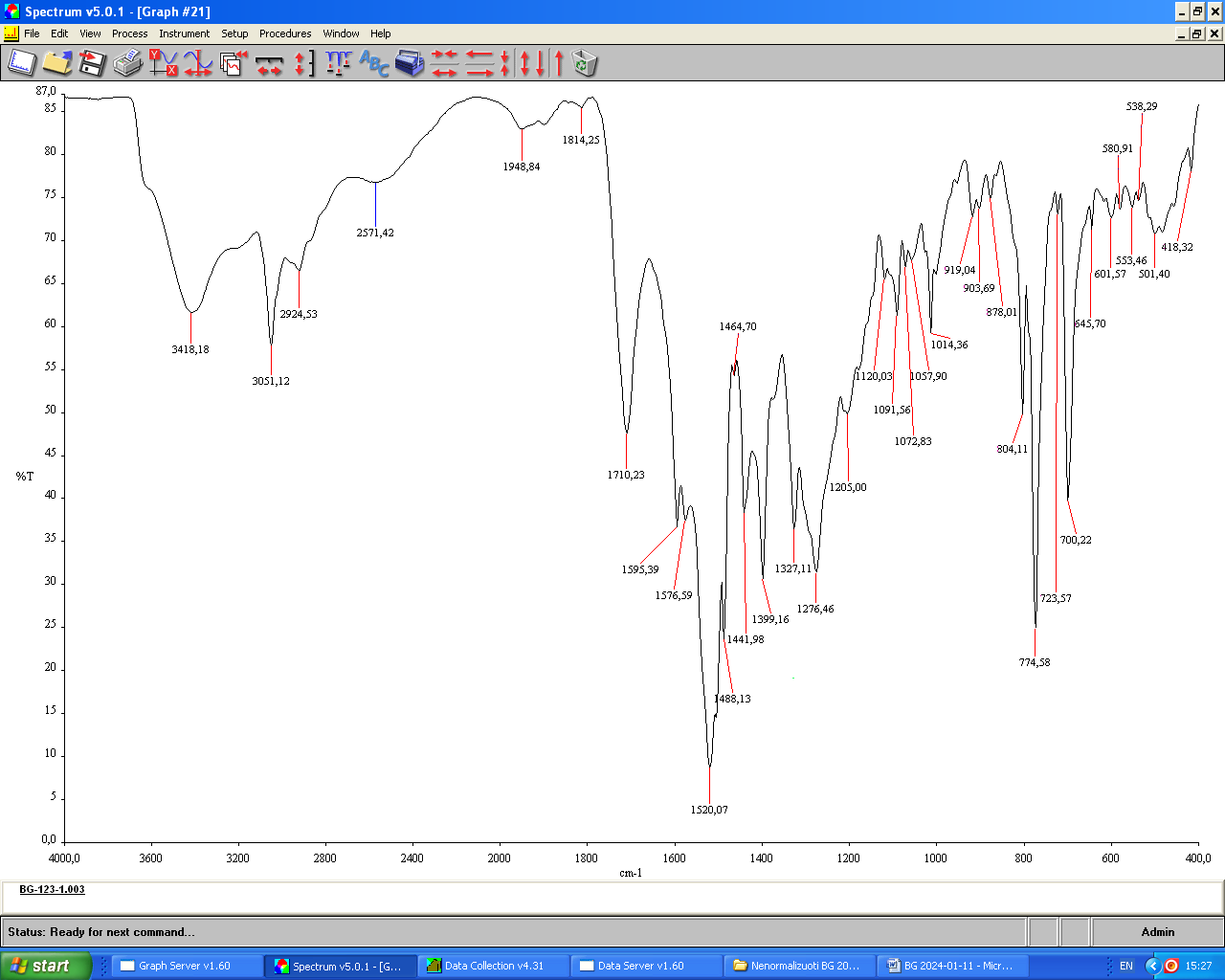


*Figure S32: IR spectra of compound* ***25b***


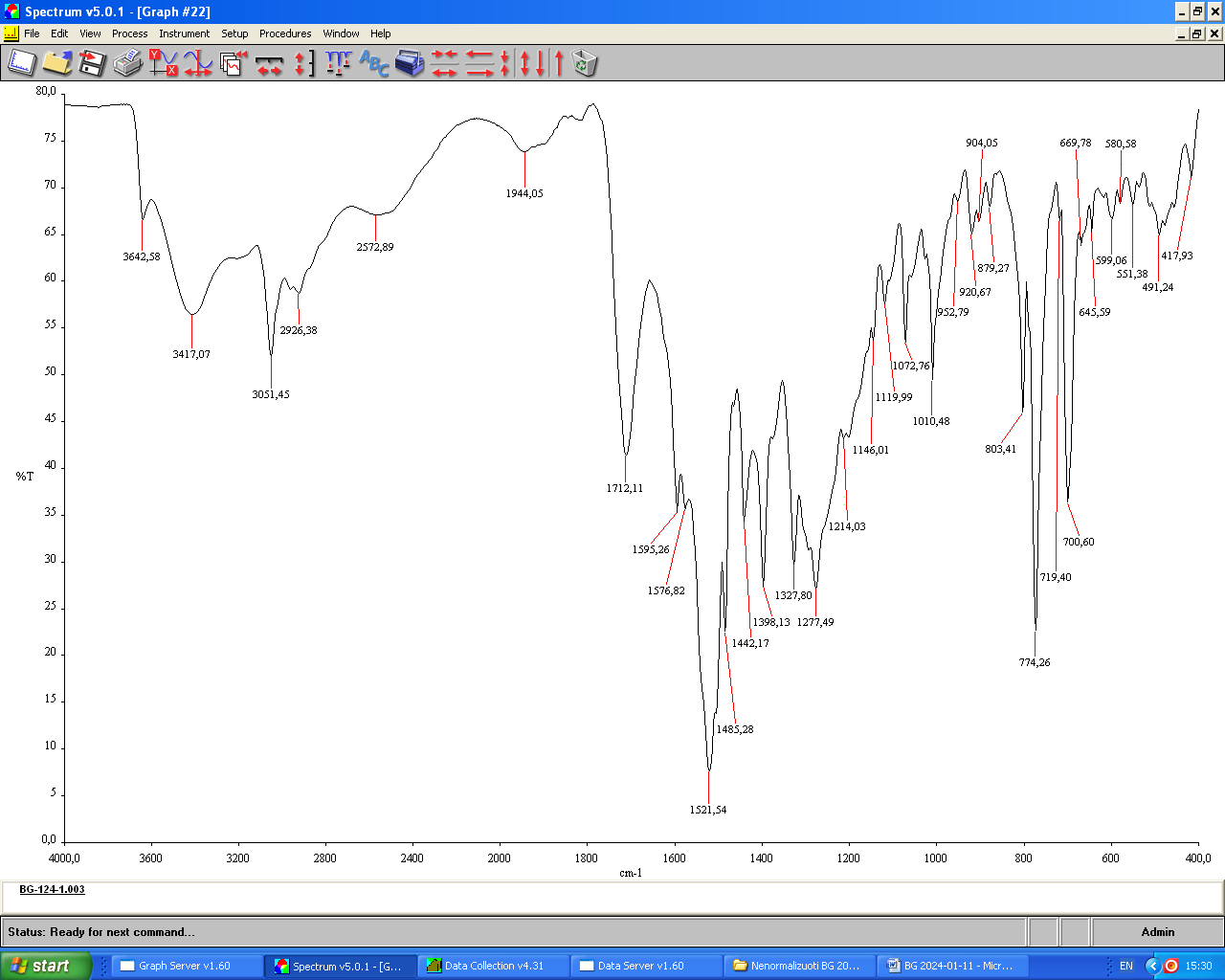


*Figure S33: IR spectra of compound* ***26b***


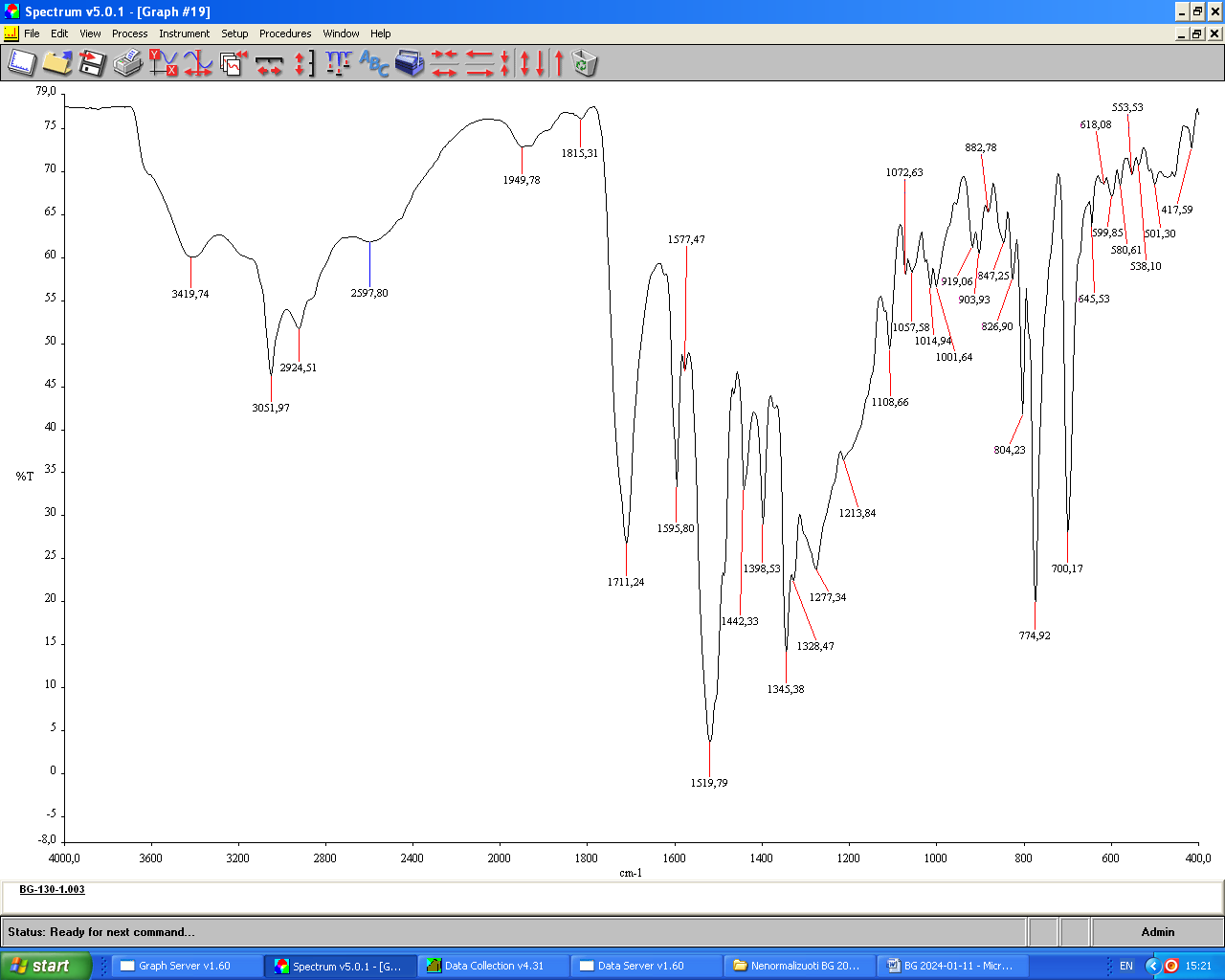


*Figure S34: IR spectra of compound* ***27b***


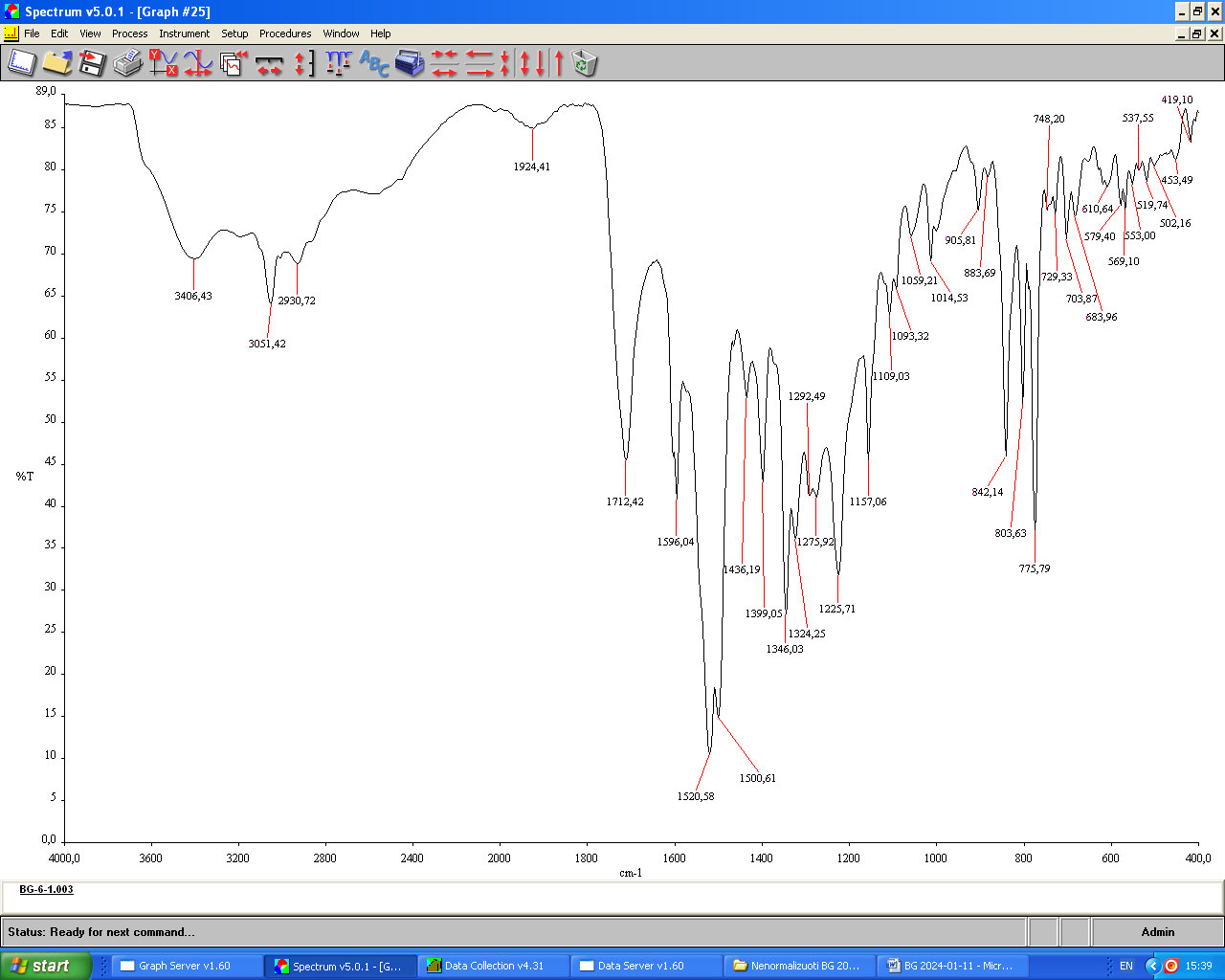


*Figure S35: IR spectra of compound* ***28b***


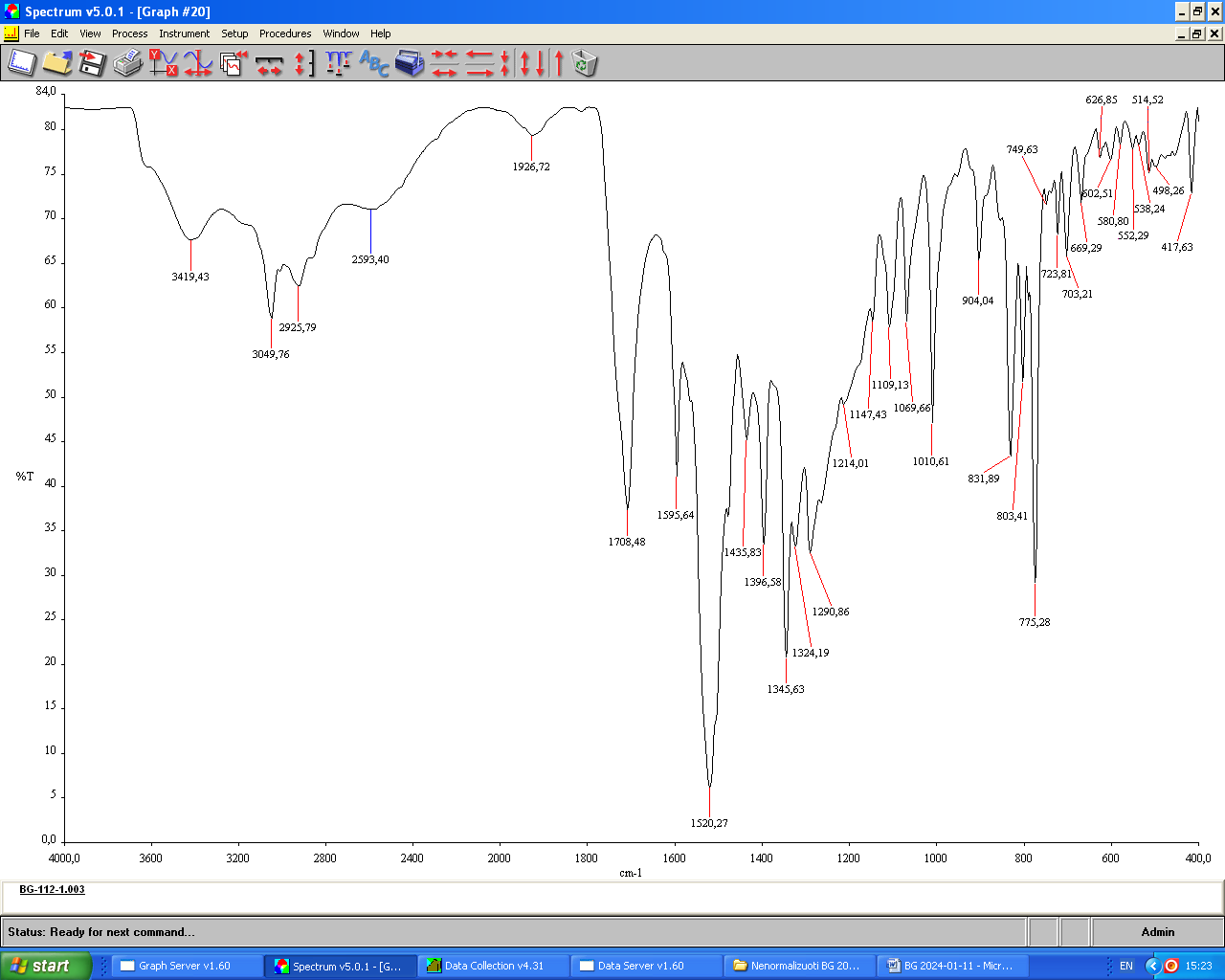


*Figure S36: IR spectra of compound* ***29b***


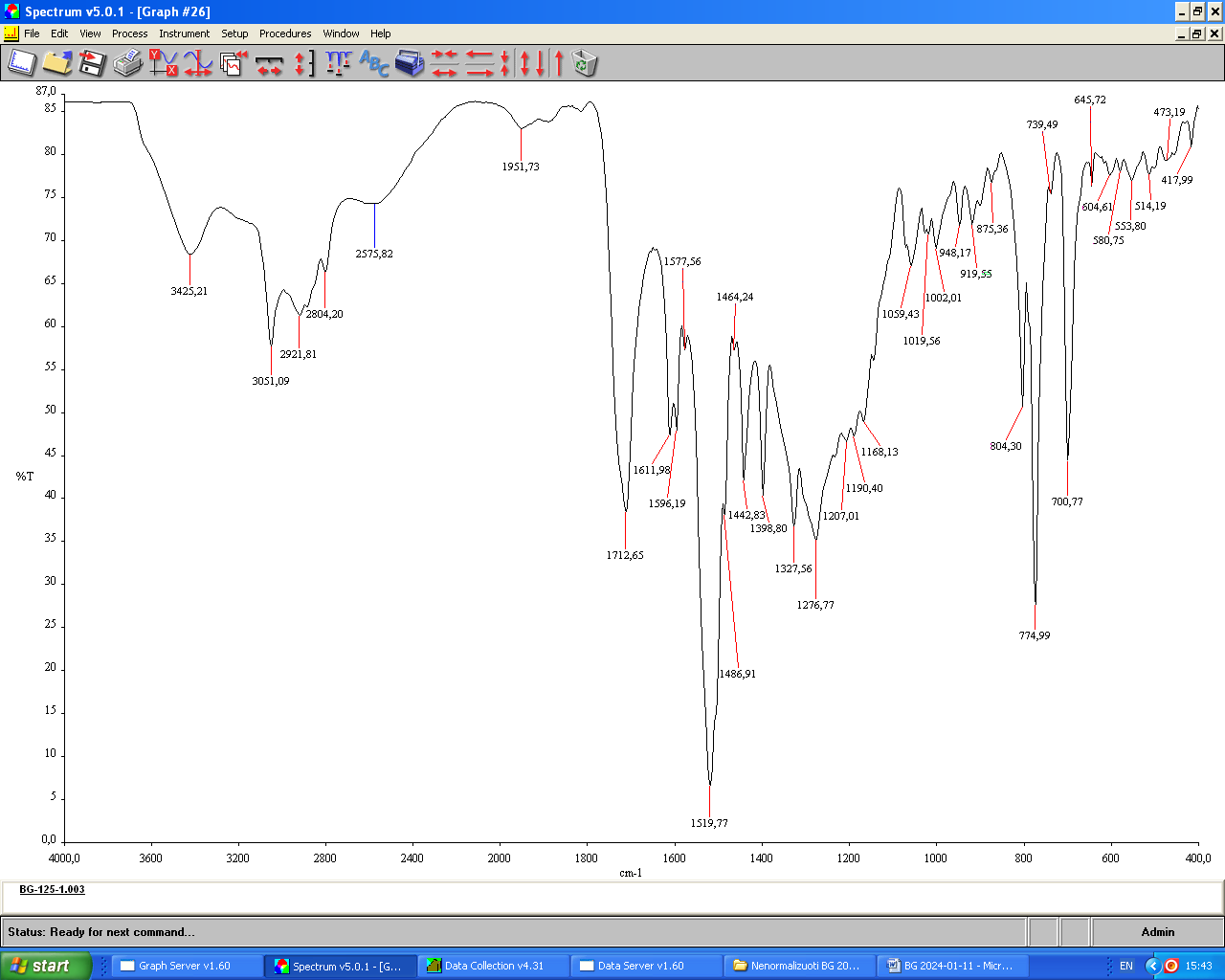


*Figure S37: IR spectra of compound* ***30b***


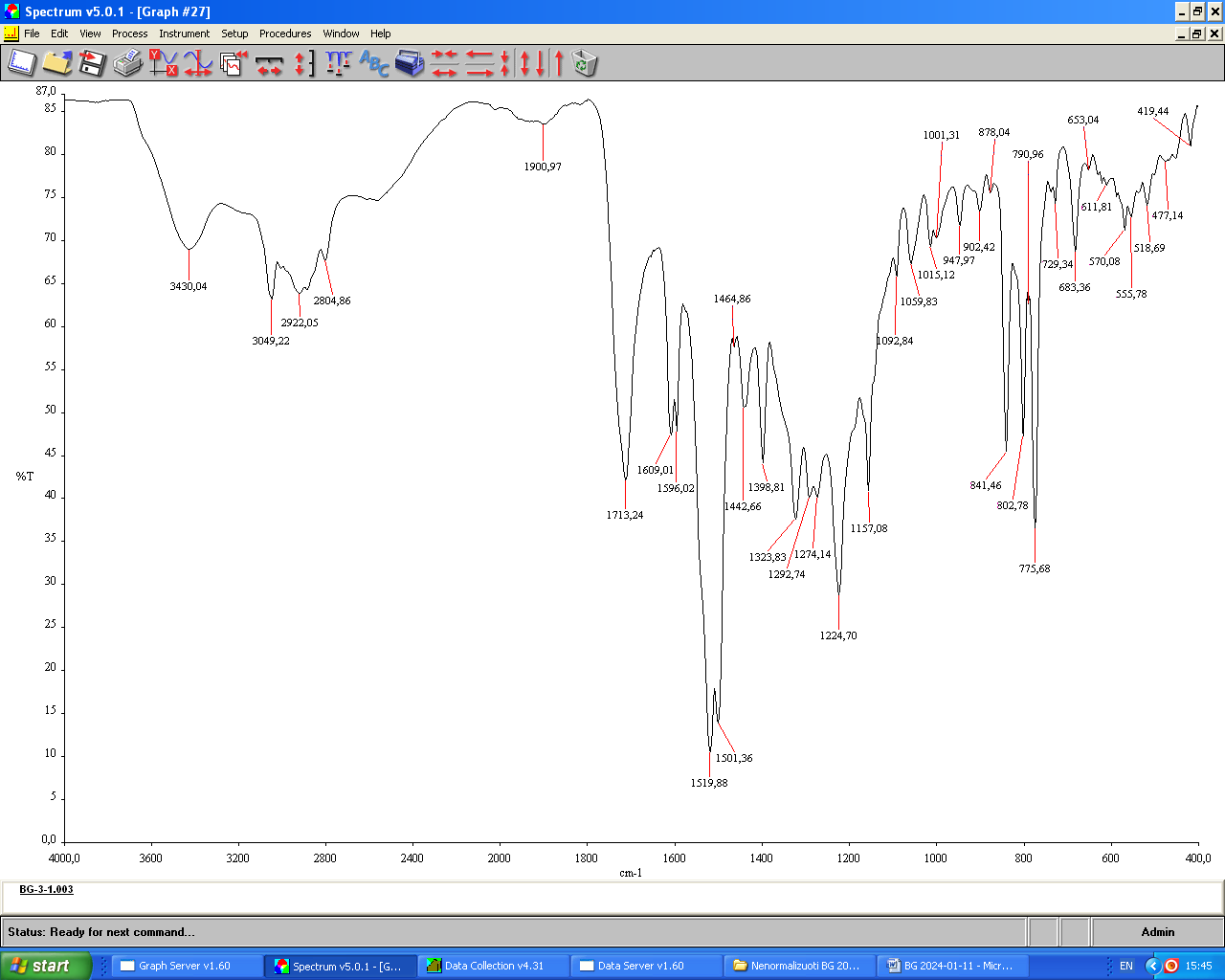


*Figure S38: IR spectra of compound* ***31b***

IV. In vitro antifungal activity

***Figure S39. In vitro antifungal activity of bis(thiazol-5-yl)phenylmethane derivatives 10-31 and control antimicrobial compounds at a concentration of 100 µM against drug-resistant Candida auris AR-0386 strain.*** *C. auris was exposed to compounds and control antimicrobial drugs at a fixed concentration of 100 µM for 18 hours. Subsequently, resazurin (25 µM) was added, and the plates were further incubated for 3 hours. Following incubation, the optical density at 700 nm (OD700 nm) was measured, and the post-treatment viability percentage was normalized to the untreated control (UC). FLU – fluconazole, AmB – amphotericin B. The data presented in the figure represents the mean ± standard deviation (SD) from three independent experimental replicates.*

***Table S1. In vitro minimal bactericidal concentration determination for activity of bis(thiazol-5-yl)phenylmethane derivatives 10-31 and control antimicrobial compounds*.**

| **Compound** | ***S. aureus* 2748 (MSSA)** | | ***S. aureus* TCH 1516 (MRSA)** | | ***S. aureus* VR13 (VRSA)** | |
| --- | --- | --- | --- | --- | --- | --- |
|  | **MIC** | **MBC** | **MIC** | **MBC** | **MIC** | **MBC** |
| **10a** | 4 | 64 | 8 | 64 | 16 | 32 |
| **11a** | 16 | 32 | 32 | 64 | 16 | 32 |
| **12a** | 4 | 64 | 4 | 16 | 4 | 32 |
| **13a** | 8 | 16 | 8 | 16 | 8 | 32 |
| **14a** | 4 | 32 | 16 | 16 | 16 | 64 |
| **15a** | 8 | 16 | 32 | 64 | 16 | 16 |
| **16a** | 8 | 16 | 4 | 32 | 4 | 16 |
| **17a** | 4 | 64 | 4 | 32 | 4 | 16 |
| **18a** | 8 | 64 | 8 | 32 | 8 | 16 |
| **19a** | 4 | 16 | 4 | 32 | 8 | 32 |
| **20a** | 4 | 32 | 16 | 32 | 4 | 32 |
| **21a** | 8 | 32 | 8 | 16 | 4 | 32 |
| **22a** | 64 | 64 | 64 | 64 | 64 | 32 |
| **23a** | 4 | 4 | 4 | 8 | 4 | 4 |
| **24b** | 8 | 16 | 4 | 16 | 4 | 16 |
| **25b** | 16 | 64 | 16 | 8 | 4 | 16 |
| **26b** | 16 | 64 | 8 | 32 | 8 | 32 |
| **27b** | 8 | 32 | 4 | 32 | 4 | 16 |
| **28b** | 2 | 4 | 2 | 4 | 2 | 4 |
| **29b** | 32 | 32 | 16 | 64 | 64 | <64 |
| **30b** | 4 | 16 | 2 | 16 | 2 | 8 |
| **31b** | 4 | 16 | 2 | 8 | 2 | 8 |
| **Clindamycin** | 1 | 2 | 4 | 16 | 4 | 16 |
| **Doxycycline** | 1 | 2 | 8 | 32 | 32 | 64 |
| **Vancomycin** | >1 | >1 | 2 | 8 | 16 | 64 |

**Abreviations:** MIC – minimal inhibitory concentration (*µ*g/mL); MBC – minimal bactericidal concentration (*µ*g/mL).
